# RTF2 controls replication repriming and ribonucleotide excision at the replisome

**DOI:** 10.1038/s41467-024-45947-z

**Published:** 2024-03-02

**Authors:** Brooke A. Conti, Penelope D. Ruiz, Cayla Broton, Nicolas J. Blobel, Molly C. Kottemann, Sunandini Sridhar, Francis P. Lach, Tom F. Wiley, Nanda K. Sasi, Thomas Carroll, Agata Smogorzewska

**Affiliations:** 1 Laboratory of Genome Maintenance, The Rockefeller University, New York, NY 10065 USA; 2 Laboratory for Cell Biology and Genetics, The Rockefeller University, New York, NY 10065 USA; 3 Bioinformatics, The Rockefeller University, New York, NY 10065 USA

**Keywords:** DNA synthesis, Stalled forks

## Abstract

DNA replication through a challenging genomic landscape is coordinated by the replisome, which must adjust to local conditions to provide appropriate replication speed and respond to lesions that hinder its progression. We have previously shown that proteasome shuttle proteins, DNA Damage Inducible 1 and 2 (DDI1/2), regulate Replication Termination Factor 2 (RTF2) levels at stalled replisomes, allowing fork stabilization and restart. Here, we show that during unperturbed replication, RTF2 regulates replisome localization of RNase H2, a heterotrimeric enzyme that removes RNA from RNA-DNA heteroduplexes. RTF2, like RNase H2, is essential for mammalian development and maintains normal replication speed. However, persistent RTF2 and RNase H2 at stalled replication forks prevent efficient replication restart, which is dependent on PRIM1, the primase component of DNA polymerase α-primase. Our data show a fundamental need for RTF2-dependent regulation of replication-coupled ribonucleotide removal and reveal the existence of PRIM1-mediated direct replication restart in mammalian cells.

## Abstract (web summary)

Ribonucleotides are incorporated into DNA during every S phase. Here, the authors show that replisome protein RTF2 localizes RNase H2 to the replisome, promoting ribonucleotide removal by RNase H2 when replication is ongoing but interfering with PRIM1-dependent restart following fork stalling.

## Introduction

Genetic information is duplicated via the highly regulated process of DNA replication. The machinery coordinating this process, the replisome, must faithfully copy the genetic material within the window of S phase. Difficult-to-replicate genomic regions and DNA lesions, including damaged bases and misincorporated nucleotides or ribonucleotides further complicate this essential process^1^. Conditions that slow the progression of replication forks are termed replication stress^2^. Given the crucial nature of DNA replication, the cell has many dedicated molecular mechanisms to deal with replication stress and to ensure that lesions are bypassed and repaired before the transmission of the genetic material^1–3^.

We have previously identified a new mechanism for replication fork stabilization and restart. The proteasome shuttle proteins, DNA Damage Inducible 1 and 2 (DDI1/2), are required to remove Replication Termination Factor 2 (RTF2) from the stalled replisomes, allowing replication restart^4^. However, RTF2’s role in DNA replication and replication stress in mammalian cells remains opaque, and the mechanism of restart affected by RTF2 is unknown. *S. pombe* Rtf2 has been shown to mediate site-specific replication termination by inhibiting replication restart during mating type switching^5^. Recent data, however, show that Rtf2’s function in replication termination is indirect, affecting splicing of Rtf1^6^.

RNase H2 is an enzyme responsible for removing RNA in the context of RNA-DNA heteroduplexes^7,8^. It belongs to the type 2 family of RNase H enzymes, which recognize and cleave the 5’-phosphodiester bond of a single ribonucleotide embedded in a DNA strand in addition to the ability shared with Type I enzymes to remove strings of three or four consecutive DNA-embedded ribonucleotides^9^. RNase H2 comprises three subunits, with RNASEH2A serving as the catalytic core of the complex, while RNASEH2B and RNASEH2C are non-catalytic accessory subunits^10^. RNASEH2B contains a PIP-box thought to localize the RNase H2 complex to PCNA and the replication fork, but RNase H2 may also be recruited to the replication fork independently of the PCNA interaction^10–12^.

The RNase H2 complex is essential for development in mammals and localizes to nascent DNA^13–16^. It plays several roles in DNA replication and replication stress response. Its best-understood function is in the ribonucleotide excision repair (RER) pathway, where RNase H2 removes single DNA-embedded ribonucleotides from the genome, reducing replication stress^8,13^. It has also been proposed to degrade toxic RNA: DNA hybrids behind stalled forks to promote replication restart^17^. Notably, the *S. cerevisiae* homolog of RNase H2 can degrade RNA primers and may contribute to Okazaki fragment maturation during DNA replication^18–22^.

Several pathways promote direct replication restart of the compromised replication fork, including repriming downstream of the lesion, lesion bypass using translesion synthesis (TLS) polymerases, or lesion bypass via template switching (TS)^23–26^. Repriming is dependent on the Archaeo-Eukaryotic Primase (AEP) superfamily of proteins, responsible for synthesizing new primers. In mammals, there are only two identified members of the AEP superfamily: PRIM1 (the primase catalytic subunit of DNA Polymerase α (Pol α)-primase) and PRIMPOL. In *S. cerevisiae*, experiments that reconstituted DNA replication on templates containing fork-stalling lesions showed that Pol α-primase efficiently reprimes replication on the lagging strand but not on the leading strand^27^. The contribution of Pol α-primase-dependent repriming remains to be assessed in response to replication fork stalling in mammalian cells^27^. Recently, PRIMPOL has been implicated in direct replication restart via repriming in mammalian cells, and its function may be most relevant for leading-strand lesion bypass^28–32^.

Here, we show that RTF2 is essential for embryonic development and localizes RNase H2 to nascent DNA. This localization is necessary for proper ribonucleotide excision repair during replication. Moreover, we find that excess of RTF2 at the stalled replication fork leads to inefficient replication restart after treatment with various replication stress-inducing agents. Removal of excess RNase H2 or overexpression of PRIM1 reverses this defect. These data are consistent with our proposed model wherein direct restart of a stalled replication fork is dependent on the ability of PRIM1 to synthesize RNA primers but this repriming can be offset by the presence of increased levels of RNase H2 at the stalled fork when RTF2 is inappropriately present.

## Results

### RTF2 is necessary for in vivo viability, cellular proliferation, and DNA replication

**Fig. 1:**
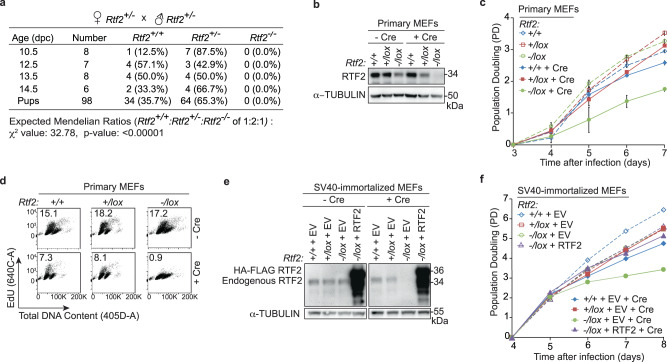
RTF2 is necessary for viability and cellular proliferation. **a** Genotypes from litters of *Rtf2*^*+/-*^ female mice crossed with *Rtf2*^*+/-*^ male mice showing embryonic lethality. **b** Representative immunoblot of whole cell lysates showing RTF2 levels 72 hrs after transduction of MEFs with Hit & Run pMMP Cre retrovirus^65^. α-tubulin represents loading control. **c** Representative growth curves in MEFs transduced with pWZL Cre-hygro retrovirus. **d** Representative cell cycle profiles from flow cytometry at 72 hrs after Cre. Percentage of S phase cells is indicated. **e** Representative immunoblot of whole cell lysates for RTF2 deletion in *Rtf2*^*-/lox*^ SV40-immortalized MEFs expressing empty vector (EV) or HA-FLAG-mRTF2 (RTF2) cDNA constructs transduced with pWZL Cre-hygro retrovirus 120 hrs before harvest. α-tubulin represents loading control. **f** Representative growth curves of MEFs 72 hr after transduction with Hit & Run pMMP Cre expressing empty vector (EV) or HA-FLAG-mRTF2 (RTF2) cDNA constructs^65^. Error bars represent standard deviation for (**c**). Experiments were conducted at least three times in biological replicates with consistent results for (**b**)–(**f**). Source data are provided as a Source Data file.

To investigate the function of RTF2, we intercrossed *Rtf2*^*+/-*^ and *Rtf2*^*+/stop*^ mice (Supplementary Fig. 1a–c). While heterozygous mice were viable and grew at rates comparable to those of wild type mice, neither *Rtf2*^*stop/stop*^ nor *Rtf2*^*-/-*^ offspring were observed, indicating that RTF2 is essential for embryonic development (Fig. 1a, Supplementary Fig. 1d, e). Primary *Rtf2*^*-/lox*^ mouse embryonic fibroblasts (MEFs) treated with Cre recombinase proliferated slowly with few cells in S phase (Fig. 1b–d, Supplementary Fig. 2a). This phenotype was recapitulated in SV40-immortalized *Rtf2*^*-/lox*^ MEFs and a clonal *Rtf2*^*-/-*^ cell line, and was rescued by complementation with expression of mRTF2 (Fig. 1e, f, Supplementary Fig. 2b–g). Based on the in vivo and ex vivo data, we conclude that RTF2 is required for organismal and cellular growth.

RTF2-deficient cells exhibited abnormal nuclear morphology, including multinucleated cells and cells with multiple micronuclei (Supplementary Fig. 2h, i). Live cell imaging of RTF2-deficient cells expressing hGFP-H2B revealed an increased percentage of abnormal mitoses in the absence of RTF2 (Supplementary Fig. 2j, k, Movies 1–3). The accumulation of abnormal nuclei was suppressed by treatment with RO-3306, a potent inhibitor of CDK1 that arrests cells in G2 and prevents entry into M phase (Supplementary Fig. 2l, m), underscoring that the increase in abnormal nuclei is the result of aberrant cellular division^33^.

**Fig. 2:**
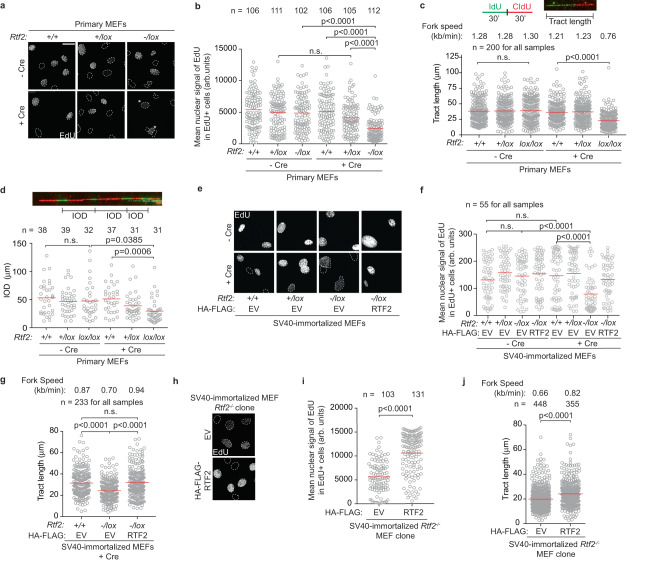
RTF2 is necessary for DNA replication in primary and immortalized MEF cell lines. **a** Representative immunofluorescence of primary MEFs after infection with Hit & Run Cre 72 hrs before fixation^65^. Nuclei are outlined in dashed lines based on DAPI staining. Asterisks indicate EdU-positive cells in the *Rtf2*^*-/lox*^ + Cre sample. **b** Quantification of representative experiment of mean nuclear signal of EdU from EdU-positive cells at 72 hrs after Cre. **c** Top: Schematic and representative image of DNA combing. PBS washes are indicated by a black vertical line. Below: Quantification of representative experiment of replication tract lengths from primary MEFs at 72 hrs after Cre. **d** Top: Representative image of inter-origin distances measured within replication clusters. Bottom: Quantification of representative experiment of inter-origin distances (IOD) from primary MEFs at 72 hrs after Cre. **e** Representative immunofluorescence of SV40-immortalized MEFs expressing empty vector (EV) or HA-FLAG-mRTF2 (RTF2) cDNA constructs at 120 hrs after Cre. Nuclei are outlined in dashed lines based on DAPI staining. **f** Quantification of mean nuclear signal of EdU from EdU-positive cells from (**e**). **g** Quantification of representative experiment of replication tract lengths from SV40-immortalized MEFs stably expressing HA-FLAG empty vector (EV) or Rtf2 at 120 hr after Cre. **h** Representative immunofluorescence images of MEFs. Nuclei are outlined in dashed lines based on DAPI staining. **i** Quantification of representative experiment of mean nuclear signal of EdU from EdU-positive SV40-immortalized *Rtf2*^*-/-*^ sub-cloned MEFs expressing empty vector (EV) or HA-FLAG-mRTF2 (RTF2) cDNA constructs. **j** Quantification of representative experiment of replication tract lengths of progressing fork species in SV40-immortalized *Rtf2*^*-/-*^ sub-cloned MEFs expressing empty vector (EV) or HA-FLAG-mRTF2 (RTF2) cDNA constructs. Experiments were conducted at least three times in biological replicates with consistent results for (**a**)–(**j**). Cells were pulsed with EdU for 1 hr prior to fixation for (**a**), (**b**), (**e**), (**f**), (**h**), (**i**). Each dot represents one EdU-positive cell in (**b**), (**f**), (**i**). Mean is indicated with a red line for (**b**)-(**d**), (**f**), (**g**), (**i**), (**j**). Experiments were blinded prior to analysis for (**b**)–(**d**), (**f**), (**g**), (**i**), (**j**). Average fork speed listed above each sample for (**c**), (**g**), (**j**). Outliers removed with ROUT (1%) for (**c**), (**g**), (**j**). Significance evaluated by Kruskal-Wallis ANOVA with a Dunn’s post-test. Source data are provided as a Source Data file.

Given that RTF2 is a component of the replisome, we hypothesized that aberrant cellular division in the setting of RTF2 loss could follow aberrant DNA replication^4,34,35^. We assessed cell cycle profiles of cells lacking RTF2 with 5-ethynyl-2′-deoxyuridine (EdU) labeling. Cre-treated primary *Rtf2*^*-/lox*^ MEFs in S phase had lower peak EdU intensity (Fig. 2a, b). DNA combing revealed a significant decrease in replication speed and shortening of the inter-origin distance in RTF2-deficient cells (Fig. 2c, d). These findings were recapitulated in SV40-immortalized *Rtf2*^*-/lox*^ MEFs and a clonal *Rtf2*^*-/-*^ cell line, and the replication speed defect was complemented by expression of mRTF2 cDNA (Fig. 2e–j). The replication tracks emanating bidirectionally from an origin of replication were symmetrical (Supplementary Fig. 3), suggesting that, though slow-moving, RTF2-deficient replication forks are stable.

Previous reports in *A. thaliana* suggest RTF2 has a role in intron retention, likely through non-conserved N-terminal extension in the atRTF2 protein^36^. Similarily, *S. pombe* mutant of Rtf2 displayed intron retention^6^. However, we did not detect any significant changes in global intron retention, and the transcriptional profiles of RTF2-deficient MEFs were largely unchanged (Supplementary Fig. 4a–c).

### RTF2 function during unperturbed replication is dependent on RNase H2

**Fig. 3:**
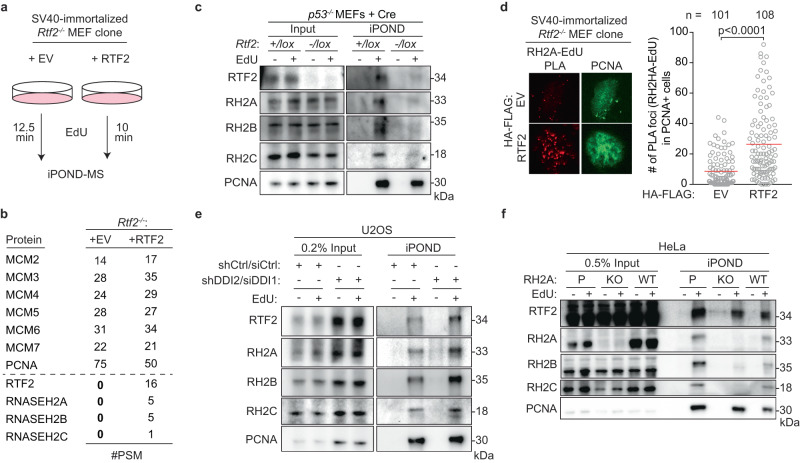
RTF2 regulates RNase H2 localization to the replisome. **a** Schematic of the iPOND set-up in SV40-immortlized *Rtf2*^*-/-*^ MEFs expressing HA-FLAG empty vector (EV) or RTF2 cDNA. EdU pulses were normalized to replication tract lengths. Nascent DNA was purified using streptavidin beads against biotin-conjugated EdU. **b** Peptide Spectral Match (#PSM) values for indicated proteins from experiment in (**a**). **c** Representative iPOND immunoblot from *p53*^*-/-*^ MEFs at 72 hr after Cre. **d** Left: Representative images of RNASEH2A-EdU nascent proximity ligation assay (nPLA) co-stained with PCNA in SV40-immortalized *Rtf2*^*-/-*^ MEFs expressing HA-FLAG EV or RTF2 cDNA. Right: Quantification of RNASEH2A-EdU foci in PCNA-positive cells. **e** Representative iPOND immunoblot in DDI-depleted U2OS cells. Proteins purified using streptavidin beads recognizing nascent DNA were detected by immunoblot. **f** Representative iPOND immunoblot in CRISPR-edited RNASEH2A KO or WT HeLa cells. Proteins purified using streptavidin beads recognizing nascent DNA were detected by immunoblot. Experiments were conducted at least three times in biological replicates with consistent results for (**c**)–(**f**). Mean is shown with red line for (**d**). Significance was evaluated by Kruskal-Wallis ANOVA with a Dunn’s post-test. P = Parental, RH2A = RNASEH2A, RH2B = RNASEH2B, RH2C = RNASEH2C, RH2 = RNASEH2 complex, Ctrl = Control. Source data are provided as a Source Data file.

To identify the mechanism responsible for the observed replication defect, we employed isolation of proteins on nascent DNA (iPOND) to examine the changes in the replication fork proteome in the absence of RTF2 (Fig. 3a)^37^. While many replisome components were unchanged, peptides from the RNase H2 complex were lost from replication forks in RTF2-deficient MEFs (Fig. 3b, c, Supplementary Data 1). Nascent DNA proximity ligation assay (nPLA), an orthogonal method to detect the presence of proteins at nascent DNA^38^, showed a dramatic decrease of endogenous RNASEH2A, the catalytic subunit of RNase H2, at the replication fork in the absence of RTF2 (Fig. 3d, Supplementary Fig. 5a). The decrease in RNASEH2A at the replication fork is not due to loss of *Rnaseh2a* transcripts or total protein levels (Supplementary Fig. 5b–d). Increasing the levels of RTF2 at replication forks through DDI1/2 depletion led to increased levels of RNase H2 at nascent chromatin (Fig. 3e)^4^. RNASEH2A loss, however, did not result in loss of RTF2 from replication forks (Fig. 3f).

**Fig. 4:**
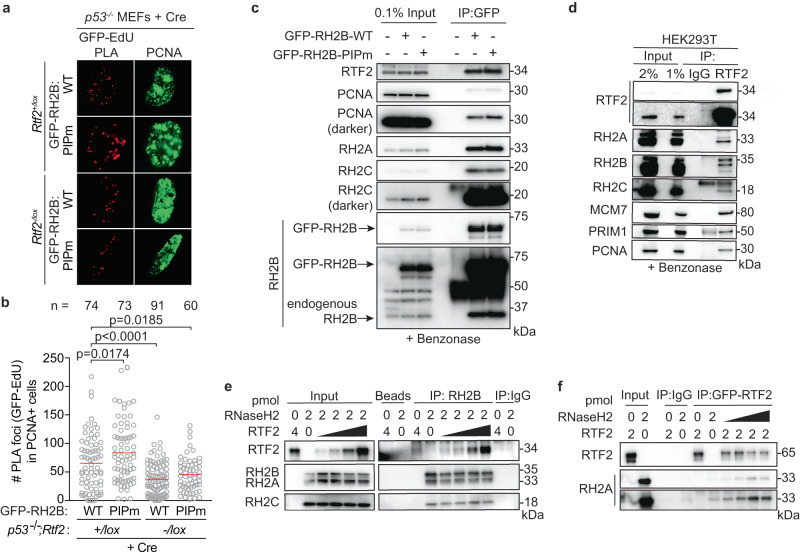
RTF2 directly interacts with RNase H2 for localization at the replisome, independent of RNaseH2B’s PIP-box. **a** Representative images from GFP-EdU nPLA co-stained with PCNA in *p53*^*-/-*^ MEFs expressing GFP-tagged wildtype (WT) or PIP box mutant (PIPm, RNASEH2B^F300A;F301A^) RNASEH2B at 72 hr after Cre. **b** Quantification of GFP (RNASEH2B)-EdU foci in PCNA-positive cells. Note that endogenous RNase H2 is present in these cells. **c** Representative immunoblots following GFP immunoprecipitation of exogenously-expressed GFP-RNASEH2B-WT or GFP-RNASEH2B-PIP mutant from HEK293T cells. **d** Representative immunoblots following immunoprecipitation of RTF2 from HEK293T cells. **e** Representative immunoblot from immunoprecipitation of recombinant RNase H2 complex and RTF2 expressed in *E. coli*. Protein amount (pmol) indicated above each lane; range of RTF2 is 0.5, 1, 2, 4 pmol. Images for input and pulldown of RTF2 are separate exposures of the same blot. **f** Representative immunoblot from immunoprecipitation of recombinant GFP-tagged RTF2 and RNase H2 complex expressed in *E. coli*. Protein amount (pmol) indicated above each lane; range of RTF2 is 0.5, 1, 2, 4 pmol. Experiments were conducted at least three times in biological replicates with consistent results for (**a**)–(**f**). Mean is shown with red line for (**b**). Significance was evaluated by Kruskal-Wallis ANOVA with a Dunn’s post-test. WT = wildtype, PIPm = PIP box mutant, RH2A = RNASEH2A, RH2B = RNASEH2B, RH2C = RNASEH2C. Source data are provided as a Source Data file.

RNase H2 has been previously reported to localize to the replication fork through its PCNA-interacting protein-box (PIP-box) in the RNaseH2B subunit^11,12^. Using cells overexpressing WT or PIP-box mutated RNASEH2B (RNASEH2B^F300A;F301A^), we asked if RNaseH2B localization to nascent DNA was dependent on the intact PIP box and/or RTF2. nPLA revealed slightly higher numbers of RNASEH2B foci in cells that expressed RTF2 (*p53*^*-/-*^*;Rtf2*^*+/lox*^ MEFs + Cre), when PIP box was mutated. However, the number of nPLA foci diminished when RTF2 was absent (*p53*^*-/-*^*;Rtf2*^*-/lox*^ MEFs + Cre), and the extent of the decrease was similar for the WT and PIP-mutant RNASEH2B (Fig. 4a, b, Supplementary Fig. 5e, f). In combination with the above data showing that endogenous RNase H2 no longer localizes to nascent DNA in the absence of RTF2, we conclude that RTF2 is a major regulator of RNase H2 localization to the replisome. Moreover, RNASEH2B^F300A;F301A^ and PCNA co-immunoprecipitated to the same extent as wildtype RNASEH2B, suggesting that the RNASEH2B-PCNA interactions are by and large indirect in this context (Fig. 4c)^12,13^.

To test if RNASEH2A and RTF2 were found in the same complexes in cells, we used endogenously- or exogenously-tagged RTF2 to perform immunoprecipitations and LC-MS analysis. Indeed, they were found to interact (Supplementary Fig. 6a–h, Supplementary Data 2 and 3), and the interactions were confirmed by co-immunoprecipitation of endogenous proteins (Fig. 4d). Immunoprecipitations of recombinant proteins purified from *E.coli* showed direct interaction between RTF2 and RNase H2 (Fig. 4e, f, Supplementary Fig. 6i)^39^. Together, these data show that RTF2 localizes the RNase H2 complex to nascent DNA through a direct interaction. Like RTF2 itself, RNase H2 levels at replication forks are dynamically regulated by the DDI proteins.

**Fig. 5:**
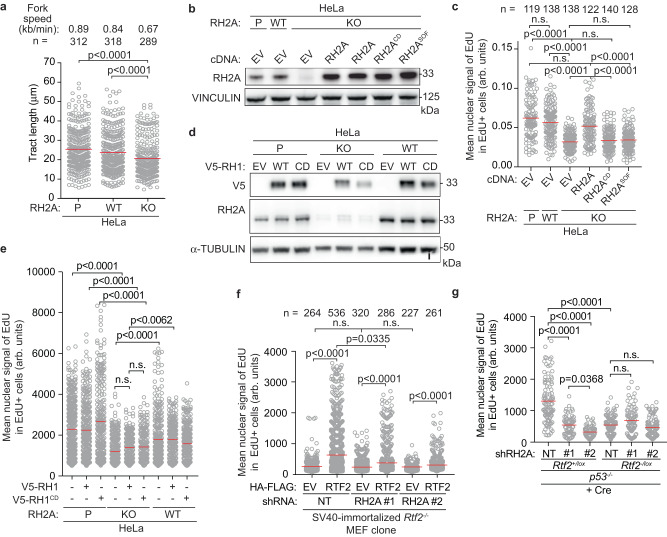
RTF2 recruits RNase H2 to the replisome to facilitate DNA replication. **a** Quantification of representative experiment of replication tract lengths in CRISPR-edited RNASEH2A knockout (KO) or wildtype (WT) HeLa cells. **b** Representative immunoblot showing complementation of wildtype, catalytic dead (RH2A^CD^/RNASEH2A^D34A;D169A^), or separation of function (RH2A^SOF^/RNASEH2A^P40D;Y210A^) RNASEH2A in CRISPR-edited RNASEH2A KO HeLa cells. Vinculin represents loading control. **c** Quantification of representative experiment of mean signal of EdU in EdU-positive cells in indicated cells. **d** Representative immunoblot showing expression of empty vector (EV), wildtype V5-RNASEH1, or catalytic dead V5-RNASEH1^D210N^ in CRISPR-edited RNASEH2A KO or HeLa cells. α-tubulin represents loading control. **e** Quantification of representative experiment of mean signal of EdU in EdU-positive cells in indicated cells. **f** Quantification of mean nuclear signal of EdU in EdU-positive SV40-immortalized MEFs, combined from 4 biological replicates. **g** Quantification of representative experiment of mean signal of EdU in EdU-positive cells in indicated cells. Experiments were conducted at least three times in biological replicates with consistent results for (**a**)–(**g**). Average fork speeds are listed above each sample for (**a**). Outliers removed with ROUT (1%) for (**a**). Each dot represents one cell for (**c**), (**e**), (**f**), (**g**). Mean for each sample shown with red line for (**a**), (**c**), (**e**), (**f**), (**g**). Cells were pulsed with EdU for 1 hr prior to fixation for (**c**), (**e**), (**f**), (**g**). Experiments were blinded prior to analysis for (**a**), (**c**), (**e**), (**f**), (**g**). Significance was evaluated by Kruskal-Wallis ANOVA with a Dunn’s post-test. RH2A RNASEH2A, P Parental, WT wildtype, EV empty vector, RH2A^CD^ = catalytic dead RNASEH2A^D34A/D169A^, RH2A^SOF^ = separation of function RNASEH2A^P40D/Y210A^, RH1 RNASEH1, CD catalytic dead RNASEH1^D210N^, Ctrl Control, NT Non-targeting. Source data are provided as a Source Data file.

We next evaluated whether RNase H2 deficiency phenocopies RTF2 deficiency, which would be consistent with RTF2 regulating RNase H2 localization to the replication fork. Like RTF2-deficient cells, RNASEH2A knockout (KO) cells displayed slower replication speed (Fig. 5a). Additionally, *RNASEH2A*^-/-^;*p53*^*-/-*^ HCT-116 cells, *RNASEH2A*^-/-^ HeLa cells, and *RNASEH2A*-depleted BJ cells, exhibited other phenotypes consistent with a replication defect, including slow growth and a significant decrease in mean nuclear signal of EdU (Supplementary Fig. 7a–e)^13,40^. These defects could be improved by expression of WT RNASEH2A, but not catalytic dead (CD) RNASEH2A^D34A;D169A^ or ribonucleotide excision deficient (separation of function (SOF)) RNASEH2A^P40D;Y210A^, a mutant which is unable to process single ribonucleotides but still cleaves RNA-DNA hybrids (Fig. 5b, c, Supplementary Fig. 7f)^39,41,42^. Expression of RNase H1, another mammalian ribonuclease with nucleolytic activity against RNA-DNA hybrids, could not improve the growth and EdU incorporation defects observed in RNASEH2A KO cells (Fig. 5d, e, Supplementary Fig. 7g)^43^. These data indicate that RNase H2, but not RNase H1 promotes DNA replication in mammalian cells and that it is the removal of single ribonucleotides that is important for normal replication speed maintenance.

**Fig. 6:**
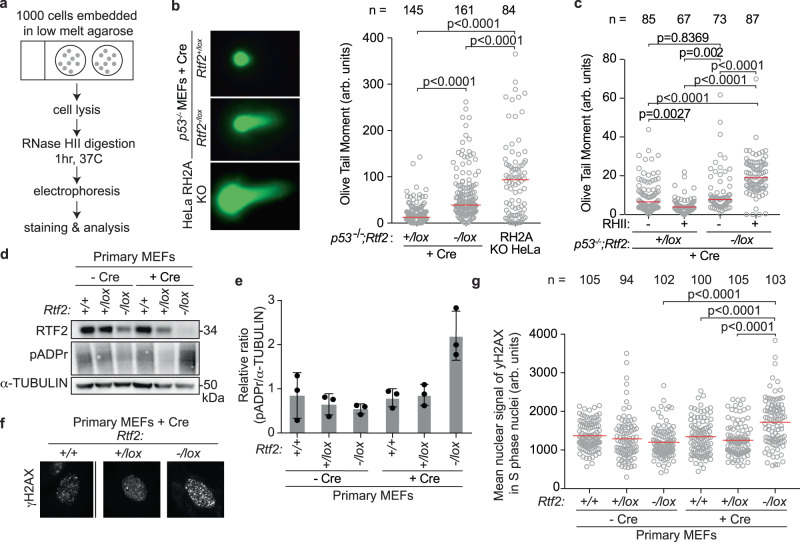
RTF2 recruits RNase H2 to the replication fork to facilitate removal of genomic ribonucleotides. **a** Schematic of neutral comet assay post RNase HII-digestion. **b** Left: Representative images of neutral comet assay post RNase HII-digestion with olive tail moment. Right: Quantification of olive tail moment in *p53*^*-/-*^ MEFs (72 hr after Cre), combined from 4 biological replicates. RNASEH2A KO HeLa cells serve as positive control. **c** Quantification of olive tail moment in *p53*^*-/-*^ MEFs (72 hr after Cre) with or without exogenous RNase HII digestion, combined from 2 biological replicates. **d** Representative immunoblot of whole cell lysates in primary MEFs 72 hr after Cre showing poly-ADP-ribosylation. **e** Average ratios from three biological replicates as in (**d**). **f** Representative immunofluorescent images of γH2AX staining in primary MEFs 72 hr after Cre. **g** Quantification of representative experiment of mean nuclear signal of γH2AX from (**f**). Experiments were conducted at least three times in biological replicates with consistent results for (**b**), (**d**), (**e**), (**f**), (**g**). Experiment was conducted twice in biological replicates for (**c**). Each dot represents one cell for (**b**), (**c**), (**g**). Mean for each sample shown with red line for (**b**), (**c**), (**g**). Experiments were blinded prior to analysis for (**b**), (**c**), (**g**). Error bars represent standard deviation in (**e**). Significance evaluated by Kruskal-Wallis ANOVA with a Dunn’s post-test. RH2A = RNASEH2A. Source data are provided as a Source Data file.

Depletion of RNASEH2A in RTF2-deficient cells did not further diminish the mean nuclear signal of EdU (Fig. 5f, g). Furthermore, under those conditions, expression of an RTF2 cDNA could not rescue the replication defect, suggesting that RNase H2 and RTF2 function in the same pathway during DNA replication (Fig. 5f, g, Supplementary Fig. 7h). Since RNase H2 removes ribonucleotides embedded in the newly replicated DNA and consequently *p53*^*-/-*^;*Rnaseh2b*^*-/-*^ MEFs harbor high levels of ribonucleotides in their genome, we asked whether cells without RTF2 show a similar increase in genomic ribonucleotides^13^. Using a neutral comet assay incorporating RNase HII digestion (Fig. 6a), we observed an increased load of genome-embedded ribonucleotides in RTF2-deficient cells as compared to RTF2-competent cells (Fig. 6b, c). Alongside increased genomic ribonucleotides, we observed poly-ADP-ribosylation and S phase-specific γH2AX phosphorylation in RTF2-deficient cells, two indicators of replication-dependent DNA damage following inappropriate TOP1-mediated ribonucleotide processing (Fig. 6d–g)^13,40,44–47^.

Taken together, our experiments show that failure to localize RTF2 and RNase H2 to the replisome leads to slow replication fork speeds and accumulation of genome-embedded ribonucleotides. These data support a model whereby RTF2 localizes RNase H2 to traveling replication forks to facilitate removal of genome-embedded single ribonucleotides incorporated during replication.

### Regulation of RTF2, RNase H2, and PRIM1 coordinate the response to replication stress and replication fork restart

Proper RTF2 levels at the replisome are necessary for the correct cellular response to replication stress^4^. Cells with an overabundance of replisome-associated RTF2 under conditions of DDI1/2-depletion exhibit increased sensitivity, accumulation of ssDNA, chromosomal breakage, and inefficient replication restart in response to treatment with replication stress-inducing agents^4^. Given that we showed above that RTF2 localizes RNase H2 to the replication fork to promote unperturbed DNA replication, we examined whether RTF2 and RNase H2 function together in the response to replication stress.

**Fig. 7:**
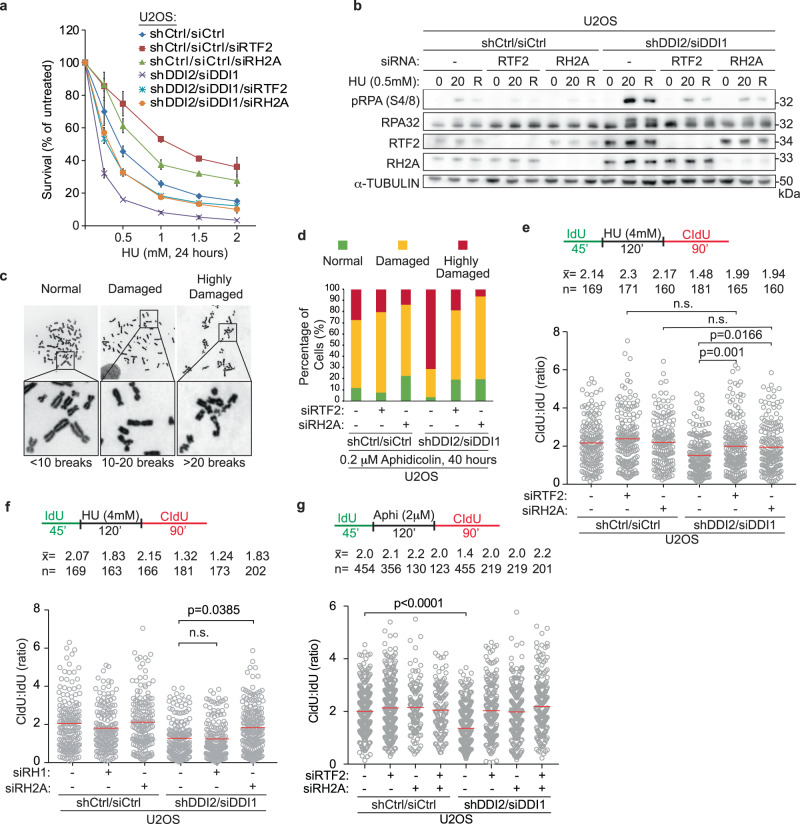
RTF2 and RNase H2 coordinate proper response to replication stress. **a** Representative cellular survival in HU-treated U2OS cells transduced or transfected with indicated RNAi reagents. **b** Representative immunoblot in U2OS cells transduced or transfected with indicated RNAi reagents and treated with HU (0 = untreated, 20 = 20 hr treatment, R = 20 hr treatment followed by 8 hr release). **c** Representative DNA metaphase spreads from U2OS cells after replication in the presence of low-dose aphidicolin for 40 hr. Metaphase spreads were categorized into three classes of breakage severity: normal ( < 10 breaks), damaged (10-20 breaks), and highly damaged/uncountable ( > 20 breaks). **d** Quantification of chromosome damage in a representative experiment of aphidicolin-treated U2OS cells transduced or transfected with indicated RNAi reagents. Quantification of representative experiments of CldU:IdU tract length ratio in DNA combing fork restart assay with fork stalling by HU (**e**, **f**) and aphidicolin (**g**). PBS washes are indicated by a black vertical line in all schematics. Experiment was conducted three times in biological replicates with technical triplicates for (**a**). Error bars represent standard deviation for (**a**). Experiments were conducted at least three times in biological replicates with consistent results for (**b**)–(**g**). Mean is shown with red line for (**e**)–(**g**). Experiments were blinded prior to analysis for (**d**)–(**g**). Average CldU:IdU ratios are listed above each sample for (**e**)–(**g**). Outliers were removed with ROUT (1%) for (**e**)–(**g**). Significance evaluated by Kruskal-Wallis ANOVA with a Dunn’s post-test. Ctrl Control, RH2A RNASEH2A, RH1 RNASEH1. Source data are provided as a Source Data file.

DDI1/2-depleted cells exhibit increased sensitivity to replication stress-inducing agents, including hydroxyurea (HU), aphidicolin, mitomycin C, and gemcitabine, that can be rescued by RTF2 depletion^4^. Like RTF2 depletion, knockdown of RNASEH2A rescued the sensitivity of DDI1/2-depleted cells to HU (Fig. 7a). Even in control cells, depletion of RTF2 or RNASHE2A reduced sensitivity to increasing doses of HU (Fig. 7a), suggesting that the overall level of RTF2 and RNASEH2A at the replisome may dictate the response to local replication stress. Depletion of RNASEH2A, like depletion of RTF2, reversed other phenotypes seen in cells without DDI1/2, including elevated p-RPA S4/8 signaling during low dose HU treatment (Fig. 7b, Supplementary Fig. 8a) and genome instability as measured by presence of chromosomal abnormalities, which are a direct outcome of an inappropriate replication stress response (Fig. 7c, d, Supplementary Fig. 8b). These data suggest that DDI1/2-dependent removal of RTF2 and RNase H2 from stressed replication forks is important for the proper replication stress response.

A striking phenotype associated with DDI1/2-depletion is a decreased percentage of restarted replication forks and a decreased efficiency of fork restart after release from HU treatment^4^. To test the replication restart defects under these conditions, we used a modified DNA combing assay in which the initial 45 minute pulse of IdU is followed by complete replication block with 4 mM HU (Supplementary Fig. 8c). Following removal of HU, the length of the second nascent DNA tract (CldU) is compared to the length of first tract (IdU) as a proxy for replication restart efficiency (Fig. 7e). The fork restart defect observed upon treatment with HU in DDI1/2-depleted cells was rescued to the same extent by depletion of RTF2 or RNASEH2A (Fig. 7e), while RTF2- or RNASEH2-depleted cells had unperturbed ratios of CldU:IdU when untreated (Supplementary Fig. 8d). This effect was specific to RNase H2, as depletion of RNase H1 did not rescue the fork restart defect in DDI1/2-depleted cells after HU treatment (Fig. 7f, Supplementary Fig. 8e, f). These experiments suggest that both RTF2 and RNase H2 must be removed from replication forks to promote efficient replication restart following fork stalling.

To show that this restart defect was not specific to fork stalling by HU, we attempted to repeat this assay using camptothecin (CPT), gemcitabine, and aphidicolin. Replication could not restart within a short time after complete stalling induced by CPT or gemcitabine (Supplementary Fig. 8g, h). This is most likely due to an inability to directly restart lesions, which necessitate nucleolytic processing and homology-directed repair, induced by these agents^48^. However, replisomes completely stalled by 2 μM aphidicolin treatment were able to restart in a timely manner once aphidicolin was removed, and fork restart depended on the depletion of RTF2 and RNASEH2A under those conditions (Fig. 7f and Supplementary Fig. 7i). These data show that replication restart is sensitive to levels of RTF2 and RNase H2 not only in response to HU, but also aphidicolin.

These findings are consistent with the DDI/RTF2 pathway regulating the RNase H2 complex at the replication fork to allow proper recovery from transient DNA replication stress. If persistent, RNase H2 seems to interfere with an activity necessary for efficient replication restart of stalled forks. As RNase H2 processes RNA-DNA hybrids, we hypothesized that persistent RNase H2 endonucleolytically processes an RNA primer necessary to resume replication. To test this hypothesis, we examined the contribution of the two AEP primases, PRIMPOL and PRIM1 on the efficiency of replication restart after transient replication stalling^49^.

**Fig. 8:**
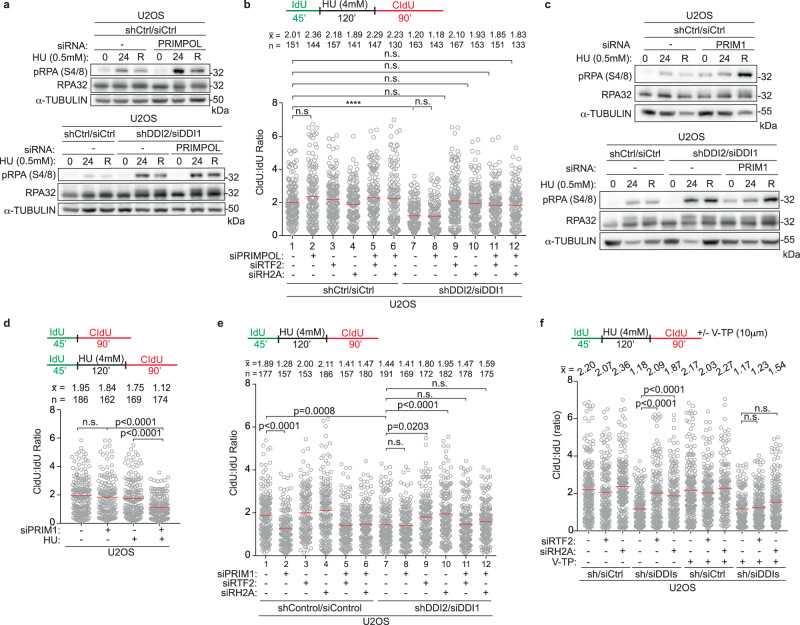
Catalytic activity of primase PRIM1, not PRIMPOL, is required for efficient replication restart after stress. **a** Representative immunoblot in indicated U2OS cells treated with HU (0 = untreated, 24 = 24 hr treatment, R = 24 hr treatment followed by 8 hr release). α-tubulin represents loading control. **b** Top: Labeling schematic. PBS washes are indicated by a black vertical line in all schematics. Bottom: Ratio of CldU:IdU tract lengths in indicated U2OS cells. **c** Representative immunoblot in U2OS cells transfected with siRNA and treated with HU as indicated (0 = untreated, 24 = 24 hr treatment, R = 24 hr treatment followed by 8 hr release). α-tubulin represents loading control. **d–f** Top: Schematic of labeling scheme. PBS washes are indicated by a black vertical line in all schematics. In (**f**), V-TP, a potent PRIM1 inhibitor was added as indicated. Bottom: Quantification of representative experiment of CldU:IdU tract length ratio in U2OS cells after indicated perturbations, including transfuction or transfection with indicated RNAi reagents. Experiments conducted at least three times in biological replicates with consistent results for (**a**)–(**f**). Mean is shown with red line for (**b**), (**d**), (**e**), (**f**). Experiments were blinded prior to analysis for (**b**), (**d**), (**e**), (**f**). Average CldU:IdU ratios are listed above each sample for (**b**), (**d**), (**e**), (**f**). Outliers removed with ROUT (1%) for (**b**), (**d**), (**e**), (**f**). Significance evaluated by Kruskal-Wallis ANOVA with a Dunn’s post-test. RH2A = RNASEH2A, Ctrl = Control. Source data are provided as a Source Data file.

PRIMPOL depletion resulted in an increase in p-RPA S4/8 signal after HU treatment regardless of presence or absence of DDI1/2, a finding consistent with PRIMPOL’s function infilling in gaps behind the replication fork (Fig. 8a)^50^. Despite its reported role in fork restart after UV, depletion of PRIMPOL had no effect on the efficiency of replication fork restart after release from HU (Fig. 8b, in b, compare lanes 1 and 2, Supplementary Fig. 9a–c)^28,30,32^. Moreover, the rescue of replication restart under conditions of DDI1/2 and RTF2 (or RNASEH2A) co-depletion was not diminished upon PRIMPOL depletion (Fig. 8b, in b, compare lanes 9, 10, 11, and 12). These data are consistent with a role for PRIMPOL in filling in gaps behind the replication fork, a pathway that does not seem to involve DDI1/2, RTF2, or RNase H2.

We next examined the role of PRIM1, the catalytic primase subunit of the DNA polymerase α-primase complex, during replication fork restart. Knockdown of PRIM1 induced p-RPA S4/8 in untreated conditions that was exacerbated with HU treatment (Fig. 8c). Although cells depleted of PRIM1 maintained an unperturbed ratio of CldU:IdU under untreated conditions, they exhibited decreased fork restart efficiency after treatment with HU indicating that replication restart is reliant on PRIM1 activity. (Fig. 8d, Supplementary Fig. 9d–f) This dependency was seen regardless of RTF2 or RNASEH2A status (Fig. 8e, Supplementary Fig. 9d–f). In a parallel experiment, we found that low levels of PRIM1 inhibitor vidarabine-TP (V-TP, 10 μM) did not significantly perturb normal replication but resulted in inefficient restart after HU treatment, an effect that could not be overcome by depletion of RTF2 or RNASEH2A (Fig. 8f, Supplementary Fig. 9g, h)^51^. These data suggest that PRIM1 catalytic activity is essential for efficient replication restart in mammalian cells.

**Fig. 9:**
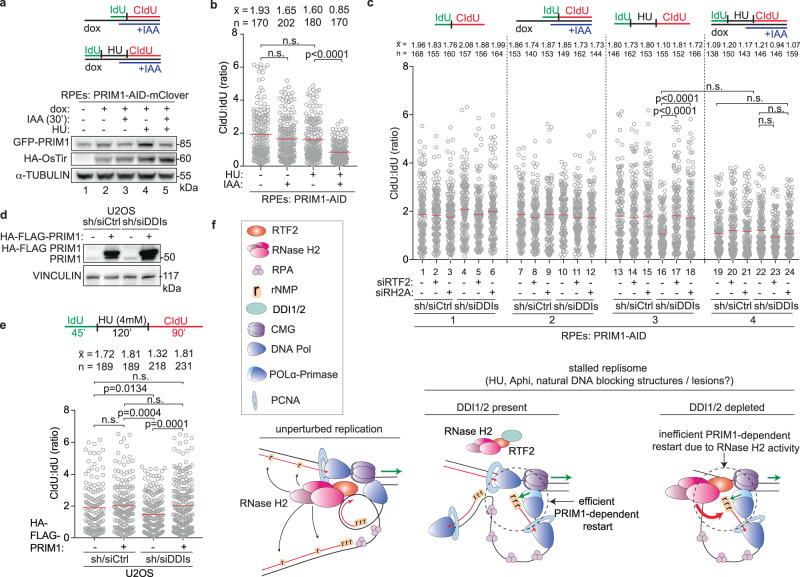
Replication restart is exquisitely sensitive to cellular levels of PRIM1. **a** Top: Schematic of labeling scheme. Dox was used to induce expression of HA-OsTir enzyme for targeted PRIM1-AID-mClover degradation. PBS washes are indicated by a black vertical line in all schematics. Bottom: Representative immunoblot of PRIM1 levels in endogenously edited PRIM1-AID-mClover RPE cells following indicated treatments. Lane 3 normalized expression of GFP-PRIM1 is 16% less than the normalized expression in lane 2. Lane 5 normalized expression of GFP-PRIM1 is 24% less than the normalized expression in lane 4. **b** Quantification of representative experiment of CldU:IdU tract length ratio in cells from experiment in (**a**). **c** Top: Schematic of labeling scheme. Quantification of representative experiment of CldU:IdU tract length ratio. **d** Immunoblot of HA-FLAG-PRIM1 expression in control and DDI-depleted U2OS cells. **e** Top: Schematic of labeling scheme. Bottom: Quantification of representative experiment of CldU:IdU tract length ratio in cells from experiment in (**d**). **f** Model of RTF2’s function in localizing RNase H2 to the replisome during unpurturbed replication to allow for single ribonucleotide removal (left). Model of RTF2 and RNase H2 affecting PRIM1-dependent restart following replication stress (right). We speculate that in the DDI1/2-competent cells, RTF2 is removed from stalled replication forks through the activity of the DDI1/2 proteasome shuttle proteins, resulting secondarily in loss of RNase H2 and efficient restart that is dependent on the levels and catalytic activity of PRIM1. In DDI1/2-depleted cells, RTF2-RNase H2 are retained at stalled replication forks, opposing RNA primer deposition by PRIM1 and slowing replication restart after stalling. Experiments conducted at least three times in biological replicates with consistent results for (**a**)–(**e**). Mean is shown with red line for (**b**), (**c**), (**e**). Experiments were blinded prior to analysis for (**b**), (**c**), (**e**). Average CldU:IdU ratios are listed above each sample for (**b**), (**c**), (**e**). Outliers removed with ROUT (1%) for (**b**), (**c**), (**e**). Significance evaluated by Kruskal-Wallis ANOVA with a Dunn’s post-test. RH2A = RNASEH2A, Ctrl = Control. IAA = auxin. Source data are provided as a Source Data file.

As PRIM1’s essential role in DNA replication could confound the results obtained with a 72-hr depletion of PRIM1, an auxin-inducible degron (AID) system was employed to partially degrade PRIM1 within 30 minutes (Fig. 9a)^52^. An acute 24% reduction in PRIM1 expression resulted in a significant decrease in replication restart efficiency (Fig. 9b). The addition of auxin in the untreated condition (no HU) did not significantly change the length of replication tracks in the presence of the first thymidine analog, IdU (Supplementary Fig. 10a). These data indicate that replication restart is exquisitely sensitive to even small changes in PRIM1 levels.

To understand the genetic interaction of PRIM1 with the DDI-RTF2 pathway, DDI1/2 were depleted in RPE PRIM1-AID-mClover cells. No further reduction in replication restart efficiency was observed with combined reduction of PRIM1 and DDI1/2 levels (Fig. 9c). However, depletion of PRIM1 prevented the rescue of replication restart seen with the depletion of RTF2 or RNASEH2A in the DDI1/2-depleted setting (Fig. 9c, compare lanes 17,18 to 23,24), suggesting that PRIM1 and RTF2/RNASEH2A function in the same pathway. Depletion of DDI1/2 did not affect the levels of PRIM1 at either progressing or stalled replication forks (Supplementary Fig. 10b). However, DDI1/2-depleted cells overexpressing HA-PRIM1 were proficient in replication restart following stalling by HU, despite retained levels of RTF2-RNase H2 (Fig. 9d, e, Supplementary Fig. 10e).

Based on the data presented here, we propose that PRIM1 activity at the stalled replication fork allows for repriming and direct replication restart, enabling the resumption of replication without replication fork collapse. While biochemical reconstitution studies in bacteria and yeast have provided evidence for DNA polymerase α-primase-dependent re-priming on the lagging strand, we provide the first evidence of PRIM1-dependent replication fork restart in mammalian cells^27,53–55^. Our data show that RTF2, which is dynamically regulated at replication forks in the setting of DNA damage, facilitates efficient PRIM1-dependent restart of stalled forks by removing RNase H2 (Fig. 9f).

## Discussion

The identification of DDI1/2 and RTF2 as coordinators of RNase H2 localization to the replication fork implicates the DDI/RTF2/RNase H2 axis as central in regulating unperturbed DNA replication and the replication stress responses. Our data show that RNase H2 must be tightly controled during the S phase to ensure a proper balance between replication repriming and ribonucleotide excision. Genomic ribonucleotide incorporation in S phase may result from inappropriate incorporation by the replicative polymerases, inefficient Okazaki fragment maturation, or replication-associated DNA repair^56–58^. We show that the decreased speed seen in cells lacking RTF2 parallels the lack of RNase H2 localization to the replication fork. Based on these data, we propose that RNase H2 is tethered to the replisome through RTF2, where it removes single ribonucleotides incorporated by DNA polymerases (Fig. 9f, unperturbed replication).

We find that localization of RNase H2 to replication forks is RTF2-dependent and PCNA-independent. These findings were surprising at first, given previous reports of PCNA-dependent localization of RNase H2 to sites of replication through RNASEH2B’s PIP box^11^. However, they are consistent with prior work identifying PCNA-independent localization of RNase H2 to replication forks through RNASEH2A^11,12^. Further studies will be necessary to identify conditions when the PCNA-RNase H2 interaction is necessary for cellular function.

Many pathways exist to rescue stalled replisomes^1,3^. As shown here, PRIM1-dependent restart is an important mechanism for continuing replication after stalling. We find it interesting that small changes in levels of PRIM1, the RNA catalytic subunit of the α-primase, have large effects during replication restart, indicating that the restart process is very sensitive to both the levels of PRIM1 and its activity. This could be due to the weak affinity of PRIM1 for the substrate as recently described, or may have a yet-to-be defined structural basis^59^.

Our data are consistent with the idea that the action of PRIM1, which creates RNA primers for replication restart of temporarily stalled replisomes, can be counteracted by inappropriately retained RNase H2. We propose that to permit an efficient restart, RNase H2 is removed from the stalled fork. Since the localization of RNase H2 is dependent on RTF2, the removal of RTF2 from the replication fork would also remove RNase H2, preventing the destruction of RNA primers and allowing efficient direct restart (Fig. 9f, stalled replisome).

This model raises a question of how the RNase H2 present at the replisome is prevented from interfering with the deposition of RNA primers during normal replication. Structural differences may exist between the stalled and progressing replisomes that allow access of RNase H2 to the primers at the latter. We suspect that other proteins and regulatory networks may also prevent close association of PRIM1 and RNase H2 while replication is ongoing. Future work will be needed to biochemically and structurally define the differences in RNase H2 localization at stalled and progressing forks and to determine how the DDI1/2-RTF2 axis mediates these dynamic changes.

This discovery of RTF2’s role in regulating replication speed and the response to replication stress suggests it is a regulatory hub at the replisome. Fork stalling agents, like aphidicolin and HU, mimic the replication stalling that the replisome faces during duplication of difficult-to-replicate regions like common fragile sites and repetitive sequences. Uncovering the mechanisms of RTF2’s regulation will lend insight into the coordination of replication through vast areas of the genome undergoing transient replication stress.

Both RNase H2 and PRIM1 have been implicated in human disease. *RNASEH2* pathogenic variants have been identified in the inflammatory disease AGS and cancer^60^. A pathogenic variant in *PRIM1* was reported to cause a primordial dwarfism syndrome^61^. Understanding the basic mechanism underlying the balance in their activities during DNA replication will facilitate understanding of diverse human diseases. Further, the DDI/RTF2/RNase H2/PRIM1 axis contains druggable targets to sensitize cells to replication stress-inducing agents currently entering the clinic.

## Methods

### Availability of materials

Materials generated by our lab will be shared with proper material transfer agreements in place upon request from the corresponding author. All commercial materials are indicated in Supplementary Table 1.

### Generation of Rtf2 mouse strains, maintenance and genotyping

mESCs containing the *Rtf2* gene targeting construct (*Rtf2*^*tm1a(KOMP)Wtsi*^) were obtained from the UCDavis KOMP Repository. The Rockefeller transgenics facility injected targeted mESCs into C57BL/6 J blastocysts to generate chimeras. *Rtf2*^*+/stop*^ mice were mated with mice expressing Flp^62^ to generate *Rtf2*^*+/lox*^ mice. *Rtf2*^*+/lox*^ mice were then mated with mice expressing Cre recombinase from a ubiquitous EIIa promoter^63^. All animals were handled according to the Rockefeller University Institutional Animal Care and Use Committee protocols. See Supplementary Table 1 for summary of mouse strains.

Mouse tail tips were obtained from pups at day 21. Tails were lysed in DirectPCR Lysis Reagent (Mouse Tail) supplemented with 0.2 mg/mL proteinase K according to manufacturer’s protocol. 0.2-1.0 μL of lysate was used for 20 μL PCR reaction. GoTaq® DNA polymerase master mix was used for PCRs with appropriate primers. Sanger sequencing was performed at GeneWiz. See Supplementary table 1 for mESC long range genotyping primers and for mouse genotyping primers.

### Cell culture

Mouse embryonic fibroblasts (MEFs) were isolated on embryonic day 13.5 from crosses of *Rtf2*^*+/lox*^ and *Rtf2*^*+lox*^ mice (*Rtf2*^*+/+*^*, Rtf2*^*+/lox*^, and *Rtf2*^*lox/lox*^ MEFs) and crosses of *Rtf2*^*+/lox*^ and *Rtf2*^*+/-*^ mice (*Rtf2*^*+/+*^*, Rtf2*^*+/lox*^*, Rtf2*^*+/-*^, and *Rtf2*^*-/lox*^ MEFs). MEFs were expanded to obtain cells for early passage primary cell stocks and to immortalize cells with a retrovirus expressing SV40-LT^64^. Experiments were conducted with littermate MEFs. Multiple MEF lines from individual mothers were isolated and used in replicate experiments. *Rtf2*^*-/-*^ clonal cell lines were also generated from SV40-LT immortalized *Rtf2*^*-/lox*^ MEFs after infection with pMMP Hit & Run Cre recombinase^65^. For conditional *Rtf2* cell lines, experiments were performed 72–120 hr after transduction with pMMP Hit & Run Cre recombinase as indicated^65^. *p53*^*-/-*^*; Rtf2*^*+/lox*^ and *Rtf2*^*-/lox*^ MEF lines were generated using CRISPR gene editing at the p53 locus using the pX459 plasmid^66^. See Supplementary table 1 for list of mutagenesis primers and plasmids.

Primary MEFs and BJ foreskin fibroblasts (transformed by HPV16 E6E7 expression and/or immortalized by expression of catalytic subunit of human telomerase (hTERT)) were maintained in DMEM, supplemented with 15% (v/v) fetal bovine serum (FBS), 1X MEM non-essential amino acids, 2 mM L-alanyl-L-glutamine dipeptide, and 100 U/mL penicillin-streptomycin. MEFs virally transformed with SV40-LT, HEK293T cells, U2OS cells (as described in Kottemann *et al*.) and HeLa cells (Jackson lab) were maintained in DMEM supplemented with 10% FBS and the additional supplements described above. RPE *p53*^*-/-*^*, pRb*^*-/-*^, PRIM1-AID-mClover cells (de Lange lab) were maintained in DMEM/F12 supplemented with 10% FBS and the additional supplements described above. HCT-116 *p53*^*-/-*^ (Durocher and Jackson lab) cells were maintained in McCoy’s 5 A media supplemented with 10% FBS and the additional supplements described above. Upon confluence, cells were dissociated with trypsin and a fraction of the cells were passaged into a new dish. Cells were cryopreserved in their respective media supplemented with 10% DMSO. Cell lines were validated by immunoblotting or qPCR, as shown in the Supplementary Figs. for each cell line used. See Supplementary table 1 for summary of cell types.

### Growth and sensitivity assays

For growth assays, 2 × 10^4^ − 3 × 10^4^ cells were plated in each well of a 6-well plate in triplicate. Wells were counted on subsequent days using a Z2 Coulter Counter Analyzer (Beckman Coulter). Population doublings were calculated using the following formula: 3.32 x [log(the number of cell harvested)–log(the initial number of cells plated)].

For sensitivity assays, 2.5 × 10^4^ – 4.5 × 10^4^ cells were plated in each well of a 6-well plate in triplicate. The following day drugs were added at indicated concentrations. After 5-6 days in culture, cells were passaged once at appropriate ratios. Cells were counted when untreated wells reached near confluence around 7-9 days. The cell numbers at each dose of drug were divided by the cell number in the untreated sample to calculate the percent survival.

### RNA preparation, reverse transcription, and real-time quantitative PCR

Total messenger RNA was extracted from cells using RNeasy Plus Mini Kit. RNA was reverse transcribed using the SuperScript^TM^ III First-Strand Synthesis System. The relative transcript levels of genes of interest were determined by RT-qPCR using Platinum^TM^ SYBR^TM^ Green SuperMix-UDG. All kits were used according to manufacturer’s protocol. Reactions were run and analyzed on Applied Biosystems™ QuantStudio™ 12 K Flex system. See supplementary information for RT-qPCR primers.

### Plasmid generation and mutagenesis

cDNA from MEFs or BJ cells was PCR amplified with primers containing attB sites. attB-PCR products were cloned using the Gateway® system into pDONR223^67^ with BP Clonase II Enzyme Mix. Site directed mutagenesis of pDONR223 derivatives was performed with Agilent QuikChange or Agilent QuikChange kits according to manufacturer’s protocol. pDONR223 derivatives with inserted cDNAs (pENTR vectors) were cloned into destination vectors with LR clonase II Enzyme Mix^42,68^. Reactions were transformed into chemically competent DH5-α or Stlb3 *E. coli* cells and plated onto Luria-Bertani (LB)/agar plates with the appropriate bacterial selection (kanamycin (50 μg/ml), spectinomycin (50 μg/mL), chloramphenicol (25 μg/ml), or ampicillin (100 μg/ml)). Clones were mini-prepped and sequences were confirmed with Sanger sequencing (GeneWiz). Appropriate clones were maxi-prepped and ethanol precipitated for sterile tissue culture use. See supplementary information for Gateway primers, mutagenesis primers and a list of plasmids.

pEGFP‐RNASEH2B was a gift from Andrew Jackson & Martin Reijns (Addgene plasmid # 108697; http://n2t.net/addgene:108697; RRID:Addgene_108697). pMSCVpuro-DEST was a gift from Andrew Jackson & Martin Reijns (Addgene plasmid # 119745; http://n2t.net/addgene:119745; RRID:Addgene_119745). ppyCAG_RNaseH1_WT was a gift from Xiang-Dong Fu (Addgene plasmid # 111906; http://n2t.net/addgene:111906; RRID:Addgene_111906). ppyCAG_RNaseH1_D210N was a gift from Xiang-Dong Fu (Addgene plasmid # 111904; http://n2t.net/addgene:111904; RRID:Addgene_111904). pcDNA5-FRT-TO-EGFP-AID was a gift from Andrew Holland (Addgene plasmid # 80075; http://n2t.net/addgene:80075; RRID:Addgene_80075). MLM3636 was a gift from Keith Joung (Addgene plasmid # 43860; http://n2t.net/addgene:43860; RRID:Addgene_43860). pX330-U6-Chimeric_BB-CBh-hSpCas9 was a gift from Feng Zhang (Addgene plasmid # 42230; http://n2t.net/addgene:42230; RRID:Addgene_42230). pGEX6P1‐hsRNASEH2BCA was a gift from Andrew Jackson & Martin Reijns (Addgene plasmid # 108692; http://n2t.net/addgene:108692; RRID:Addgene_108692). pMD2.G was a gift from Didier Trono (Addgene plasmid # 12259; http://n2t.net/addgene:12259; RRID:Addgene_12259). psPAX2 was a gift from Didier Trono (Addgene plasmid # 12260; http://n2t.net/addgene:12260; RRID:Addgene_12260). pLVpuro-CMV-N-EGFP was a gift from Robin Ketteler (Addgene plasmid # 122848; http://n2t.net/addgene:122848; RRID:Addgene_122848). pSpCas9(BB)−2A-Puro (PX459) V2.0 was a gift from Feng Zhang (Addgene plasmid # 62988; http://n2t.net/addgene:62988; RRID:Addgene_62988). pMSCV_PM_shRNA_Control_puro was a gift from Steve Elledge^69^. pMMP Hit & Run Cre was a gift from David Livingston^65^. pWZL Cre-hygro was a gift from Titia de Lange^52^. PSKA002 HIS14-SUMO-MCS Expression Vector and PSKA008 HIS14-GFP-MCS-Expression Vector were gifted from Sebastian Klinge.

### Transductions

cDNAs were delivered by retroviral or lentiviral transduction after packaging in HEK293T cells. 5 × 10^6^ were plated the evening before transfection. DNA and viral packaging vectors were transfected into cells with TransIT-293 transfection reagent according to the manufacturer’s protocol. The media was changed the next day and after 24 hr, viral supernatants were harvested and filtered (0.45 μM). Harvests were repeated every 12 hr for 2 days. Target cells were infected with viral supernatants supplemented with 4 μg/mL polybrene. Stably expressing cells were selected with the appropriate agent ((puromycin (0.5-2 μg/ml), hygromycin (100 μg/ml), blasticidin (600 μg/ml), neomycin (600 μg/ml)). See supplementary information for the list of plasmids.

### Generation of GFP-AID-RTF2 endogenously tagged 293Ts

The pUC19-EGFP-AID-hRTF2 donor construct was generated with classical and In-Fusion® HD cloning techniques. hRTF2 was amplified with BamHI and NotI digestion sites and ligated into the pcDNA5-FRT-TO-EGFP-AID backbone. Using this as a PCR template, a GFP-AID-hRTF2 construct was amplified with primers compatible for In-Fusion. 5’ and 3’ UTR regions of RTF2 were also amplified with primers compatible with In-Fusion. Subsequently purified PCR products from these reactions were cloned into a HindIII- and EcoRI-digested pUC19 backbone with In-Fusion®, generating a donor construct containing homolog arms in the 5’ UTR and 3’ UTR and a cDNA for GFP-AID-RTF2. HEK293Ts were transfected using TransIT-293 with this donor construct, a pX330 guide RNA-Cas9 construct, and a single guide RNA construct in a 1:1:1 ratio^70^. Cells were grown for 48-72 hr before sorting for GFP+ populations. GFP+ populations were sub-cloned for single cell clones. These clones were verified for integration of the GFP-AID-RTF2 cDNA into the endogenous *Rtf2* locus. See supplementary information for the list of CRISPR cloning primers.

### Immunoblotting

If cells were counted upon collection, an equal number of cells were lysed by resuspension in an equal volume (100 μL per 1 ×10^6^ cells) of hot Laemmli buffer (Bio-Rad). If cells could not be counted upon collection, whole cell lysates were prepared by lysing cells in Laemmli buffer (4% SDS, 20% glycerol, 0.125 M Tris-HCl (pH=6.8)), to determine protein concentration by Lowry protein assay prior to addition of 2-mercaptoethanol and bromophenol blue. Samples were either sonicated or passed through a tuberculin needle 10 times. Subsequently, samples were boiled for 5 minutes. Equal amounts of protein were separated by sodium dodecyl sulfate polyacrylamide gel electrophoresis (SDS-PAGE) on precast 4–12% Bis-Tris gels. Membranes were blocked for 1 hr in 5% milk in TBST (10 mM Tris-HCl (pH=7.5), 150 mM NaCl, 0.1% Tween 20) and incubated in primary antibodies for 2 hr at room temperature or overnight at 4 °C. Membranes were sufficiently washed in TBST (3 ×10 minutes) before being incubated with horseradish peroxidase (HRP)-conjugated secondary antibodies for 1 hr at room temperature, sufficiently washed again and detected by enhanced chemiluminescence. Membranes were visualized with either ImageQuantLAS 4000 or Azure c300 imaging systems. Antibodies used: Mouse monoclonal anti-α-tubulin (clone DM1A), WB:1:5000 (MilliporeSigma, Cat# T9026, RRID:AB_477593); Mouse monoclonal anti-Poly (ADP-Ribose) Polymer antibody [10H], WB:1:100 (Abcam, Cat # ab14459, RRID:AB_301239); Mouse monoclonal anti-PCNA (PC10), WB: 1:1000 (Santa Cruz, Cat# sc-56, RRID:AB_628110); Mouse monoclonal anti-RNASEH2A, WB:1:500 (Santa Cruz, Cat# sc-515475); Mouse monoclonal anti-RTF2 (clone OTI1E8), WB: 1:1000 (LS Bio, Cat# LS-C340588); Mouse Monoclonal anti-vinculin, Unconjugated, Clone hVIN-1, WB: 1:5000 (MilliporeSigma, Cat# V9131, RRID:AB_477629); Rabbit monoclonal anti-MCM7 (D10A11) XP, WB: 1:1000 (Cell Signaling, Cat# 3735 S, RRID:AB_2142705); Rabbit polyclonal anti-c20orf43 (RTF2), WB: 1:500 (Novus, Cat# NBP2-30645); Rabbit polyclonal anti-GFP, 1:1000 (Abcam, Cat# ab290 RRIDL AB_303395); Rabbit polyclonal anti-RNASEH2A, 1:1000 (Abcam, Cat# ab83943, RRID:AB_1861175); Rabbit polyclonal anti-RNASEH2C (AbClonal, Cat# A13884, RRID:AB_2760737); Rabbit polyclonal anti-phospho-RPA32 (S4/8), WB:1:1000 (Bethyl, Cat# A300-245A, RRID:AB_210547); Rabbit polyclonal anti-RPA32, WB 1:2000 (Bethyl, Cat# A300-244A, RRID:AB_185548); Rabbit polyclonal anti-PRIM1 (Proteintech, Cat # 10773-1-AP, RRID:AB_2237549).

### siRNA transfections

Cells were transfected with pools of 3 siRNAs against DDI1 as previously published^4^. For RNASEH1, PRIM1 and PRIMPOL, pools of 3 siRNAs were used. For RNASEH2 and RTF2 depletion, a single siRNA was used. Cells were transfected using Lipofectamine™ RNAiMAX Transfection Reagent according to manufacturer’s instructions with the exception that siRNA-lipid complexes were added to the well and then cells were seeded on top of complexes. Knockdown was measured by RT-qPCR or western blot. See supplementary information for RNAi sequences.

### Immunofluorescence and nascent proximity ligation assay

#### EdU staining

Cells were pulsed with 10 μM EdU for 1 hr, then washed in 1x PBS and fixed in 3.7% (v/v) formaldehyde in PBS at RT for 10 minutes. Cells were washed and permeabilized in 0.5% (v/v) Triton X-100 in PBS. Cells were blocked in either 5% (v/v) FBS in PBS or 3% BSA (w/v) in PBS at RT for 30 minutes. Cells were stained with Click-iT™ EdU Alexa Fluor™ 488 Imaging Kit (Invitrogen, C10337) according to manufacturer’s protocol. Cells were washed and embedded on glass slides with DAPI Fluoromount-G (SouthernBiotech). Mean nuclear signal is mean gray value calculated with image analysis using FIJI.

#### γH2AX staining

Cells were fixed and permeabilized as above. Slides were incubated with primary antibodies in blocking buffer for 2 hr at room temperature or overnight at 4 °C. Cells were washed with blocking buffer and then incubated with secondary antibody. Cells were washed and embedded on glass slides as above. Mean nuclear signal is mean gray value calculated with image analysis using FIJI. Antibodies used: Mouse monoclonal anti-γH2AX Ser139 (clone JBW301), IF 1:2000 (MilliporeSigma, Cat# 05-636, RRID:AB_309864).

#### Nascent PLA staining

This assay was optimized to detect the amount of protein at the replication fork by labeling with short pulses of 10 μM EdU. The duration of EdU pulse was determined by DNA combing experiments to label equal amounts of nascent DNA across different conditions. Cells were washed with PBS, permeabilized with 0.5% Triton X-100 in PBS, fixed with 3% formaldehyde/2% sucrose in PBS and blocked with 3% BSA in PBS. EdU was then biotin-clicked and coverslips were incubated with either mouse anti-biotin and rabbit anti-RNASEH2A, or with rabbit anti-biotin and mouse anti-GFP overnight. The next day, antibody-coupled sense and anti-sense probes were used to detect the light chains of rabbit and mouse IgG, respectively, followed by the PLA reaction (DuoLink) according to the manufacturer’s protocol. If the probes are within 30-40 nM of each other, they are ligated and amplified to produce a fluorescent signal. Where indicated, cells were co-stained with PCNA prior mounting on slides. Slides were imaged (Inverted Olympus IX-71 DeltaVision Image Restoration Microscope (Applied Precision) or Axio Observer.A1 fluorescence microscope (Carl Zeiss), equipped with a Plan- Apochromat 63× NA-1.4 oil objective, the AxioCam CCD camera, and the AxioVision Rel Version 4.7 software. Foci were counted using Cell Profiler software using either DAPI or PCNA co-stain to detect nuclei. Antibodies used: Mouse monoclonal anti-biotin, IF: 1:2000 (Jackson ImmunoResearch, Cat# 200-002-211, RRID:AB_2339006); Rabbit polyclonal anti-GFP, 1:1000 (Abcam, Cat# ab290 RRIDL AB_303395); Rabbit polyclonal anti-RNASEH2A, 1:1000 (Abcam, Cat# ab83943, RRID:AB_1861175).

### Cell cycle

Exponentially growing cells were labeled with 10 μM EdU for 1 hr prior to collection and fixation. Cell cycle preparation was performed with Click-iT™ EdU Alexa Fluor™ 647 Flow Cytometry Assay Kit per the manufacturer’s protocol. Total DNA content was stained with FxCycle™ Violet. Stained cells were analyzed on a BD Accuri^TM^ C6 or BD^TM^ LSR II cytometers. Data was analyzed with FlowJo software.

### RNA sequencing and analysis

Total messenger RNA was extracted using RNeasy Plus Mini Kit and DNase treated prior to submission to Rockefeller University’s genomic core for library preparation. Libraries for intron retention were prepared using rRNA depletion and run on NextSeq 500 High Output for 75 base pair paired end reads. Libraries for transcript analysis were prepared using standard Illumina sequencing primers and run on NextSeq 500 High Output for 75 base pair single reads. For intron retention analysis, raw reads were aligned to the mouse genome (mm10) with Strand NGS (version 2.1) software. Percentage of reads mapping to introns was from determined post-alignment statistics.

Sequence and transcript coordinates for mouse mm10 UCSC genome and gene models were retrieved from the Bioconductor Bsgenome.Mmusculus.UCSC.mm10 (version 1.4.0) and TxDb.Mmusculus.UCSC.mm10.knownGene (version 3.4.0) Bioconductor libraries respectively. Transcript expressions were calculated using the Salmon quantification software (version 0.8.2) and gene expression levels as TPMs and counts retrieved using Tximport (version 1.8.0). Normalization and rlog transformation of raw read counts in genes were performed using DESeq2 (version 1.20.0). For visualization in genome browsers, RNA-seq reads are aligned to the genome using Rsubread’s subjunc method (version 1.30.6) and exported as bigWigs normalized to reads per million using the rtracklayer package (version 1.40.6).

### Isolation of proteins on nascent DNA (iPOND)

Cells were pulsed with 10 μM EdU for differential times to yield similar labeled lengths. The duration of EdU pulse was determined by DNA combing experiments to label equal amounts of nascent DNA across different conditions. iPOND was performed as previously described^37^ with Dynabeads™ MyOne™ Streptavidin C1 (Invitrogen, 65001). Eluate was run only 1 cm into a 4%–12% Bis-Tris gel (Invitrogen) and submitted for mass spectrometry analysis at Rockefeller University’s Proteomics Core. For iPOND immunoblots, the eluate was run on 4%–12% Bis-Tris gels (Invitrogen) and immunoblotted as indicated.

### Co-immunoprecipitations

Endogenous co-immunoprecipitations were seeded the previous day at a concentration of 10-20 ×10^6^ cells per 15 cm. Cells were treated 1 hr prior to collection with 10 μM MG-132. Cells were then washed 1x with cold PBS, harvested by scraping, and spun at 500 x g for 5 min at 4 °C. Cells were subsequently washed 2x with cold PBS. Cells were lysed in 1 mL of cold lysis buffer (50 mM HEPES pH=7.5, 150 mM NaCl, 2 mM MgCl2, 0.1% Tween-20, 1x Phosphatase inhibitor cocktail II, 1x protease inhibitor (Roche, cOmplete EDTA-free, 11697498001), 2 mg/mL N-ethylmaleimide). Cells were sonicated (3x 10 A for 15 seconds) and treated with benzonase for 30 minutes. Lysates were centrifuged to remove debris. The cleared lysate was incubated with antibodies and Dynabeads™ (Invitrogen) or antibody-coupled Dynabeads™ M-270 Epoxy resin (Invitrogen). Normal IgG was used for antibody controls. Lysates were incubated at 4 °C for 2 hr on nutator and then washed with lysis buffer. Samples were heated for 10 minutes in 2 x LDS buffer to elute and run on 4-12% Bis-Tris SDS-PAGE gel. Membranes were subject to standard immunoblotting detection methods. IgG Antibodies used: Mouse IgG (Santa Cruz, Cat# sc-2025, RRID:AB_737182); Rabbit IgG (Santa Cruz, Cat# sc-2027, RRID:AB_737197).

GFP co-immunoprecipitations were performed on endogenously tagged HEK 293Ts seeded the previous day at a concentration of 10 ×10^6^ – 20 ×10^6^ cells per 15 cm. Five 15 cm dishes were collected per sample. Lysates were prepared as above, and supernatants were incubated with GFP-rabbit antibody coupled M270 epoxy beads for 2 hours at 4 °C. GFP co-immunoprecipitations were also performed on HEK 293Ts overexpressing either GFP-EV or GFP-hRTF2 cDNAs seeded the previous day at a concentration of 10 ×10^6^ – 20 ×10^6^ cells per 15 cm. Five 15 cm dishes were collected per sample. Chromatin extracts were isolated as previously described^71^. The supernatants were incubated with GFP-nanobody coupled M270 epoxy beads for 2 hours at 4 °C. GFP-rabbit or GFP-nanobodies were coupled to M270 epoxy beads according to manufacturer’s protocol. Beads were washed three times in 1 mL lysis buffer (5 minutes each on nutator). Beads were then eluted with 8 M urea, neutralized in TFA, and samples were submitted for LC-MS analysis at Rockefeller University’s Proteomics Core.

### Proteomics methods and data analysis

For the iPOND experiments, SDS-PAGE stack-type-bands were washed 3 times in 20% acetonitrile/50 mM ammonium bicarbonate followed by overnight washing in 50 mM ammonium bicarbonate. Proteins were reduced in 10 mM dithiothreitol at 57 °C which was hereafter removed and replaced with 45 mM iodoacetamide and incubated at room temperature in the dark. Proteins were digested overnight at room temperature where after digestion were halted by acidification (trifluoro acidic acid). Peptide was extracted three times in 20% acetonitrile and the extracts were merged and dried in speedvac. For the co-immunoprecipitations, sample were resuspended in 8 M urea (Cytiva PlusOne, Fisher Scientific) /50 mM ammonium bicarbonate (Fisher Scientific) /10 mM dithiothreitol (Millipore-Sigma). pH was verified to be neutral prior to incubation for 45 minutes. Reduced proteins were alkylated by incubating with 30 mM iodoacetamide in the dark for 45 min. Samples were diluted to less than 4 M Urea and digested by adding 0.5ug Endopeptidase LysC (Fujifilm Wako) overnight. Samples were further diluted to less than 2 M Urea and trypsinized (Promega) for 6 h. Digestions were halted by acidification (trifluoro acidic acid).

All sample types were desalted using reversed phase based micro solid phase extraction^72^. 3 of 10 uL were injected and analyzed by nano LC-MS/MS. Mass spectrometers ((Lumos Fusion coupled to a EasyLC1200 trap-free setup and Q-Exactive Plus coupled to a Dionex 3000 trap-based setup, Thermo Scientific) were mass calibrated weekly and operated with lock mass^73^. Both MS and MS/MS, were always operated in high (60,000 resolution)/high mode (30,000 and 15,000 resolution, respectively). Automatic Gain Control (AGC) for the Lumos Fusion were set to ‘Standard’ while the Q-Exactive Plus was set to 3e6 and 2e5 for MS and MS/MS). Samples were typically analyzed using 70 minute gradient typically increasing from 2% B/98%A to 42% B/58%A in 70 min (A: 0.1% formic acid, B: 80% acetonitrile/0.1% formic acid). Data were queried against the appropriate databases (UniProt’s human and mouse databases concatenated with common contaminants using Proteome Discoverer. 1.4/Mascot^74^. In short: 10 ppm or 20 ppm mass accuracy was used for precursor mass accuracy and 20 mDa for fragment ions. Carbamidomethylation of cysteines was set as a fixed modification and oxidation of methionine was always set as a variable modification. Protein N-term. acetylation and deamidation of asparagine and glutamine were considered for some of the samples. Search results were filtered requiring a Percolator calculated peptide false discovery rate of 1% or better and a mass accuracy of 5 ppm or better^75^. The average area of the three most abundant peptides matched to a given protein was used as a proxy for relative protein amount^76^.

### Live cell imaging

Cells were infected with retrovirus carrying a GFP-H2B cDNA. Cells were infected with Cre, seeded in 35 mm MatTek (MatTek, P35G-1.5-14-C) dishes and imaged every 10 minutes using Olympus CellVoyager (Olympus, CV1000).

### DNA combing

For unperturbed replication, exponentially growing cells were labeled with IdU (100 μM) followed by and CldU label (100 μM) with three warm PBS washes in between. Cells were collected and washed in PBS. Cells were resuspended in 45 μL Resuspension Buffer (PBS) with 0.2% NaN3. An equal volume of 2% low melting agarose Mb grade (BioRad) melted in resuspension buffer was equilibrated at 55 °C and added to cells to make agarose plugs. Agarose plugs were injected into digestion buffer (1 mg/mL proteinase K, 1% N-Lauroylsarcosine, 0.2% sodium deoxycholate, 100 mM EDTA, 10 mM Tris-HCl, pH 7.5) and incubated overnight at 55 °C. Plugs were washed for at least 3 ×1 hr each in TE 1X pH 8.5 with 100 mM NaCl before melting in 1 mL combing buffer at 68 °C for 20 minutes. After melting, tubes were transfered to 42 °C heat block. After 10 minutes, 1 μL beta-agarase (New England Bio Labs) was added without mixing and incubated overnight at 42 °C. DNA was poured into Disposable DNA Reservoirs (Genomic Vision) and combed onto silanized coverslips (CombiCoverslips, Genomic Vision or made in house) using the Molecular Combing System (Genomic Vision). Slides were dried for 2 hr at 65 °C. Slides were denatured with 0.5 M NaOH + 1 M NaCl for 8 minutes, followed by dehydration in 70%, 90% and 100% ethanol. Slides were blocked for 1 hr in 5% FBS in PBS. Slides were incubated with primary antibodies overnight at 4 °C or 2 hr at RT, washed and incubated with secondary antibody for 1 hr at RT. Slides were mounted with Fluoromount-G (SouthernBiotech, 0100-01). Fibers were imaged (Inverted Olympus IX-71 DeltaVision Image Restoration Microscope (Applied Precision)) and measured using FIJI software. Antibodies used: Mouse monoclonal anti-BrdU (B44), combing: 1:10 (BD Biosciences, Cat# 347580, RRID:AB_400326); Rat monoclonal anti-BrdU [BU1/75 (ICR1)], combing: 1:20 (Abcam, Cat# ab6326, RRID:AB_305426).

For the restart assays, cells were treated with IdU, 4 mM HU, and CldU for the indicated times. Three warm PBS washes were performed between each treatment. For PRIM1-AID experiments, cells were pulsed with indicated drugs for the following times: doxycycline for 4 hr and 15 minutes, IdU for 45 minutes, 4 mM HU for 120 minutes, CldU for 90 minutes, IAA for 120 minutes. Three warm PBS washes were done between each treatment, with doxycycline and IAA continued as indicated in the figures.

### Silanization of coverslips

Coverslips were prepared as previously published^77^ with modification of the plasma cleaning step, wherein we used Gatan Model 950 Advanced Plasma System for 10 minutes with atmospheric air.

### Metaphase spreads

Cells were treated for 40 hr with 0.2 aphidicolin μM. In the final 90 minutes of treatment, cells were co-incubated with colcemid (0.1 μg/mL). Cells were harvested and incubated in 5 mL 0.075 M KCL for 10 minutes before being fixed with the addition of 1 mL methanol and acetic acid (3:1). Cells were resuspended in 10 mL of methanol and acetic acid (3:1) overnight at 4 °C. Cells were dropped onto wet slides and dried at 42 °C for at least one hr before staining with 8% (v/v) KaryoMAX^TM^ Giemsa in 1x Gurr buffer for three minutes, washing in 1x Gurr buffer for 3 minutes, washing in water for 3 minutes, and drying. Dry slides were then imaged on the Metasystems Metafer slide scanning platform.

### RNase HII-digested neutral comet assay

Neutral comet assays were performed according to manufacturer’s protocol (Trevigen) with the addition of an RNase HII digestion step. Cells were harvested 72 hr after Hit & Run pMMP Cre retroviral infection^65^. Following cell lysis, slides were flooded with 1 X ThermoPol Buffer (NEB) for 5 minutes at 25 °C to equilibrate and digested with 10U RNase HII for 1 hr at 37 °C. To measure DNA breaks following RNase HII digestion, slides were stained with SYBR Green (1:10,000) in PBS. Comet tail moments were visualized or Axio Observer.A1 fluorescence microscope (Carl Zeiss), equipped with a Plan- Apochromat 40× NA-1.4 oil objective, the AxioCam CCD camera, and the AxioVision Rel Version 4.7 software and scored with OpenComet software^78^.

### Recombinant protein purification and co-immunoprecipitation experiments

hRTF2 and the hRNASEH2BCA (RNASEH2B is GST tagged) complexes were purified from *E.coli* using standard IMAC and GST pulldown, respectively, followed by further purification using an AKTA™ FPLC. hRTF2 and RNase H2 complex were incubated together at 4 °C for 2 hr in mixing buffer (20 mM HEPES, pH=7.5, 1 mM DTT, 0.01% NP-40, 5% glycerol, 0.1 mg/mL BSA, 1x cOmplete protease inhibitor, 50 mM NaCl; 50 uL total reaction). Benzonase was added at final concentration of 1 uL per 1 mL of mixing buffer. After mixing, an additional 1 uL of 5 M NaCl was added to each reaction to increase final NaCl concentration to 150 mM. Dynabeads™ (Invitrogen) were added to each reaction and incubated 4 °C for 1 hr. Beads were washed with 1 mL wash buffer (20 mM HEPES, pH=7.5, 1 mM DTT, 0.01% NP-40, 5% glycerol, 1x cOmplete protease 126 inhibitor, 150 mM NaCl) for 5 minutes. Washes were repeated four times before proteins were eluted in 20 uL Laemmli buffer at 100 °C for 5 minutes. Lysates were subjected to standard immunoblotting techniques. Sheep polyclonal anti-human RNase H2 complex antibody was a gift from Andrew Jackson & Martin Reijns^39^.

### Quantification and statistical analysis

All ANOVA and t-test analysis was done using Graphpad Prism software. Image quantification was completed with FIJI software. For DNA combing restart assays, outliers were identified and removed with ROUT (1%). Descriptions of the statistical analyses presented in the figures can be found in the corresponding figure legends.

### Reporting summary

Further information on research design is available in the Nature Portfolio Reporting Summary linked to this article.

### Supplementary information

Supplementary Information

Peer Review File

Description of Additional Supplementary Files

Supplementary Data 1

Supplementary Data 2

Supplementary Data 3

Supplementary Movie 1

Supplementary Movie 2

Supplementary Movie 3

Reporting Summary

### Source data

Source Data

## Data Availability

Mouse RNA sequencing data have been deposited in NCBI’s Gene Expression Omnibus and are accessible through GEO Series accession number GSE152047. Sequence and transcript coordinates for mouse mm10 UCSC genome and gene models are available from the Bioconductor Bsgenome.Mmusculus.UCSC.mm10 (https://bioconductor.org/packages/release/data/annotation/html/BSgenome.Mmusculus.UCSC.mm10.html) and TxDb.Mmusculus.UCSC.mm10.knownGene https://bioconductor.org/packages/release/data/annotation/html/TxDb.Mmusculus.UCSC.mm10.knownGene.html Bioconductor libraries respectively. Raw data from the proteomic studies are not available, but the complete primary data is included in Supplementary Data 1-3. Source data are provided with this paper.
