# Supplementary material for: RTF2 controls replication repriming and ribonucleotide excision at the replisome
Source: Nat Commun. 2024 Mar 2;15:1943. doi: 10.1038/s41467-024-45947-z (PMC10908796; doi:10.1038/s41467-024-45947-z)
Supplement: Supplementary file 1 — Supplementary Information [file 41467_2024_45947_MOESM1_ESM.pdf]

Supplementary Information

Title: RTF2 controls replication repriming and ribonucleotide excision at the replisome

Brooke A. Conti[1*], Penelope D. Ruiz[1*], Cayla Broton[1*], Nicolas J. Blobel[1], Molly C. Kottemann[1], Sunandini Sridhar[1], Francis P. Lach[1], Tom Wiley[1], Nanda K. Sasi[2], Thomas Carroll[3], & Agata Smogorzewska[1]

Index of Supplementary information:

1. 3 Supplementary movies……………………………….............................page 3
2. Supplementary Figure 1………………………………………………………page 4
3. Supplementary Figure 2………………………………………………………page 5
4. Supplementary Figure 3………………………………………………………page 6
5. Supplementary Figure 4………………………………………………………page 7
6. Supplementary Figure 5………………………………………………………page 8
7. Supplementary Figure 6………………………………………………………page 9
8. Supplementary Figure 7……..………………………………………………page 10
9. Supplementary Figure 8……..…..…………………………………………..page 11
10. Supplementary Figure 9…………………………………………………..…page 12
11. Supplementary Figure 10……………………………………………………page 13
12. Supplementary Data 1……………………………………………….…….Excel file
13. Supplementary Data 2……………………………………………….…….Excel file
14. Supplementary Data 3……………………………………………….…….Excel file
15. Supplementary Table 1
    - Mouse strains……………………………………………………………page 14
    - Mammalian cell lines……………………………………………………page 14
    - RTF2 mouse genotyping primers …..…………………………………..page 14
    - RT-qPCR primers……..……………………...…...………………………...…page 15
    - Gateway cloning primers ………………………………………………..page 15
    - Mutagenesis primers …………..………………………..…………..page 16
    - CRISPR cloning primers ……………...…………………………………..page 16
    - siRNAs and shRNAs ……………………..………………………………..page 16
    - Plasmids …………………………………………………………...……..page 17
    - Antibodies………………………………………………………………..page 18
    - Reagents………………………………………………………...……..page 20
    - Equipment……………………..………………….……………...……..page 23
16. Supplementary References…………………………………………………...page 24

Movies
All movies show representative mitoses in SV40-LT-immortalized *Rtf2*$^{-/-}$ MEFs.

Supplementary Movie 1 Normal mitosis.
Supplementary Movie 2 Failed mitosis generating anaphase bridged and lagging chromosomes.
Supplementary Movie 3. Another failed mitosis whereby the cell does not divide and instead forms a ‘donut.’

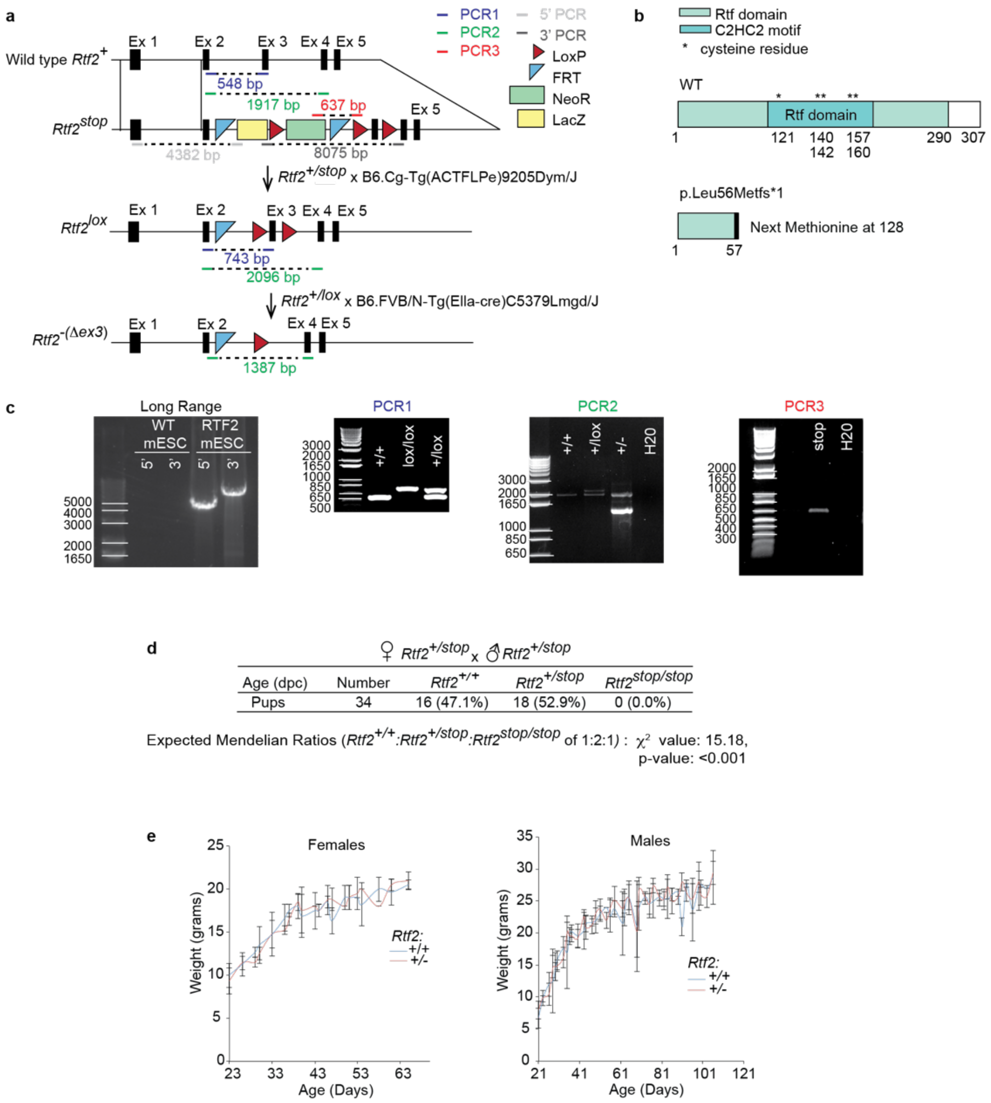

Supplementary Figure 1. RTF2 is necessary for *in vivo* viability.

a, Schematic of crosses to generate the genetic knockout of RTF2 in *Mus musculus*. Mouse embryonic stem cells (mESCs) containing an $Rtf2^{tm1a(KOMP)Wtsi}$ ($Rtf2^{stop}$) allele (Knockout Mouse Project [KOMP]) were injected into mouse blastocysts (B6(Cg)-Tyr^{c-2J}/J, Jackson Labs) to create chimeric mice, which through appropriate crosses generated $Rtf2^{+/stop}$ mice. $Rtf2^{+/stop}$ mice were bred with mice expressing Flp recombinase and subsequently mice expressing Cre recombinase under a ubiquitous promoter. These crosses induced the loss of exon 3 to yield $Rtf2^{+/\Delta\ exon3}$ pups. The loss of exon 3 will herein be referred to as $Rtf2^{-}$. Mice with different *Rtf2* genotypes were maintained on C57BL/6 background. PCR primers and product sizes are indicated on the *Rtf2* alleles. **b**, Schematic of WT RTF2 protein. Loss of exon 3 results in early truncation of RTF2. **c**, Representative genotyping PCR products run on 0.8% agarose gel and stained with ethidium bromide. Long range PCR products amplified from the KOMP mESCs confirmed the presence of the FRT and LoxP sites after excision and analysis with Sanger sequencing. PCR1, PCR2, and PCR3 products amplified from DNA extracted from mouse tail tips were used to confirm genotypes. **d,** Genotypes from litters of $Rtf2^{+/stop}$ female mice crossed with $Rtf2^{+/stop}$ male mice. $Rtf2^{stop/stop}$ mice showing embryonic lethality. **e,** Weights from $Rtf2^{+/-}$ and $Rtf2^{+/+}$ mice. For each point, n$\geq$2 mice. Total n for the indicated genotypes were: 15 for female $Rtf2^{+/+}$, 21 for female $Rtf2^{+/-}$, 14 for male $Rtf2^{+/}$, and 34 for male $Rtf2^{+/-}$. In d. Chi-squared test statistic (X^2) and p-values are indicated for pups. In e, error bars indicate standard deviation. WT = wildtype. Source data are provided as a Source Data file.

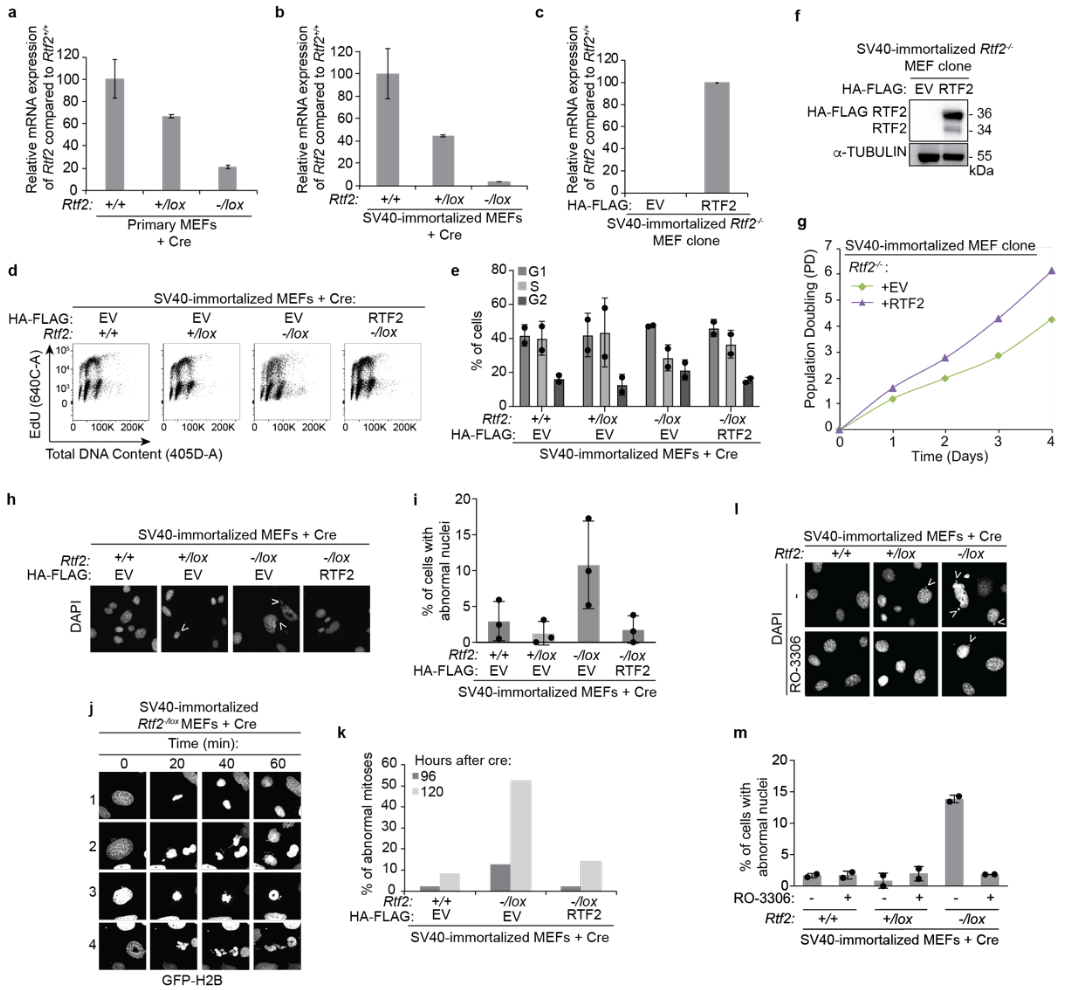

Supplementary Figure 2. RTF2-deficient SV40-immortalized MEFs and clones accumulate aberrant nuclear structures that are suppressed by CDK1 inhibition. a,b,c, Representative RT-qPCR of relative *Rtf2* mRNA transcript levels in primary MEFs (a) or SV40-immortalized (b) MEFs at 96 hr after Cre, or from an SV40-immortalized MEF *Rtf2*$^{-/-}$ clone expressing empty vector (EV) or RTF2 cDNA (c). *Rtf2* expression was normalized to *β-actin* expression. **d,** Representative cell cycle profiles from flow cytometry of indicated SV40-immortalized MEFs at 72 hr after Cre. **e,** Average percentage of G1, S phase, and G2 cells from (d). **f,** Representative immunoblot of whole cell lysates in SV40-immortalized RTF2-deficient sub-cloned MEF lines expressing empty vector (EV) or HA-FLAG-mRTF2 (RTF2) cDNA constructs. α-tubulin represents loading control. **g,** Representative growth curves of SV40-immortalized RTF2-deficient sub-cloned MEF lines expressing EV or HA-FLAG-mRTF2. **h,** Representative images of DAPI staining from indicated SV40-immortalized MEFs expressing EV or HA-FLAG-mRTF2 at 120 hr after Cre. Arrows indicate abnormal nuclei. **i,** Quantification of percentage of cells with abnormal nuclei based on DAPI staining from (h). **j,** Representative images of GFP-H2B staining in live *Rtf2*$^{-/-}$ SV40-immortalized MEFs expressing empty vector (EV) and transduced with pWZL Cre-hygro retrovirus 120 hrs prior to analysis. In row 1, a cell enters mitosis and undergoes a normal division. In rows 2-4, cells fail to complete successful mitosis, resulting in lagging chromosomes and/or abnormal nuclear morphology. **k,** Quantification of abnormal mitoses from live-cell imaging of cells in (j). **l,** Representative images of DAPI-stained nuclei from SV40-immortalized MEFs at 120 hr after Cre and then treated with CDK1 inhibitor (RO-3306) for an additional 24 hr. Arrows indicate abnormal nuclei. **m,** Quantification of abnormal nuclei in images shown in (l). Experiments were conducted at least three times in biological replicates with technical triplicates for a-c. Representative plot of normalized average across technical triplicates is shown, with error bars indicating standard deviation, for a-c. Experiments were conducted at least three times in biological replicates with consistent results for d-i,l,m. Averages from two biological replicates plotted in e, with error bars representing standard deviation. EV = empty vector. Source data are provided as a Source Data file.

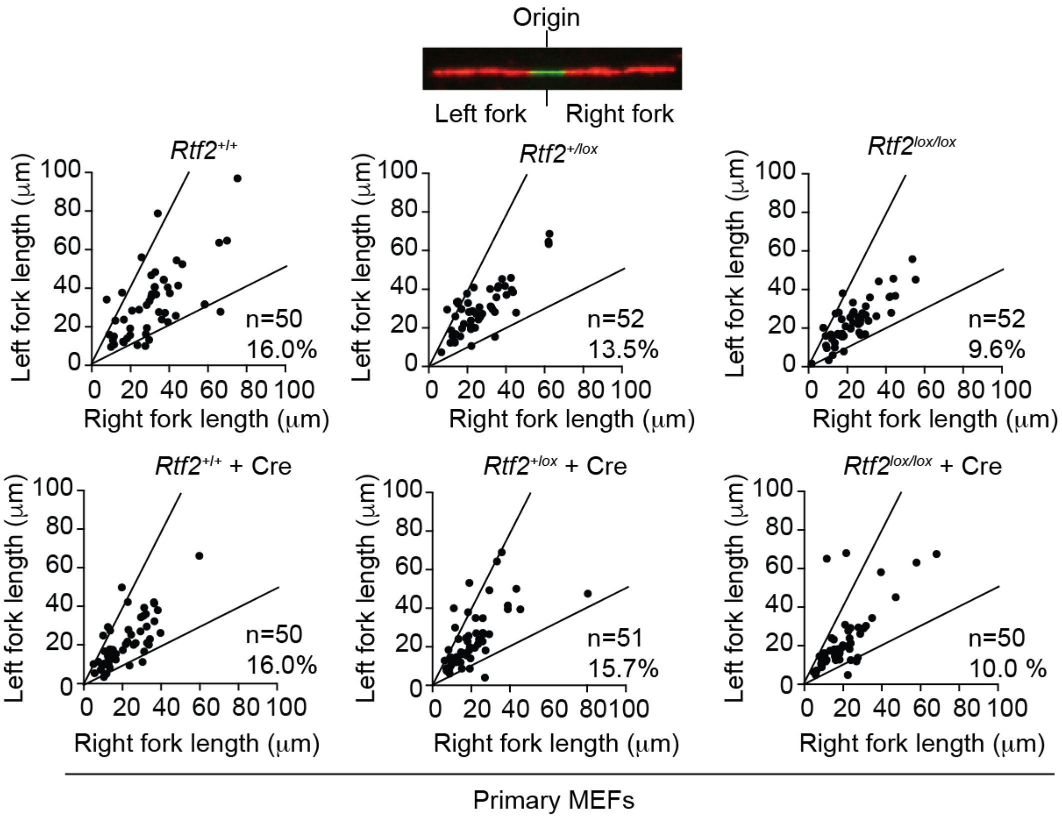

Supplementary Figure 3. RTF2-deficient replication forks progress symmetrically from origins.
a, Top: Schematic of replication initiation sites, identified as species where the second label (CldU) flanks the first label (IdU). Left fork length is plotted against right fork length to determine fork symmetry. Bottom: Representative experiment showing fork symmetry from primary MEFs 72 hrs after transduction with Cre. Left fork length is plotted against right fork length. Lines represent arbitrary cutoffs for replication forks with symmetry less than 2 and greater than 0.5. Percentages in the bottom right corner represent the percentage of asymmetric forks. Source data are provided as a Source Data file.

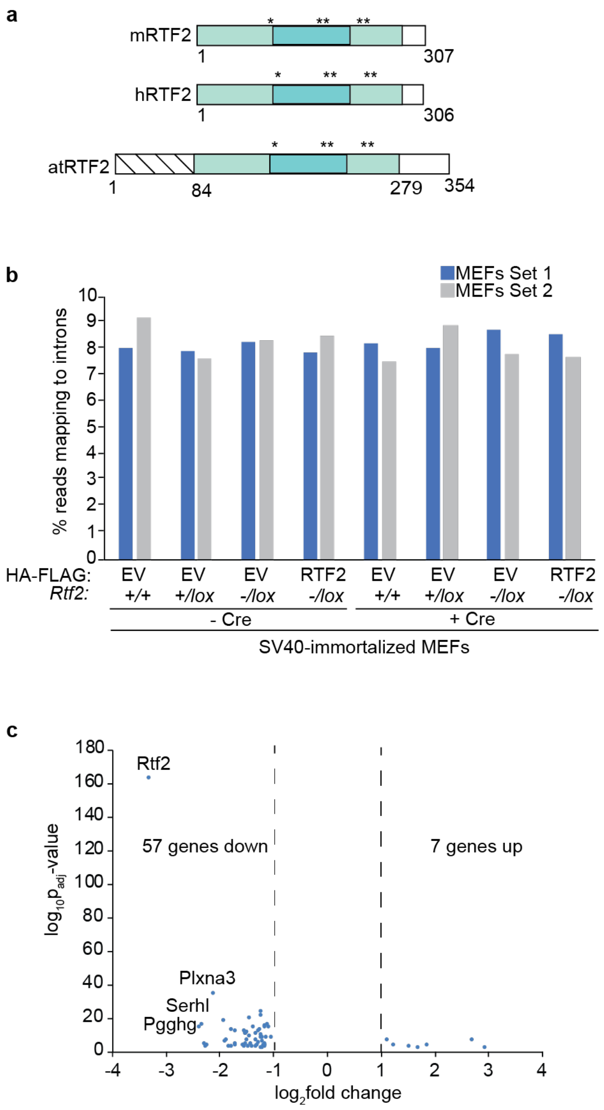

Supplementary Figure 4. RTF2-deficient MEFs do not display changes in global intron retention or gene expression.

a, Schematic of RTF2 from *Mus musculus, Homo sapiens* and *Arabidopsis thaliana*. Amino acids 7-63 in atRTF2 are implicated in an intron-retention defect in plants. **b,** Percent of reads from paired-end RNA-seq mapping to introns. Genotypes are indicated for SV40-immortalized MEFs expressing cDNA for HA-FLAG-empty-vector (EV) or HA-FLAG-mouse-RTF2 (RTF2) 120 hrs after transduction with pWZL Cre-hygro retrovirus **c,** Volcano plot showing $\log_{10}$p-values against $\log_2$fold change for the significant differentially expressed genes as calculated by DESeq2. Genes with $\log_2$fold change >1 and $p_{adjusted}$-value <0.05 averaged across the two biological replicates (technical triplicate). These results indicate 57 genes significantly downregulated and 7 genes significantly upregulated. n=3 for each set of MEFs, technical replicate. Comparison is between aligned single-end reads from *Rtf2*$^{+/+}$ and *Rtf2*$^{-/lox}$ SV40-immortalized MEFs transduced with Hit & Run pMMP Cre retrovirus 72 hrs prior to harvest. Source data are provided as a Source Data file.

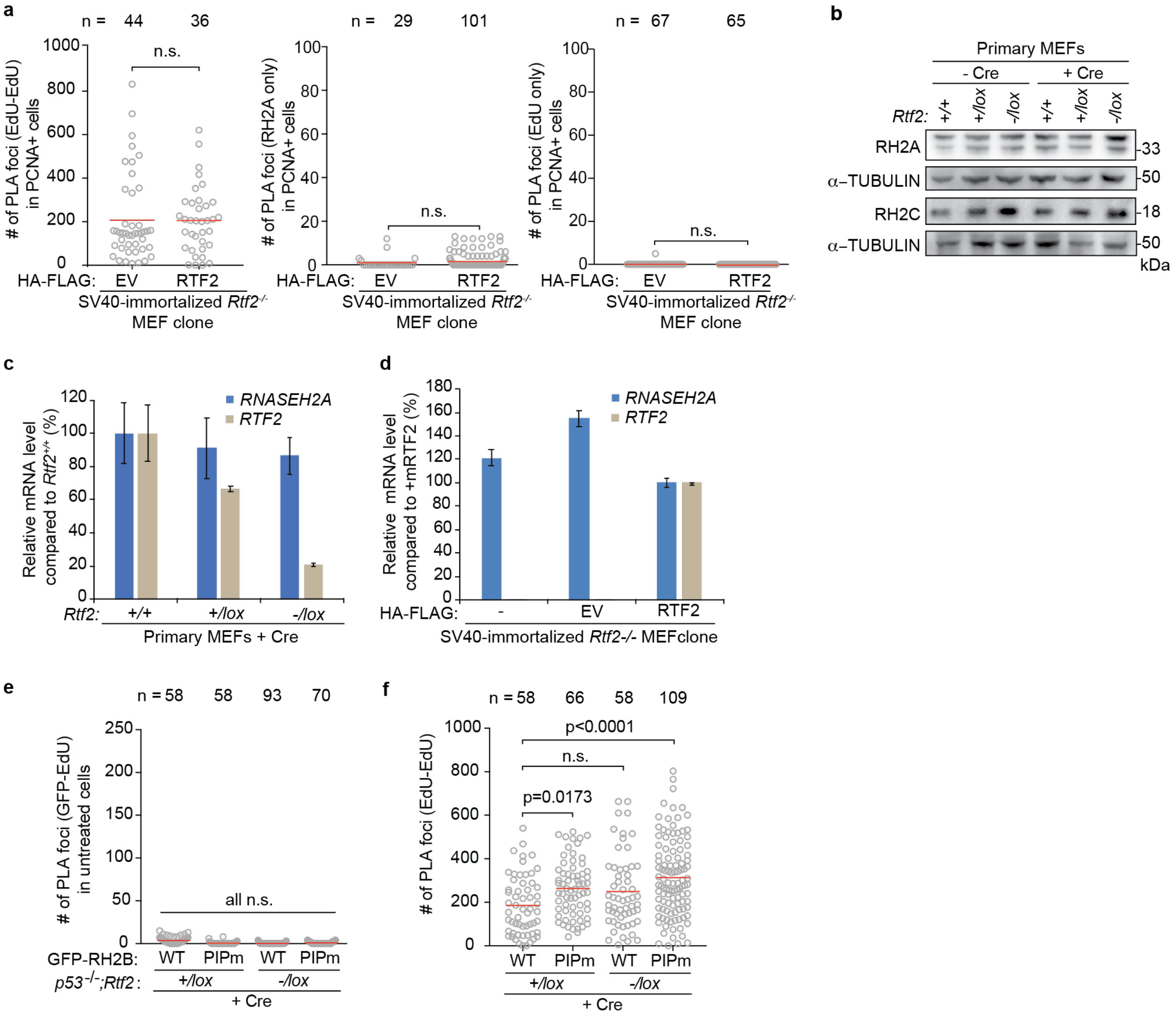

Supplementary Figure 5. RTF2 deficiency results in loss of RNase H2 from the replication fork. **a,** Quantification of EdU-EdU PLA foci and single antibody (RNASEH2A and EdU) controls for nPLA in Fig. 3d. **b,** Representative immunoblot of whole cell lysates showing RNASEH2A and RNASEH2C levels in primary MEFs transduced with Hit & Run Cre recombinase retrovirus 72 hrs before harvest. **c,d,** RT-qPCR analysis of relative mouse *Rnaseh2a* and *Rtf2* transcript levels in primary MEFs transduced with Hit & Run pMMP Cre retrovirus 72 hrs before harvest or in SV40-LT immortalized RTF2-deficient sub-cloned MEF lines expressing HA-FLAG empty vector (EV) or mRTF2 (RTF2) cDNA constructs, respectively. Expression is normalized to *β-actin*. **e,** Quantification of GFP (RNASEH2B)-EdU PLA foci in untreated cells (no EdU) for nPLA in Fig. 4a. **f,** Quantification of EdU-EdU PLA foci for nPLA in Fig. 4a. Experiments were conducted at least three times in biological replicates with consistent results for a,b. Experiment conducted twice in biological replicates with consistent results for e,f. Experiments were conducted at least three times in biological replicates with technical triplicates for c,d. Representative plot of normalized average across technical triplicates is shown, with error bars indicating standard deviation, for c.d. Mean is indicated with a red line for a,e,f. Significance evaluated by Kruskal-Wallis ANOVA with a Dunn's post-test. EV = empty vector, RH2A = RNASEH2A, RH2C = RNASEH2C, WT = wildtype, PIPm = PIP box mutant. Source data are provided as a Source Data file.

a **b** **c** **d**

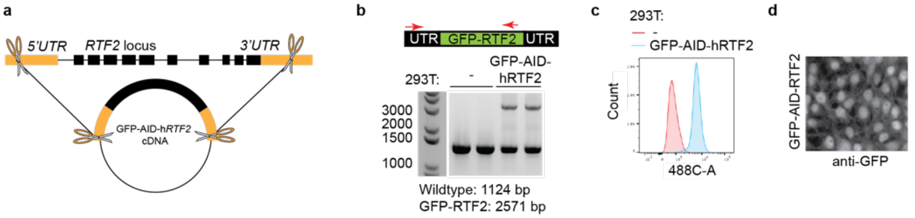

e

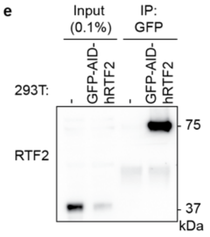

f

	IP: GFP			
	#PSM		Area Under Curve	
	293T	GFP-AID-RTF2	293T	GFP-AID-RTF2
RTF2	0	7.5	0.00	1.11E8
RH2A	0.5	1.5	0.00	1.50E7
MCM2	0.5	2.5	9.84E5	6.14E6
MCM3	0	2.5	0.00	1.13E7
MCM4	0.5	1.5	2.69E6	1.15E7
MCM5	0.5	1.5	0.00	1.36E7
MCM6	0.5	4.5	2.14E6	9.14E6
MCM7	2	5	6.10E6	1.70E7
PCNA	5.5	7.5	9.25E7	2.31E8
RPA70	1	4	0.00	2.97E7
RPA32	0	1	0.00	9.70E6
RPA14	0	0.5	0.00	5.62E6

g

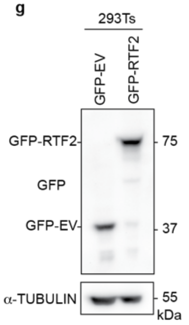

h

	IP: GFP			
	#PSM		Area Under Curve	
	GFP-EV	GFP-RTF2	GFP-EV	GFP-RTF2
RTF2	0	30	0.00	4.48E9
GFP-RTF2	0	60.5	0.00	4.97E9
RH2A	0	1.5	0.00	4.96E7
RH2C	0	0.5	0.00	9.88E6
MCM2	7.5	13.5	3.16E7	9.58E7
MCM3	7.5	14.5	1.518E7	6.26E8
MCM4	11	13.5	4.72E7	1.98E8
MCM5	3.5	3	5.76E7	7.25E7
MCM6	4	17	2.03E7	8.84E7
MCM7	7.5	16	5.21E7	1.79E8
PCNA	3.5	7	5.17E7	1.62E8
RPA70	3.5	8	3.10E7	7.07E7
RPA32	4.5	7	4.40E7	1.28E8
RPA14	0.5	2.5	9.22E7	2.31E8

i

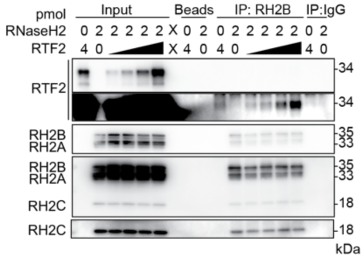

Supplementary Figure 6. RTF2 interacts with RNase H2 and components of the replisome.
a, Schematic of CRISPR-Cas9 targeting to generate a tagged RTF2 construct expressed from the endogenous *RTF2* locus. A plasmid carrying a GFP-AID-h*RTF2* cDNA flanked by homology arms to the 5'UTR and 3'UTRs (orange boxes) of *RTF2* was targeted to the endogenous locus of Rtf2 in HEK293T cells and subsequently cloned. This line will be referred to as endogenous GFP-AID-RTF2 HEK293Ts. **b,** Genotyping analysis of wild type and endogenous GFP-AID-*RTF2* HEK293Ts. Schematic represents genotyping primers that were used to amplify the endogenously tagged locus. Forward primer recognizes *RTF2* promoter region upstream to the GFP-AID-h*RTF2* insert and reverse primer recognizes an *RTF2* exonic region. The primer pair amplifies a wild type *RTF2* allele of 1124 bp and the GFP-tagged allele of 2571 bp. **c,** Flow cytometry analysis of wild type and endogenous GFP-AID-RTF2 HEK293Ts. **d,** Representative images of immunofluorescence analysis of endogenous GFP-AID-RTF2 HEK293Ts. Cells were fixed and stained with anti-GFP antibodies. **e,** Representative immunoprecipitation with GFP antibodies from wild type and endogenous GFP-AID-RTF2 HEK293Ts. Immunoblotted with RTF2 antibody. The GFP-AID-h*RTF2* protein is predicted to be 90.5 kDa. **f,** GFP was immunoprecipitated with GFP antibodies from wild type and endogenously targeted GFP-AID-RTF2 HEK293Ts described in a-e. Average peptide spectral matches (#PSM) and area under the curve (AUC) from LC-MS for given proteins averaged across two biological replicates. **g,** Representative immunoprecipitation with GFP from HEK293Ts retrovirally expressing GFP-Empty Vector (GFP-EV) or GFP-human-RTF2 (GFP-RTF2). The GFP-h*RTF2* construct is predicted to be 63.8 kDa. **h,** GFP was immunoprecipitated from the chromatin fraction of cell lysates with GFP nanobodies isolated from cells in g. Average #PSM and AUC from LC-MS for given proteins averaged across two biological replicates **i,** Representative immunoblot from immunoprecipitation of recombinant RNase H2 complex and RTF2 expressed in *E. coli*. Protein amount (pmol) are indicated above each lane; range of RTF2 is 0.5, 1, 2, 4 pmol. The blot corresponds to immunoblot shown in Fig. 4e. Experiments were conducted at least two times in biological replicates with consistent results for f,h,i. EV = empty vector, RH2A = RNASEH2A, RH2B = RNASEH2B, RH2C = RNASEH2C. Source data are provided as a Source Data file.

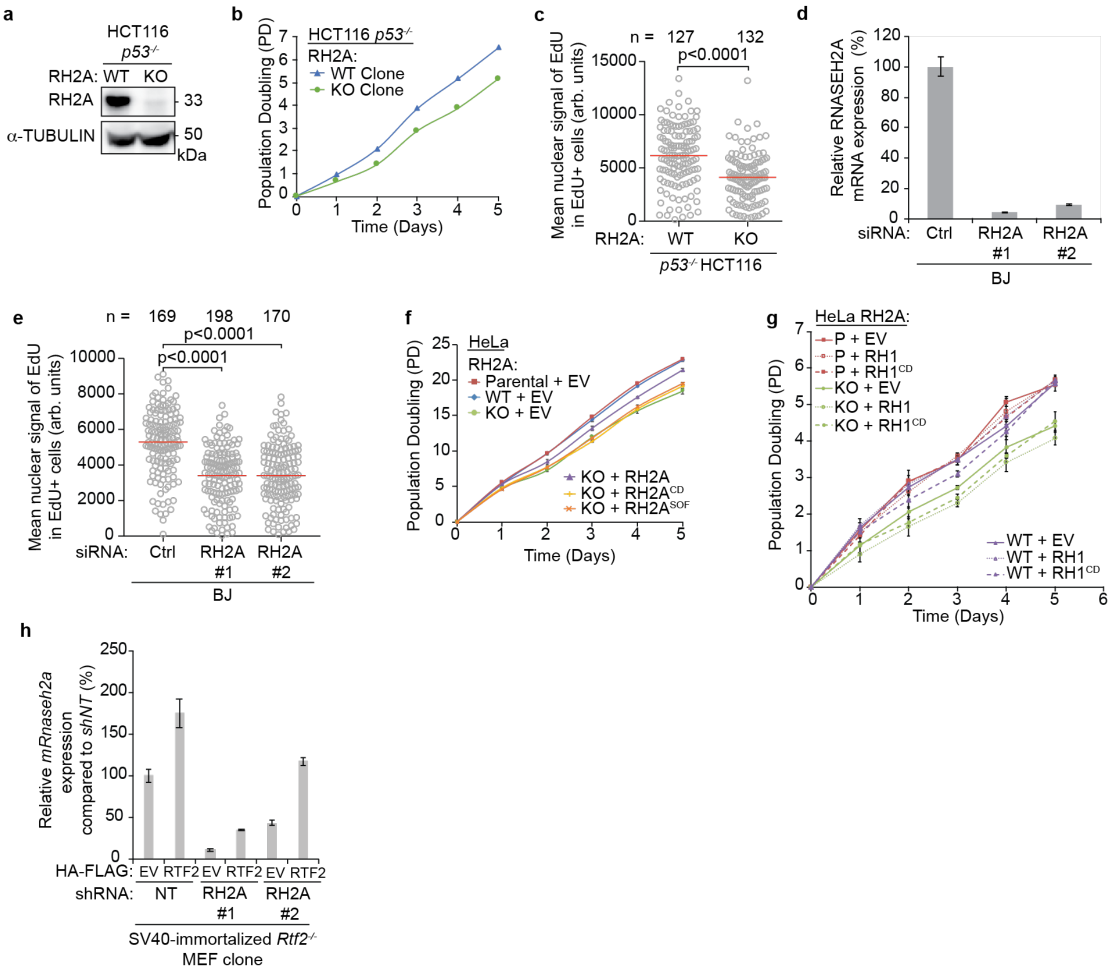

Supplementary Figure 7. RNase H2-deficient cells phenocopy growth and replication phenotypes of RTF2 deficiency.

a, Representative immunoblot showing loss of RNASEH2A in CRISPR-edited HCT116 *p53*$^{-/-}$ cells. α-tubulin represents loading control. **b,** Growth curves of indicated cells. **c,** Quantification of representative experiment showing mean signal of EdU in EdU-positive cells in indicated cells. **d,** Representative RT-qPCR analysis of relative human *RNASEH2A* transcript levels in BJ cells. Expression is normalized to *β-actin* expression. **e,** Mean nuclear signal of EdU in EdU-positive BJ cells treated with indicated siRNAs. **f,** Growth curves of HeLa cells and CRISPR-edited RNASEH2A KO HeLa cells complemented with wildtype, catalytic dead (RH2A^{CD}/RNASEH2A$^{D34A;D169A}$), or separation of function (RH2A^{SOF}/RNASEH2A$^{P40D;Y210A}$) RNASEH2A. **g,** Growth curves of HeLa cells and CRISPR-edited RNASEH2A KO HeLa cells expressing empty vector (EV), wildtype V5-RNASEH1 (RH1), or catalytic dead (RH1^{CD}/V5-RNASEH1^{D210N}) RNASEH1. **h,** Representative RT-qPCR analysis of mouse *Rnaseh2* expression in SV40-immortalized *Rtf2*$^{-/-}$ MEF clones expressing empty vector (EV) or HA-FLAG-mRTF2 (RTF2) transduced with indicated shRNAs. *Rnaseh2* expression is normalized to *b-actin*. Experiments were conducted at least three times in biological replicates with consistent results for a,c,e. Experiments were conducted at least three times in biological replicates with technical triplicates for d,h. Representative plot of normalized average across technical triplicates is shown, with error bars indicating standard deviation, for d,h. Experiments were conducted three times in biological replicates with technical triplicates for b,f,g. Error bars represent standard deviation. Each dot represents one cell for c,e. Mean for each sample shown with red line for c,e. Cells were pulsed with EdU for 1 hr prior to fixation for c,e, Experiments were blinded prior to analysis for c,e. Significance evaluated by Kruskal-Wallis ANOVA with a Dunn's post-test. RH2A = RNASEH2A, P = Parental, WT = wildtype, EV = empty vector, RH2A^{CD} = catalytic dead RNASEH2A$^{D34A/D169A}$, RH2A^{SOF} = separation of function RNASEH2A$^{P40D/Y210A}$, RH1 = RNASEH1, CD = catalytic dead RNASEH1^{D210N}, Ctrl = Control, NT = Non-targeting. Source data are provided as a Source Data file.

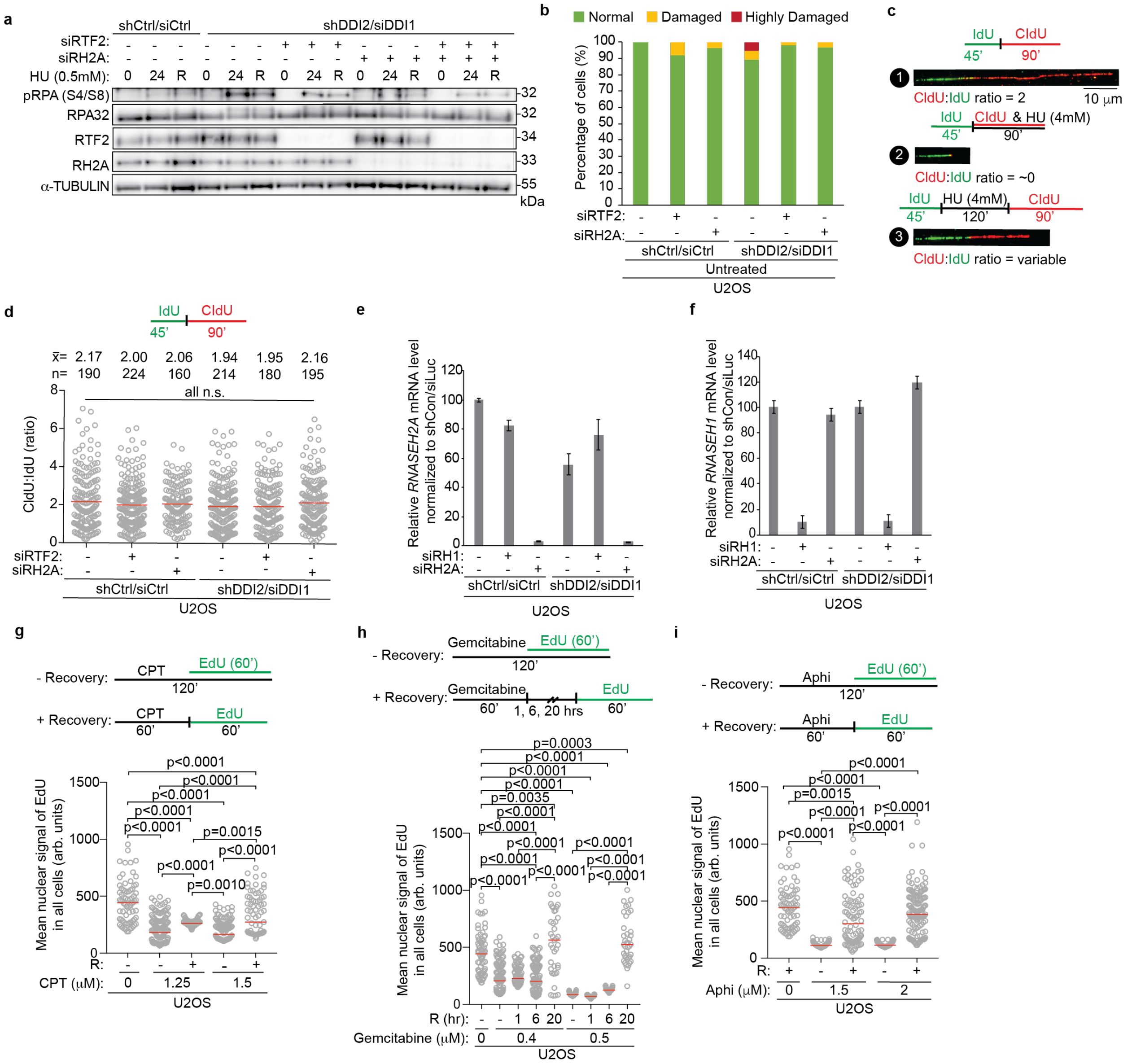

Supplementary Figure 8. Removal of RNase H2 from stalled replication forks allows for replication restart and genome stability.

a, Representative immunoblot in U2OS cells transduced or transfected with indicated RNAi reagents and treated with HU (0 = untreated, 24 = 24 hr treatment, R = 24 hr treatment followed by 8 hr release). **b,** Quantification of representative experiment of the percentage of metaphase spreads in each category described in Fig. 7c in U2OS cells transduced or transfected with indicated RNAi reagents (112 hrs). n>50 for metaphases scored in each sample. **c,** Schematic and representative images of DNA combing replication fork restart assay. PBS washes are indicated by a black vertical line in all schematics. **d,** Top: Labeling schematic. Bottom: Ratio of CldU tract to IdU tract lengths in U2OS cells transfected with indicated siRNAs for 72 hrs. **e,f,** RT-qPCR analysis of *RNASEH2A* or *RNASEH1* expression in indicated cells normalized to *GAPDH*. **g, h, i,** Top: Labeling schematic. Mean nuclear signal of EdU in EdU-positive U2OS cells treated as indicated. Experiments conducted at least three times in biological replicates with consistent results for a,b,d-i. Error bars represent standard deviation. Experiments were blinded prior to analysis for d. Average CldU:IdU ratios are listed above each sample for d, g-i. Outliers removed with ROUT (1%) for d. Each dot represents one cell for g-i. Mean shown with red line for d, g-i. Experiments were conducted at least three times in biological replicates with technical triplicates for h,i. Representative plot of normalized average across technical triplicates is shown, with error bars indicating standard deviation, for e,f. Significance evaluated by Kruskal-Wallis ANOVA with a Dunn's post-test. RH2A= RNASEH2A, Ctrl = Control, Luc = Luciferase, RH2A = RNASEH2A, Aphi = Aphidicolin, CPT = Camptothecin, RH1 = RNASEH1. Source data are provided as a Source Data file.

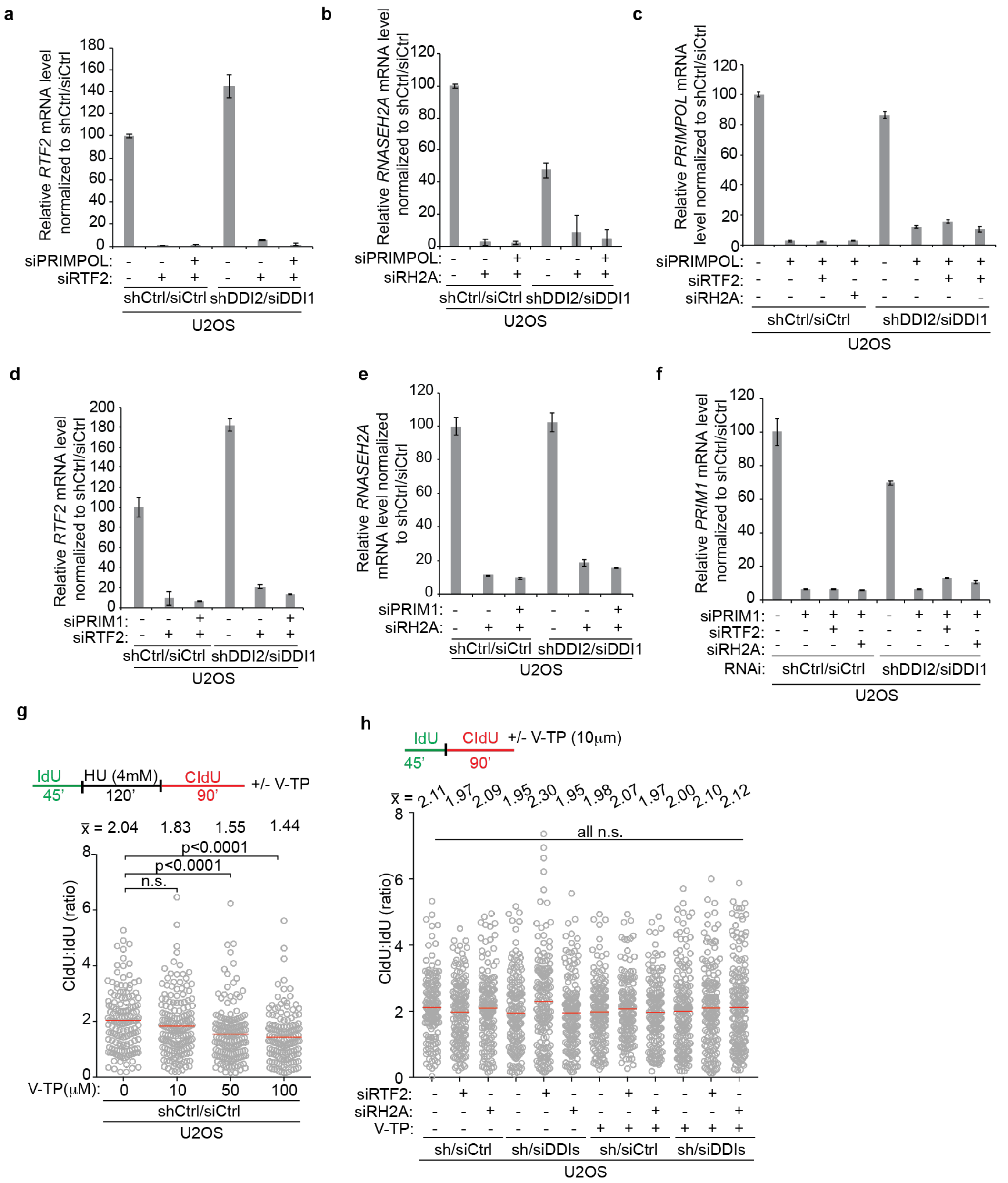

Supplementary Figure 9. Catalytic activity of primase PRIM1, not PRIMPOL, is required for efficient replication restart after stress.

a-c, Representative RT-qPCR analysis of relative human *RNASEH2A, RTF2,* or *PRIMPOL* transcript levels in indicated U2OS cells. Expression is normalized to *GAPDH*. **d-f,** Representative RT-qPCR analysis of relative human *RNASEH2A, RTF2,* or *PRIM1* transcript levels in indicated U2OS cells. Expression is normalized to *GAPDH*. **g,h,** Top: Labeling schematics for DNA combing restart and progression assays in the setting of varying concentrations of V-TP, a potent PRIM1 inhibitor. PBS washes are indicated by a black vertical line in all schematics. Bottom: Quantification of representative experiment of CldU:IdU tract length ratios in U2OS cells transduced or transfected with indicated RNAi reagents. Experiments were conducted at least three times in biological replicates with technical triplicates for a-f. Representative plot of normalized average across technical triplicates is shown, with error bars indicating standard deviation, for a-f. Experiments were conducted at least three times in biological replicates with consistent results for g,h. Experiments were blinded prior to analysis for g,h. Mean is shown with red line for g,h. Average CldU:IdU ratios are listed above each sample for g,h. Outliers removed with ROUT (1%) for g,h. Significance evaluated by Kruskal-Wallis ANOVA with a Dunn's post-test. RH2A= RNASEH2A, Ctrl = Control. Source data are provided as a Source Data file.

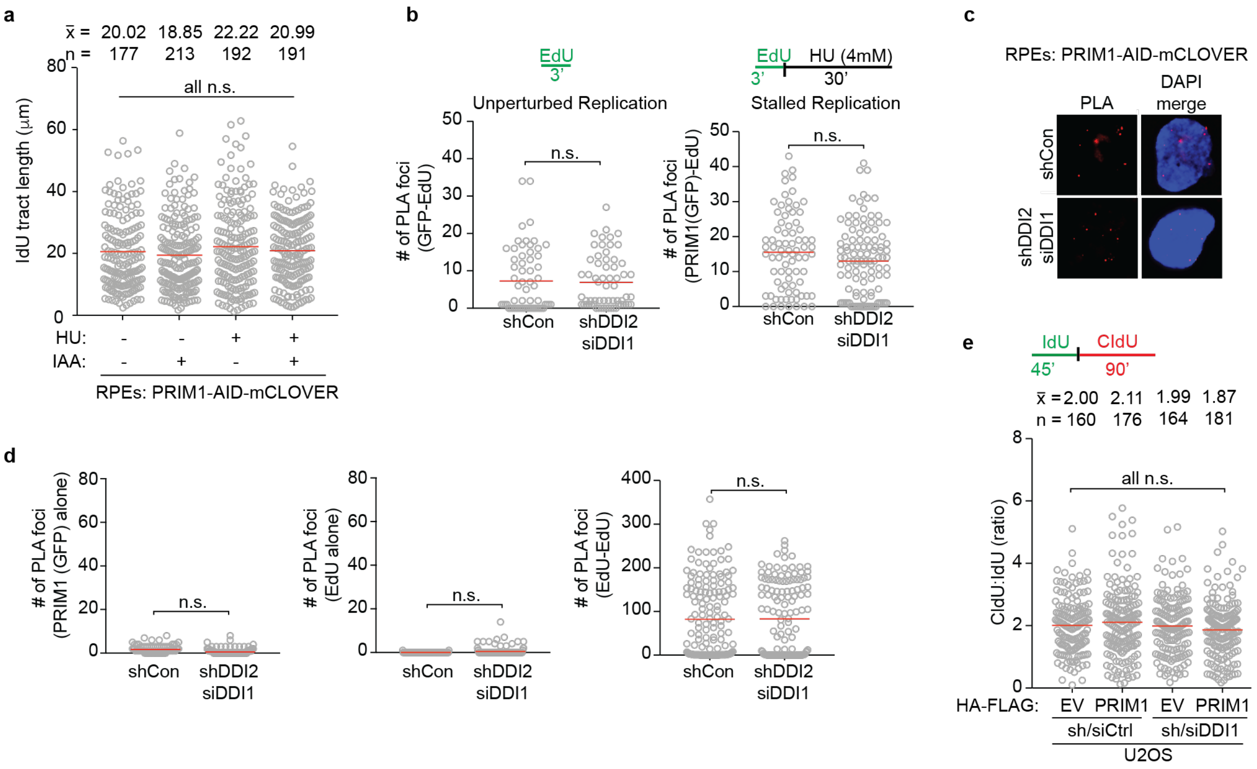

Supplementary Figure 10. Replication restart is sensitive to cellular levels of PRIM1.
a, IdU lengths in indicated RPE cells from experiment in Fig. 9b. Mean for each sample shown with red line. Average length indicated above each sample. **b,** Top: nPLA labeling schematic. PBS washes are indicated by a black vertical line in all schematics. Bottom: Quantification of PRIM1(GFP)-EdU PLA foci in PRIM1-AID-mCLOVER RPE cells in the setting of progressing or stalled replication. **c,** Representative images of PRIM1(GFP)-EdU foci in PRIM1-AID -mCLOVER RPE cells. DAPI stains the nucleus. **d,** Quantification of single antibody (PRIM1 (GFP) and EdU) and EdU-EdU PLA foci controls for nPLA in Supplementary Fig. 10b. **e,** Top: Labeling schematic for DNA combing. PBS washes are indicated by a black vertical line in all schematics. Below: Quantification of representative experiment of CldU:IdU tract length ratios in U2OS cells transduced with either HA-FLAG-PRIM1 or HA-FLAG-EV and transduced or transfected with indicated RNAi reagents related to Fig. 9e. Experiments were conducted at least three times in biological replicates with consistent results for a-e. Mean shown with red line for a,b,d,e. Experiments were blinded prior to analysis for a ,e. Average CldU:IdU ratios or tract length are listed above each sample for a,e. Outliers removed with ROUT (1%) for a,e. Significance evaluated by Kruskal-Wallis ANOVA with a Dunn's post-test. Ctrl = Control, RH2A = RNASEH2A, IAA = auxin, EV = empty vector. Source data are provided as a Source Data file.

Supplementary Data 1. Proteomic data from the iPOND experiment shown in Figure 3. **Excel file**

Supplementary Data 2. Proteomic data from the IP experiments from HEK293T cells expressing endogenously GFP- tagged RTF2 shown in Supplementary Figure 6f. **Excel file**

Supplementary Data 3. Proteomic data from the IP experiments from HEK293T cells expressing exogenously GFP- tagged RTF2 shown in Supplementary Figure 6h. **Excel files**

Supplementary Table 1. List of reagents and equipment used in the study

Mouse strains		
**MOUSE STRAIN**	**SOURCE**	**IDENTIFIER**
$Rtf2^{tm1a(KOMP)wtsi}$	This paper	
B6.Cg Tg(ACTFLPe)9205Dym/J	Jackson Laboratories	005703
B6.FVB/N-Tg(Ella-cre)C5379Lmgd/J	Jackson Laboratories	003314
C57BL/6J	Jackson Laboratories	000664

Mammalian cell lines		
**CELL LINE**	**SOURCE**	**IDENTIFIER**
Human: HEK 293T	ATCC	
Human: HEK 293T endogenously GFP-tagged RTF2	This paper	N/A
Human: BJ-hTERT-E6/7, male	Smogorzewska Lab	
Human: U2OS, female	ATCC	HTB-96
Human: RPE $p53^{-/-}$, $pRb^{-/-}$, PRIM1-AID-mClover	de Lange Lab	
Human: HeLa (HeLa Parental, HeLa WT Clone, HeLa RNASEH2A KO Clone), female	Durocher and Jackson Labs	
Human: HCT-116 $p53^{-/-}$ (HCT-116 $p53^{-/-}$ WT Clone, HCT-116 $p53^{-/-}$ RNASEH2A KO Clone)	Durocher and Jackson Labs	
Mouse: RTF2 MEFs ($Rtf2^{+/+}$, $Rtf2^{+/lox}$, $Rtf2^{-/lox}$, and $Rtf2^{lox/lox}$, SV40-immortalized $Rtf2^{-/-}$ clone, $p53^{-/-};Rtf2^{+/lox}$, $p53^{-/-};Rtf2^{-/lox}$)	This paper	N/A
KOMP mES cells $Rtf2^{tm1a(KOMP)wtsi}$	KOMP	MGI code: 1913654

mESC long range genotyping primers		
**Oligonucleotide**	**VENDOR**	**IDENTIFIER**
mESCs Cassette 3' Universal Forward cacacctccccctgaacctgaaac	IDT	KOMP
mESCs Cassette 5' Universal Reverse ggtggtgtgggaaagggttcgaag	IDT	KOMP
mESCs Cassette GF3 gccgaagaaggtcgagaaggtcag	IDT	KOMP
mESCs Cassette GR3 cgaatctctccacctgctcaatccag	IDT	KOMP

RTF2 mouse genotyping primers		
**Oligonucleotide**	**VENDOR**	**IDENTIFIER**
Genotyping_PCR1_Fwd gcctgtgagcttggcaggta	IDT	This Paper
Genotyping_PCR1_Rev aggggaagacctgactgtgt	IDT	This Paper
Genotyping_PCR2_Fwd gcctgtgagcttggcaggta	IDT	This Paper

Genotyping_PCR2_Rev agcctgagctctgtcacatt	IDT	This Paper
Genotyping_PCR3_Fwd gatattgctgaagagcttgg	IDT	This Paper
Genotyping_PCR3_Rev gaagttatctcgacgaagttc	IDT	This Paper

RT-qPCR primers		
**Oligonucleotide**	**VENDOR**	**IDENTIFIER**
Mouse *Rtf2* RT-qPCR Forward gaagtgtgtcacacgtgtgg	IDT	This Paper
Mouse *Rtf2* RT-qPCR Reverse tttcttttccagcttggccc	IDT	This Paper
Human *RTF2* RT-qPCR Forward tgctgaagacaaggatggag	IDT	Kottemann et al
Human *RTF2* RT-qPCR Reverse tgaaacagactctgctgcct	IDT	Kottemann et al.
Mouse *Rnaseh2a* RT-qPCR Forward gcatctttgccaaggtggcc	IDT	This Paper
Mouse *Rnaseh2a* RT-qPCR Reverse ggtcttgggatcattgggat	IDT	This Paper
Human *RNASEH2A* RT-qPCR Forward gctgaaagtggcagactcaa	IDT	This Paper
Human *RNASEH2A* RT-qPCR Reverse caggttgtatttgacccgcc	IDT	This Paper
Human *RNASEH1* RT-qPCR Forward aggaatcggcgtttactggg	IDT	This Paper
Human *RNASEH1* RT-qPCR Reverse aggctgcatgaatttccgc	IDT	This Paper
Human *DDI1* RT-qPCR Forward tggaacacaacgtgctacct	IDT	Kottemann et al.
Human *DDI1* RT-qPCR Reverse atctgtctgggggctgtct	IDT	Kottemann et al.
Human *DDI2* RT-qPCR Forward cgatgtagtgtgtgtgtactgc	IDT	Kottemann et al.
Human *DDI2* RT-qPCR Reverse ccagtgaggtagattctttaccactt	IDT	Kottemann et al.
Human *GAPDH* RT-qPCR Forward ggtcggagtcaacggattt	IDT	This Paper
Human *GAPDH* RT-qPCR Reverse gccccacttgattttggag	IDT	This Paper
Mouse b-actin RT-qPCR Forward ctaaggccaaccgtgaaaag	IDT	Thongthip et al.
Mouse b-actin RT-qPCR Reverse accagaggcatacagggaca	IDT	Thongthip et al.
Human *PRIM1* Forward RT-qPCR gacagagcattgaaggagga	IDT	This Paper
Human *PRIM1* Reverse RT-qPCR cgtcttgaccaccctttaca	IDT	This Paper
Human *PRIMPOL* Forward RT-qPCR ggcacttcagtagaaaccat	IDT	This Paper
Human *PRIMPOL* Reverse RT-qPCR cgccgaattcctcctttaat	IDT	This Paper

Gateway cloning primers		
**Oligonucleotide**	**VENDOR**	**IDENTIFIER**
attB human RNASEH2A Forward ggggacaagtttgtacaaaaaagcaggcttcatggatctcagcgagctgga	IDT	This Paper
attB human RNASEH2A Reverse ggggaccactttgtacaagaaagctgggtctagaggctggttgctgact	IDT	This Paper
attB mouse RTF2 Forward ggggacaagtttgtacaaaaaagcaggcttcatgggttgcgacggaggcac	IDT	This Paper
attB mouse RTF2 Reverse ggggaccactttgtacaagaaagctgggttcagaagcagtaggatgtgt	IDT	This Paper
attB human PRIM1 Forward ggggacaagtttgtacaaaaaagcaggcttcatggagacgtttgaccccac	IDT	This Paper
attB human PRIM1 Reverse ggggaccactttgtacaagaaagctgggtttatttctcaaggaaaattt	IDT	This Paper

Mutagenesis cloning primers

Oligonucleotide	VENDOR	IDENTIFIER
human RNASEH2B_F300A;F301A Forward ccaattttttttttattttttaccccagcagcggtatcaatacttttcattccactcttgtcaactttagcca	IDT	This Paper
human RNASEH2B_F300A;F301A Reverse tggctaaagttgacaagagtggaatgaaaagtattgataccgctgctggggtaaaaaataaaaaaa aaattgg	IDT	This Paper
human RNASEH2A_D34A cctgggcgtcgctgaggcgggca	IDT	This Paper
human RNASEH2A_D169A caaggccaaagcagctgccctctacccgg	IDT	This Paper
human RNASEH2A_P40D cgggcaggggcgacgtgctgggcc	IDT	This Paper
human RNASEH2A_R210A actgattatggctcaggcgcccccaatgatcccaagac	IDT	This Paper

CRISPR cloning primers		
**Oligonucleotide**	**VENDOR**	**IDENTIFIER**
RTF2 5'BamHI aaaaggatcccatgggttgcgacgggggga	IDT	This Paper
RTF2 3'NotI aaaagcggccgctcagaagcagtaggacgtgtgg	IDT	This Paper
hRTF 5’ UTR Fwd INFUSION accatgattacgccaagcttactcttggaacgggcatggc	IDT	This Paper
hRTF 5’ UTR Rev INFUSION tcctcgcccttgctcaccatcgcaggacgggagtcagagc	IDT	This Paper
GFP Fwd INFUSION atggtgagcaagggcgagga	IDT	This Paper
hRTF rev INF gagcggtggcagtgcgggcttcagaagcagtaggacgtgt	IDT	This Paper
hRTF 3’ UTR Fwd INF agcccgcactgccaccgctc	IDT	This Paper
hRTF 3’ UTR Rev INF aacgacggccagtgaattctaacttataggcagataaaat	IDT	This Paper
mouse sgTrp53 exon 5 Fwd caccgaagtcacagcacatgacgg	IDT	This Paper
mouse sgTrp53 exon 5 Rev aaacccgtcatgtgctgtgacttc	IDT	This Paper

siRNAs and shRNAs		
**Oligonucleotide**	**VENDOR**	**IDENTIFIER**
Luciferase siRNA	Thermo Fischer	12935146
hRNASEH1 siRNA 1 gggaaagaggugaucaacatt	Ambion	s48356
hRNASEH1 siRNA 2 cagacaguauguuuacgautt	Ambion	s48357
hRNASEH1 siRNA 3 cgggauuuauaggcaauatt	Ambion	s48358
hRNASEH2A siRNA 1 caaugaucccaagacaaatt	Ambion	s20656
hRNASEH2A siRNA 2 ccaccgauauuuccuggaatt	Ambion	s20657
hRTF2 siRNA 1 caaagaugccgucauugaatt	Ambion	s226737
hPrim1 siRNA 1 gaaccagagauuauaagaatt	Ambion	s11050
hPrim1 siRNA 2 caucgucucugguauauutt	Ambion	s11051
hPrim1 siRNA 3 caacuacgguggagugauatt	Ambion	s10052
hPrimpol siRNA 1 ggcuauugauagaguuaaatt	Ambion	s11053

hPrimpol siRNA 2 ccacgaagaagagaucauatt	Ambion	s11054
hPrimpol siRNA 3 ggaucuccuucgauuuagatt	Ambion	s11055
hDDI1 siRNA1 ccggagacaucaauguuccaucgat	ThermoFisher	HSS181016
hDDI1 siRNA 2 ggaaauuacacauucagucauggat	ThermoFisher	HSS140552
hDDI1 siRNA 3 ccggagacaucaauguuccaucgat	ThermoFisher	HSS140553
hDDI2 shRNA uggaaaucgauacagcuca	Open Biosystems	V3LHS_328065
mRNASEH2A shRNA #1 ccgggctcgattacaacagcactttctcgagaaagtgctgttgtaatcgagctttttg	MilliporeSigma	TRC0000119585
mRNASEH2A shRNA #2 ccggcgggtcgttgtcgtctgagttctcgagaactcagacgacaacgacccgtttttg	MilliporeSigma	TRC0000119584

Plasmids		
**Plasmids**	**VENDOR**	**IDENTIFIER**
Gateway Entry Vectors		
pDONOR233; spectinomycin resistant; Gateway	Smogorzewska Lab	Smogorzewska Lab
pENTR-EV; spectinomycin resistant; Gateway	Smogorzewska Lab	Smogorzewska Lab
pENTR-HA-FLAG-mRTF2; spectinomycin resistant; Gateway	This paper	This paper
pENTR-RNASEH2A; spectinomycin resistant; Gateway	This paper	This paper
pENTR-RNASEH2A; spectinomycin resistant; Gateway	This paper	This paper
pENTR-RNASEH2A^{CD}_D34A;D169A; spectinomycin; Gateway	This paper	This paper
pENTR-RNASEH2A^{IOF}_P40D;Y210A; spectinomycin; Gateway	This paper	This paper
pENTR-RNASEH2B; spectinomycin resistant; Gateway	This paper	This paper
pENTR-RNASEH2B^{PIPm}_ F300A;F301A; spectinomycin resistant; Gateway	This paper	This paper
pENTR-hPRIM1; spectinomycin resistant; Gateway	This paper	This paper
Retroviral expression vectors		
PEA59-EV-dest (destination vector); chloramphenicol and ampicillin resistant; Gateway	Smogorzewska Lab	Smogorzewska Lab
PEA59-EV-puro; ampicillin resistant; retroviral; Gateway	Smogorzewska Lab	Smogorzewska Lab
PEA59-HA-FLAG-mRTF2-puro; retroviral; ampicillin resistant; Gateway	This paper	This paper
pMSCVpuro-DEST (destination vector); chloramphenicol and ampicillin resistant; Gateway	Addgene	Plasmid# 119745
pMSCVpuro-EV	This Paper	This Paper
pMSCVpuro-RNASEH2A	This Paper	This Paper
pMSCV_PM_shRNA_Control_puro	Smogorzewska Lab	Smogorzewska Lab
pMSCV_PM_shRNA_shDDI2_puro	Smogorzewska Lab	Smogorzewska Lab
pMSCV-GFP-H2B-hygro	Smogorzewska Lab	Smogorzewska Lab
pEGFP-RNASEH2B; kanamycin resistant; Gateway	Addgene	Plasmid #108697
MSCV_PM_shRNA_Control_puro	Smogorzewska Lab	Smogorzewska Lab
MSCV_PM_shRNA_shDDI2_puro	Smogorzewska Lab	Smogorzewska Lab
MSCV-GFP-H2B-hygro	Smogorzewska Lab	Smogorzewska Lab
pMMP Hit & Run Cre; retroviral; self-excising	Smogorzewska Lab	NA
pWZL Cre-hygro; retroviral	Smogorzewska Lab	NA
Lentiviral expression vectors		
CMV-HA-FLAG-Dest; destination vector; chloramphenicol and ampicillin resistant; Gateway	Smogorzewska Lab	Smogorzewska Lab

CMV-HA-FLAG-hPRIM1-puro; lentiviral; ampicillin resistant; Gateway	This paper	This paper
CMV-EV-puro; lentiviral; ampicillin resistant; Gateway	This paper	This paper
pLKO.1 shRNASEH2A #1_puro; ampicillin resistant	MilliporeSigma	SHCLNG-NM_027187, TRC0000119585
pLKO.1 shRNASEH2A #2_puro; ampicillin resistant	MilliporeSigma	SHCLNG-NM_027187, TRC0000119584
pLKO.1 shRNA Control Plasmid puro; ampicillin resistant;	MilliporeSigma	SHC002
pLVpuro-CMV-N-EGFP; destination vector; chloramphenicol and ampicillin resistant; Gateway	Addgene	Plasmid #122848
pLVpuro-CMV-N-EGFP- RNASEH2B; ampicillin resistant; Gateway	This paper	This paper
pLVpuro-CMV-N-EGFP- RNASEH2B^{PIPm}_F300A;F301A; ampicillin resistant; Gateway	This paper	This paper
Expression vectors		
ppyCAG_RNaseH1_WT; ampicillin resistant	Addgene	Plasmid #111906
ppyCAG_RNaseH1_D210N; ampicillin resistant	Addgene	Plasmid #11904
CRISPR generation of GFP-AID-RTF2 HEK 293Ts		
pcDNA5-FRT-TO-EGFP-AID	Addgene	Plasmid #80075
MLM3636	Addgene	Plasmid #43860
MLM3636 5' UTR_1 sequence acgctaggcgcggcgtagcg	This Paper	This Paper
px330	Addgene	Plasmid #42230
px330 3'UTR_1 atgtgaggcgtgtcggttcc	This Paper	This Paper
pUC19-5UTR-GFP-AID-hRTF2-3UTR Homology Donor Construct	This Paper	This Paper
CRISPR generation of p53-/-;Rtf2$^{+/lox}$ and p53-/-;Rtf2$^{-/lox}$ MEFs		
pSpCas9(BB)-2A-Puro (PX459) V2.0	Addgene	Plasmid #62988
pX459-sgTRP53	This Paper	This Paper
Protein Biochemistry		
PSKA002 HIS14-SUMO-MCS Expression Vector	Klinger Lab	Klinger Lab
PSKA002 HIS14-SUMO-RTF2	This paper	This paper
PSKA008 HIS14-GFP-MCS-Expression Vector	Klinger Lab	Klinger Lab
PSKA008 HIS14-GFP-RTF2	This paper	This paper
pGEX6P1-hsRNASEH2BCA	Addgene	Plasmid #108692
Packaging Constructs		
VSV-G; retroviral packaging	Smogorzewska Lab	Smogorzewska Lab
Gagpol; retroviral packaging	Smogorzewska Lab	Smogorzewska Lab
pMD2.G (VSV-G envelope expressing plasmid); lentiviral packaging	Addgene	Plasmid #12259
psPAX2; lentiviral packaging	Addgene	Plasmid #12260
Antibodies		
**ANTIBODY**	**SOURCE**	**IDENTIFIER**
Primary Antibodies		
Mouse IgG	Santa Cruz	Cat# sc-2025, RRID:AB_737182
Mouse monoclonal anti-alpha-tubulin (clone DM1A), WB:1:5000	MilliporeSigma	Cat# T9026, RRID:AB_477593

Mouse monoclonal anti-gammaH2AX Ser139 (clone JBW301), IF 1:2000	MilliporeSigma	Cat# 05-636, RRID:AB_309864
Mouse monoclonal anti-biotin, IF: 1:2000	Jackson ImmunoResearch	Cat# 200-002-211, RRID:AB_2339006
Mouse monoclonal anti-BrdU (B44), combing: 1:10	BD Biosciences	Cat# 347580, RRID:AB_400326
Mouse monoclonal anti-CHK1, WB:1:1000	MilliporeSigma	Cat # C9358, RRID:AB_259159
Mouse monoclonal anti-Poly (ADP-Ribose) Polymer antibody [10H], WB:1:100	Abcam	Cat # ab14459, RRID:AB_301239
Mouse monoclonal anti-PCNA (PC10), WB: 1:1000,	Santa Cruz	Cat# sc-56, RRID:AB_628110
Mouse monoclonal anti-RNASEH2A, WB:1:500	Santa Cruz	Cat# sc-515475
Mouse monoclonal anti-RTF2 (clone OTI1E8), WB: 1:1000	LS Bio	Cat# LS-C340588
Mouse Monoclonal anti-vinculin, Unconjugated, Clone hVIN-1	MilliporeSigma	Cat# V9131, RRID:AB_477629
Rabbit IgG	Santa Cruz	Cat# sc-2027, RRID:AB_737197
Rat monoclonal anti-BrdU [BU1/75 (ICR1)], combing: 1:20	Abcam	Cat# ab6326, RRID:AB_305426
Rabbit monoclonal anti-MCM7 (D10A11) XP, WB: 1:1000	Cell Signaling	Cat# 3735S, RRID:AB_2142705
Rabbit polyclonal anti-c20orf43 (RTF2), WB: 1:500	Novus	Cat# NBP2-30645
Rabbit polyclonal anti-GFP	Smogorzewska Lab	Kotteman et al.
Rabbit polyclonal anti-GFP	Abcam	Cat# ab290 RRIDL AB_303395
Rabbit polyclonal anti-RNASEH2A	Abcam	Cat# ab83943, RRID:AB_1861175
Rabbit polyclonal anti-RNASEH2C	AbClonal	Cat# A13884, RRID:AB_2760737
Rabbit polyclonal anti-phospho-CHK1 (S345), WB:1:1000	Cell Signaling Technology	Cat# 2341, RRID:AB_330023
Rabbit polyclonal anti-phospho-RPA32 (S4/8), WB:1:1000	Bethyl	Cat# A300-245A, RRID:AB_210547
Rabbit polyclonal anti-PRIM1	Proteintech	Cat # 10773-1-AP RRID:AB_2237549
Rabbit polyclonal anti-RPA32, WB 1:2000	Bethyl	Cat# A300-244A, RRID:AB_185548
Secondary Antibodies		
Goat Anti-Mouse IgG H&L Cross-Absorbed (Alexa Fluor® 488), IF:1:1000, combing:1:100	ThermoFisher	Cat# A-11029, RRID:AB_2534088
Goat Anti-Mouse IgG H&L Cross-Absorbed (Alexa Fluor® 647), combing:1:100	ThermoFisher	Cat# A-21235, RRID:AB_2535804
Goat Anti-Rabbit IgG H&L (Alexa Fluor® 488)	ThermoFisher	Cat# A-11008, RRID:AB_143165
Goat Anti-Rat IgG H&L Cross-Absorbed (Alexa Fluor® 594), IF:1:1000, combing:1:100	ThermoFisher	Cat# A-11007, RRID:AB_10561522
Peroxidase-AffiniPure Goat Anti-Mouse IgG (H + L) antibody, WB:1:2000	Jackson ImmunoResearch Labs	Cat# 115-035-003, RRID:AB_10015289
Peroxidase-AffiniPure Goat Anti-Rabbit IgG (H + L) antibody, WB:1:2000	Jackson ImmunoResearch Labs	Cat# 115-035-144,

Reagents		
**REAGENT/KIT**	**SOURCE**	**IDENTIFIER**
General Chemicals		
2-mercaptoethanol	MilliporeSigma	Cat# M3148
2-propanol	ThermoFisher	Cat# A416
3-Indoleacetic acid	MilliporeSigma	Cat# I3750-5G-A
5'-cholor-2'-deoxyuridine (CldU)	MilliporeSigma	Cat# C6891
5-ethynyl-2'-deoxyuridine (EdU)	ThermoFisher	Cat# A10044
5'-iodo-2'-deoxyuridine (IdU)	MilliporeSigma	Cat# I7125
Acetic Acid	ThermoFisher	Cat# A38
Acteone	ThermoFisher	Cat# A18
Agar, Bacto	VWR	Cat# 90000-762
Agarose, Low melt	Bio-Rad	Cat# 1613111
Agarose, SeaKem® LE	Lonza	Cat# 50004
Ammonium hydroxide	ThermoFisher	Cat# 15633520
Ampicillin	MilliporeSigma	Cat# A9518
Aphidicolin	MilliporeSigma	Cat# A0781
Blasticidin S HCl	ThermoFisher	Cat# R21001
Boric Acid	ThermoFisher	Cat# A73500
Bovine serum albumin	MilliporeSigma	Cat# A3059
Bromophenol Blue	Sigma	Cat# B8026
Chlorampenicol	MilliporeSigma	Cat# C0378
Chloroform	ThermoFisher	Cat# C298
Colcemid	MilliporeSigma	Cat# 234109
cOmplete™, EDTA-free Protease Inhibitor Cocktail	MilliporeSigma	Cat# 11873580001
DMSO	MilliporeSigma	Cat# D2650
EDTA	MilliporeSigma	Cat# E5134
EGTA	ThermoFisher	Cat# O2783
Ethanol, 200 proof, molecular grade	ThermoFisher	Cat# 22-032-601
Formaldehyde	ThermoFisher	Cat# F1635
Formamide	ThermoFisher	Cat# BP228100
FxCycle™ Violet Stain	ThermoFisher	Cat# F10347
Geneticin (Neomycin)	ThermoFisher	Cat# 10131035
Glycerol	ThermoFisher	Cat# G33500
H2O, molecular grade	VWR	Cat# 71002-726
HEPES	MilliporeSigma	Cat# H3375
Heptane	MilliporeSigma	Cat# 246654
Hydrochloric acid	ThermoFisher	Cat# A144S
Hydroxyurea	MilliporeSigma	Cat# H8627
Hygromycin B	ThermoFisher	Cat# 10687-010
Kanamycin	MilliporeSigma	Cat# K1876
MES monohydrate	MilliporeSigma	Cat# 69889
Methanol	ThermoFisher	Cat# A412
Mitomycin C	MilliporeSigma	Cat# M4287
MG-132	MilliporeSigma	Cat#474790
MgCl2	MilliporeSigma	Cat# M2670
N-ethylmaleimide	MilliporeSigma	Cat# E3876
N-Lauroylsarcosine sodium salt	MilliporeSigma	Cat# 61745

Phosphatase Inhibitor Cocktail I	MilliporeSigma	Cat# 524624
Phosphatase Inhibitor Cocktail II	MilliporeSigma	Cat# 524625
PIPES	MilliporeSigma	Cat# P6757
PMSF	MilliporeSigma	Cat# 10837091001
Polybrene (Hexadimethrine bromide)	MilliporeSigma	Cat# 28728-55-4
Potassium Chloride	ThermoFisher	Cat# P2173
Proteinase K	MilliporeSigma	Cat# 3115879001
Puromycin Dihydrochloride	MilliporeSigma	Cat# P8833
Spectinomycin	MilliporeSigma	Cat# S9007
Sodium Acetate	ThermoFisher	Cat# S209500
Sodium Azide	MilliporeSigma	Cat# S2002
Sodium Chloride	ThermoFisher	Cat# S271
Sodium Deoxycholate	MilliporeSigma	Cat# D6750
Sodium dodecyl sulfate	MilliporeSigma	Cat# L4390
Sodium hydroxide	ThermoFisher	Cat# S318500
Sodium Phosphate Dibasic Heptahydrate	ThermoFisher	Cat# BP331500
Sodium Phosphate Monobasic Monohydrate	ThermoFisher	Cat# S369
Sucrose	ThermoFisher	Cat# S53
Tris Base	ThermoFisher	Cat# BP152
Triton X-100	MilliporeSigma	Cat# T8787
Trypsin	ThermoFisher	Cat# 25200-056
Tryptone	ThermoFisher	Cat# BP1421
Tween 20	ThermoFisher	Cat# BP337
Yeast Extract	ThermoFisher	Cat# BP1422
Enzymes		
BamHI	NEB	Cat# R3136
Beta-agarase	NEB	Cat# M0392
Benzonase	MilliporeSigma	Cat# E1014
DNase	ThermoFisher	EN0531
NotI	NEB	Cat# M0288
Genotyping		
1 Kb Plus DNA Ladder	ThermoFisher	Cat# 10787018
DirectPCR Lysis Reagent (Mouse Tail)	Viagen Biotech	Cat #102-T
GoTaq® DNA polymerase master mix	Promega	Cat# M7833
Mammalian cell culture		
DMEM	ThermoFisher	Cat# 11965-092
DMEM/F12	ThermoFisher	Cat# 11330-032
Fetal Bovine Serum (FBS)	R&D Systems	Cat# S11150
GlutaMAX™	ThermoFisher	Cat# 35050061
McCoy's 5A	ThermoFisher	Cat# 16600082
MEM non-essential AA 100X)	ThermoFisher	Cat# 11140076
Penicillin-streptomycin	ThermoFisher	Cat# 15140122
RNA preparation, reverse transcription and RT-qPCR		
RNeasy Plus Mini Kit	Qiagen	Cat# 74134
Platinum SYBR Green SuperMix-UDG	ThermoFisher	Cat# 11733046
SuperScript™ III First-Strand Synthesis	ThermoFisher	Cat# 18080051
Platinum SYBR Green SuperMix-UDG	ThermoFisher	Cat# 11733046

Plasmid generation and mutagenesis		
Chemically competent DH5-a *E. coli*	Smogorzewska Lab	
Chemically competent Stlb3 *E. coli*	Smogorzewska Lab	
BP Clonase II Enzyme Mix	ThermoFisher	Cat# 11789020
GenElute HP Plasmid DNA Maxiprep Kit	MilliporeSigma	Cat# NA0310
LR Clonase II Enzyme Mix	ThermoFisher	Cat# 11791100
Multi-Site Directed Mutagenesis Kit	Agilent	Cat# 200514
QIAprep Spin Miniprep Kit	Qiagen	Cat# 27106
PBS (10x)	ThermoFisher	Cat# 20012050
Transductions		
TransIT-293	MirusBio LLC	Cat# MIR2700
Generation of GFP-AID-RTF2 endogenously tagged HEK293T		
In-Fusion® HD Cloning Plus	Clontech	Cat# 638917
Immunoblotting		
DC™ Protein Assay	Bio-Rad	Cat# 5000111
NuPAGE™ 4-12% Bis-Tris Midi Protein Gels, 20- or 26-well, w/adapators	ThermoFisher	Cat# WG1402A<br>Cat# WG1403A
NuPAGE™ 4-12% Bis-Tris Mini Protein Gels, 10-well	ThermoFisher	Cat# NP0321
NuPAGE™ MOPS SDS Running Buffer (20X)	ThermoFisher	Cat# NP0001
NuPAGE™ Transfer Buffer	ThermoFisher	Cat# NP0006
Western Lighting Plus-ECL, Enhanced Chemiluminescence Substrate	PerkinElmer	Cat# NEL104001
Immunofluorescence		
Biotin azide	ThermoFisher	Cat# B10184
(+)-sodium L-ascorbate	MilliporeSigma	Cat# A7631
Click-iT™ EdU Alexa Fluor™ 488 Imaging Kit	ThermoFisher	Cat# C10337
Copper (II) sulfate pentahydrate	ThermoFisher	Cat# C489
DAPI Fluoromount-G®	SouthernBiotech	Cat# 0100-20
DuoLink® In Situ PLA Anti-mouse MINUS probe	MilliporeSigma	Cat# DUO92004
DuoLink ®In Situ PLA Anti-rabbit PLUS probe	MilliporeSigma	Cat# DUO92002
DuoLink® In Situ Detection Reagents Orange	MilliporeSigma	Cat# DUO92007
DuoLink® In Situ Wash Buffers	MilliporeSigma	Cat# DUO82049
Micro Cover Glasses, Round, No. 1.5 12 mm	ThermoFisher	Cat# 1254581
Flow cytometry)		
Click-iT™ EdU Alexa Fluor™ 647 Flow Cytomtery Kit	ThermoFisher	Cat# C10419
DNA Combing		
Combicoverslips	GenomicVision	Cat# COV-002-RUO
Disposable Reservoirs	GenomicVision	Cat# RES-001
FiberPrep® (DNA Extraction Kit)	GenomicVision	Cat# EXTR-001
Fluoromount-G®	SouthernBiotech	Cat# 0100-01
Micro Cover Glasses, Square, No. 1.5, 22x22	ThermoFisher	Cat# 12-541-BP
Octenyltrichlorosilane, mixture of isomers	MilliporeSigma	Cat# 539279
Co-immunoprecipitations)		
Dynabeads™ M-270 Epoxy	ThermoFisher	Cat# 14301
Dynabeads™ MyOne™ Streptavidin C1	ThermoFisher	Cat# 65001
Dynabeads™ Protein A	ThermoFisher	Cat# 10001D
siRNA transfections		

Lipofectamine RNAiMAX Transfection Reagent	ThermoFisher	Cat# 13778-150
Metaphase spreads		
Gurr Buffer Tablets	ThermoFisher	Cat# 10582013
KaryoMAX^{TM} Giemsa Stain Solution	ThermoFisher	Cat# 10092013

Equipment		
**EQUIPMENT**	**SOURCE**	**IDENTIFIER**
General Lab Equipment		
Nanodrop 2000c Spectrophotometer	ThermoFisher, Smogorzewska Lab	ND-2000C
PowerPac^{TM} Basic Power Supply	Bio-Rad, Smogorzewska Lab	Cat# 1645050
Thermocycler	Bio-Rad, Smogorzewska Lab	C1000
Wide Mini ReadySub-Cell GT Cell	Bio-Rad, Smogorzewska Lab	Cat# 1704489
Mammalian cell culture/ Growth and sensitivity assays		
Z2^{TM} Coulter Counter® Analyzer	Beckman Coulter, Smogorzewska Lab	Cat# 6605700
RNA preparation, reverse transcription and RT-qPCR		
Applied Biosystems™ QuantStudio™ 12K Flex	ThermoFisher, Rockefeller Genomics Resource Center	Cat# 4471087
Immunoblotting		
Azure c300 Chemiluminescent Western Blot Imaging System	Azure Biosystems, Smogorzewska Lab	Cat# c300
Criterion^{TM} Vertical Electrophoresis Cell	Bio-Rad, Smogorzewska Lab	Cat# 1656001
ImageQuantLAS 4000	GE Healthcare Life Science, Nussenzweig Lab	Cat# 28955810
XCell SureLock^{TM} Mini-Cell	ThermoFisher, Smogorzewska Lab	Cat#EI0001
Immunofluorescence		
Axio Observer.A1 fluorescence microscope, equipped with a Pan-Apochromat 63X NA-1.4 oil objective and Axio CCD camera	Carl Zeiss, Smogorzewska Lab	NA
Flow cytometry		
BD Accuri^{TM} C6	Becton Dickinson, Rockefeller Flow Cytometry Resource Center	NA
BD FACSAria^{TM} II	Becton Dickinson, Rockefeller Flow Cytometry Resource Center	NA
BD^{TM} LSR II	Becton Dickinson, Rockefeller Flow Cytometry Resource Center	NA
DNA Combing		
Branson 2800 liquid sonicator	ThermoFisher, Smogorzewska Lab	Cat# 15-336-126
FiberComb® (Molecular Combing System)	GenomicVision, Smogorzewska Lab	Cat# MCS-001
Gatan Model 950 Advanced Plasma System	Gatan, Rockefeller Cyro-EM Resource Center	

Inverted Olympus IX-71 DeltaVision Image Restoration Microscope, equipped with 60x oil objective and pco.edge sCOS camera	Applied Precision, Rockefeller Bioimaging Resource Center	NA
RNA sequencing		
NextSeq 500	Illumina, Rockefeller Genomics Resource Center	NA
Live Cell Imaging		
Olympus CellVoyager CV1000	Olympus, Rockefeller Bioimaging Resource Center	NA
Metaphase Spreads		
Humidifier	Smogorzewska Lab	NA
Metafer Slide Scanning Platform	MetaSystem, Smogorzewska Lab	NA

Supplementary References

1 Benitez-Guijarro, M. *et al.* RNase H2, mutated in Aicardi-Goutieres syndrome, promotes LINE-1 retrotransposition. *The EMBO journal* **37**, doi:10.15252/embj.201798506 (2018).

2 Cong, L. *et al.* Multiplex genome engineering using CRISPR/Cas systems. *Science (New York, N.Y.)* **339**, 819-823, doi:10.1126/science.1231143 (2013).
