# Supplementary material for: RTF2 controls replication repriming and ribonucleotide excision at the replisome
Source: Nat Commun. 2024 Mar 2;15:1943. doi: 10.1038/s41467-024-45947-z (PMC10908796; doi:10.1038/s41467-024-45947-z)
Supplement: Supplementary file 3 — Description of Additional Supplementary Files [file 41467_2024_45947_MOESM3_ESM.pdf]

Movies
All movies show representative mitoses in SV40-LT-immortalized *Rtf2*$^{-/-}$ MEFs.

Supplementary Movie 1. Normal mitosis.
Supplementary Movie 2. Failed mitosis generating anaphase bridged and lagging chromosomes.
Supplementary Movie 3. Another failed mitosis whereby the cell does not divide and instead forms a 'donut.'

Supplementary Data 1. Proteomic data from the iPOND experiment shown in Figure 3 (excel file)
Supplementary Data 2. Proteomic data from the IP experiments from HEK293T cells expressing endogenously GFP- tagged RTF2 shown in Supplementary Figure 6f (excel file)
Supplementary Data 3. Proteomic data from the IP experiments from HEK293T cells expressing exogenously GFP- tagged RTF2 shown in Supplementary Figure 6h (excel file)

Supplementary Table 1. List of reagents and equipment

Supplementary Figure Legends

Supplementary Figure 1. RTF2 is necessary for *in vivo* viability.
a, Schematic of crosses to generate the genetic knockout of RTF2 in *Mus musculus*. Mouse embryonic stem cells (mESCs) containing an *Rtf2*$^{tm1a(KOMP)Wtsi}$ *(Rtf2*^{stop}*)* allele (Knockout Mouse Project [KOMP]) were injected into mouse blastocysts (B6(Cg)-Tyr^{c-2J}/J, Jackson Labs) to create chimeric mice, which through appropriate crosses generated *Rtf2*$^{+/stop}$ mice. *Rtf2*$^{+/stop}$ mice were bred with mice expressing Flp recombinase and subsequently mice expressing Cre recombinase under a ubiquitous promoter. These crosses induced the loss of exon 3 to yield *Rtf2*$^{+/\Delta\, exon3}$ pups. The loss of exon 3 will herein be referred to as *Rtf2*$^{-}$. Mice with different *Rtf2* genotypes were maintained on C57BL/6 background. PCR primers and product sizes are indicated on the *Rtf2* alleles. **b**, Schematic of WT RTF2 protein. Loss of exon 3 results in early truncation of RTF2. **c**, Representative genotyping PCR products run on 0.8% agarose gel and stained with ethidium bromide. Long range PCR products amplified from the KOMP mESCs confirmed the presence of the FRT and LoxP sites after excision and analysis with Sanger sequencing. PCR1, PCR2, and PCR3 products amplified from DNA extracted from mouse tail tips were used to confirm genotypes. **d,** Genotypes from litters of *Rtf2*$^{+/stop}$ female mice crossed with *Rtf2*$^{+/stop}$ male mice. *Rtf2*$^{stop/stop}$ mice showing embryonic lethality. **e,** Weights from *Rtf2*$^{+/-}$ and *Rtf2*$^{+/+}$ mice. For each point, n$\geq$2 mice. Total n for the indicated genotypes were: 15 for female *Rtf2*$^{+/+}$, 21 for female *Rtf2*$^{+/-}$, 14 for male *Rtf2*$^{+/}$, and 34 for male *Rtf2*$^{+/-}$. In d. Chi-squared test statistic (X^2) and p-values are indicated for pups. In e, error bars indicate standard deviation. WT = wildtype. Source data are provided as a Source Data file.

Supplementary Figure 2. RTF2-deficient SV40-immortalized MEFs and clones accumulate aberrant nuclear structures that are suppressed by CDK1 inhibition. a,b,c, Representative RT-qPCR of relative *Rtf2* mRNA transcript levels in primary MEFs (a) or SV40-immortalized (b) MEFs at 96 hr after Cre, or from an SV40-immortalized MEF *Rtf2*$^{-/-}$ clone expressing empty vector (EV) or RTF2 cDNA (c). *Rtf2* expression was normalized to *β-actin* expression. **d,** Representative cell cycle profiles from flow cytometry of indicated SV40-immortalized MEFs at 72 hr after Cre. **e,** Average percentage of G1, S phase, and G2 cells from (d). **f,** Representative immunoblot of whole cell lysates in SV40-immortalized RTF2-deficient sub-cloned MEF lines expressing empty vector (EV) or HA-FLAG-mRTF2 (RTF2) cDNA constructs. α-tubulin represents loading control. **g,** Representative growth curves of SV40-immortalized RTF2-deficient sub-cloned MEF lines expressing EV or HA-FLAG-mRTF2. **h**, Representative images of DAPI staining from indicated SV40-immortalized MEFs expressing EV or HA-FLAG-mRTF2 at 120 hr after Cre. Arrows indicate abnormal nuclei. **i,** Quantification of percentage of cells with abnormal nuclei based on DAPI staining from (h). **j**, Representative images of GFP-H2B staining in live *Rtf2*$^{-/-}$ SV40-immortalized MEFs expressing empty vector (EV) and transduced with pWZL Cre-hygro retrovirus 120 hrs prior to analysis. In row 1, a cell enters mitosis and undergoes a normal division. In rows 2-4, cells fail to complete successful mitosis, resulting in lagging

chromosomes and/or abnormal nuclear morphology. **k,** Quantification of abnormal mitoses from live-cell imaging of cells in (j). **l,** Representative images of DAPI-stained nuclei from SV40-immortalized MEFs at 120 hr after Cre and then treated with CDK1 inhibitor (RO-3306) for an additional 24 hr. Arrows indicate abnormal nuclei. **m,** Quantification of abnormal nuclei in images shown in (l). Experiments were conducted at least three times in biological replicates with technical triplicates for a-c. Representative plot of normalized average across technical triplicates is shown, with error bars indicating standard deviation, for a-c. Experiments were conducted at least three times in biological replicates with consistent results for d-i,l,m. Averages from two biological replicates plotted in e, with error bars representing standard deviation. EV = empty vector. Source data are provided as a Source Data file.

Supplementary Figure 3. RTF2-deficient replication forks progress symmetrically from origins.
a, Top: Schematic of replication initiation sites, identified as species where the second label (CldU) flanks the first label (IdU). Left fork length is plotted against right fork length to determine fork symmetry. Bottom: Representative experiment showing fork symmetry from primary MEFs 72 hrs after transduction with Cre. Left fork length is plotted against right fork length. Lines represent arbitrary cutoffs for replication forks with symmetry less than 2 and greater than 0.5. Percentages in the bottom right corner represent the percentage of asymmetric forks. Source data are provided as a Source Data file.

Supplementary Figure 4. RTF2-deficient MEFs do not display changes in global intron retention or gene expression.
a, Schematic of RTF2 from *Mus musculus, Homo sapiens* and *Arabidopsis thaliana*. Amino acids 7-63 in atRTF2 are implicated in an intron-retention defect in plants. **b,** Percent of reads from paired-end RNA-seq mapping to introns. Genotypes are indicated for SV40-immortalized MEFs expressing cDNA for HA-FLAG-empty-vector (EV) or HA-FLAG-mouse-RTF2 (RTF2) 120 hrs after transduction with pWZL Cre-hygro retrovirus **c,** Volcano plot showing $\log_{10}$p-values against $\log_2$fold change for the significant differentially expressed genes as calculated by DESeq2. Genes with $\log_2$fold change >1 and $p_{adjusted}$-value <0.05 averaged across the two biological replicates (technical triplicate). These results indicate 57 genes significantly downregulated and 7 genes significantly upregulated. n=3 for each set of MEFs, technical replicate. Comparison is between aligned single-end reads from *Rtf2*$^{+/+}$ and *Rtf2*$^{-/lox}$ SV40-immortalized MEFs transduced with Hit & Run pMMP Cre retrovirus 72 hrs prior to harvest. Source data are provided as a Source Data file.

Supplementary Figure 5. RTF2 deficiency results in loss of RNase H2 from the replication fork.
a, Quantification of EdU-EdU PLA foci and single antibody (RNASEH2A and EdU) controls for nPLA in Fig. 3d. **b,** Representative immunoblot of whole cell lysates showing RNASEH2A and RNASEH2C levels in primary MEFs transduced with Hit & Run Cre recombinase retrovirus 72 hrs before harvest. **c,d,** RT-qPCR analysis of relative mouse *Rnaseh2a* and *Rtf2* transcript levels in primary MEFs transduced with Hit & Run pMMP Cre retrovirus 72 hrs before harvest or in SV40-LT immortalized RTF2-deficient sub-cloned MEF lines expressing HA-FLAG empty vector (EV) or mRTF2 (RTF2) cDNA constructs, respectively. Expression is normalized to *β-actin*. **e,** Quantification of GFP (RNASEH2B)-EdU PLA foci in untreated cells (no EdU) for nPLA in Fig. 4a. **f,** Quantification of EdU-EdU PLA foci for nPLA in Fig. 4a. Experiments were conducted at least three times in biological replicates with consistent results for a,b. Experiment conducted twice in biological replicates with consistent results for e,f. Experiments were conducted at least three times in biological replicates with technical triplicates for c,d. Representative plot of normalized average across technical triplicates is shown, with error bars indicating standard deviation, for c.d. Mean is indicated with a red line for a,e,f. Significance evaluated by Kruskal-Wallis ANOVA with a Dunn's post-test. EV = empty vector, RH2A = RNASEH2A, RH2C = RNASEH2C, WT = wildtype, PIPm = PIP box mutant. Source data are provided as a Source Data file.

Supplementary Figure 6. RTF2 interacts with RNase H2 and components of the replisome.
a, Schematic of CRISPR-Cas9 targeting to generate a tagged RTF2 construct expressed from the endogenous *RTF2* locus. A plasmid carrying a GFP-AID-h*RTF2* cDNA flanked by homology arms to the 5'UTR and 3'UTRs (orange boxes) of *RTF2* was targeted to the endogenous locus of Rtf2 in HEK293T cells and subsequently cloned. This line will be referred to as endogenous GFP-AID-RTF2 HEK293Ts. **b,** Genotyping analysis of wild type and endogenous GFP-AID-*RTF2* HEK293Ts. Schematic represents genotyping primers that were used to amplify the endogenously tagged locus. Forward primer recognizes *RTF2* promoter region upstream to the GFP-AID-h*RTF2* insert and reverse

primer recognizes an *RTF2* exonic region. The primer pair amplifies a wild type *RTF2* allele of 1124 bp and the GFP-tagged allele of 2571 bp. **c,** Flow cytometry analysis of wild type and endogenous GFP-AID-RTF2 HEK293Ts. **d,** Representative images of immunofluorescence analysis of endogenous GFP-AID-RTF2 HEK293Ts. Cells were fixed and stained with anti-GFP antibodies. **e,** Representative immunoprecipitation with GFP antibodies from wild type and endogenous GFP-AID-RTF2 HEK293Ts. Immunoblotted with RTF2 antibody. The GFP-AID-h*RTF2* protein is predicted to be 90.5 kDa. **f,** GFP was immunoprecipitated with GFP antibodies from wild type and endogenously targeted GFP-AID-RTF2 HEK293Ts described in a-e. Average peptide spectral matches (#PSM) and area under the curve (AUC) from LC-MS for given proteins averaged across two biological replicates. **g,** Representative immunoprecipitation with GFP from HEK293Ts retrovirally expressing GFP-Empty Vector (GFP-EV) or GFP-human-RTF2 (GFP-RTF2). The GFP-h*RTF2* construct is predicted to be 63.8 kDa. **h,** GFP was immunoprecipitated from the chromatin fraction of cell lysates with GFP nanobodies isolated from cells in g. Average #PSM and AUC from LC-MS for given proteins averaged across two biological replicates **i,** Representative immunoblot from immunoprecipitation of recombinant RNase H2 complex and RTF2 expressed in *E. coli*. Protein amount (pmol) are indicated above each lane; range of RTF2 is 0.5, 1, 2, 4 pmol. The blot corresponds to immunoblot shown in Fig. 4e. Experiments were conducted at least two times in biological replicates with consistent results for f,h,i. EV = empty vector, RH2A = RNASEH2A, RH2B = RNASEH2B, RH2C = RNASEH2C. Source data are provided as a Source Data file.

Supplementary Figure 7. RNase H2-deficient cells phenocopy growth and replication phenotypes of RTF2 deficiency.

a, Representative immunoblot showing loss of RNASEH2A in CRISPR-edited HCT116 *p53*$^{-/-}$ cells. α-tubulin represents loading control. **b,** Growth curves of indicated cells. **c,** Quantification of representative experiment showing mean signal of EdU in EdU-positive cells in indicated cells. **d,** Representative RT-qPCR analysis of relative human *RNASEH2A* transcript levels in BJ cells. Expression is normalized to *β-actin* expression. **e,** Mean nuclear signal of EdU in EdU-positive BJ cells treated with indicated siRNAs. **f,** Growth curves of HeLa cells and CRISPR-edited RNASEH2A KO HeLa cells complemented with wildtype, catalytic dead (RH2A^{CD}/RNASEH2A$^{D34A;D169A}$), or separation of function (RH2A^{SOF}/RNASEH2A$^{P40D;Y210A}$) RNASEH2A. **g,** Growth curves of HeLa cells and CRISPR-edited RNASEH2A KO HeLa cells expressing empty vector (EV), wildtype V5-RNASEH1 (RH1), or catalytic dead (RH1^{CD}/V5-RNASEH1^{D210N}) RNASEH1. **h,** Representative RT-qPCR analysis of mouse *Rnaseh2* expression in SV40-immortalized *Rtf2*$^{-/-}$ MEF clones expressing empty vector (EV) or HA-FLAG-mRTF2 (RTF2) transduced with indicated shRNAs. *Rnaseh2* expression is normalized to *b-actin*. Experiments were conducted at least three times in biological replicates with consistent results for a,c,e. Experiments were conducted at least three times in biological replicates with technical triplicates for d,h. Representative plot of normalized average across technical triplicates is shown, with error bars indicating standard deviation, for d,h. Experiments were conducted three times in biological replicates with technical triplicates for b,f,g. Error bars represent standard deviation. Each dot represents one cell for c,e. Mean for each sample shown with red line for c,e. Cells were pulsed with EdU for 1 hr prior to fixation for c,e, Experiments were blinded prior to analysis for c,e. Significance evaluated by Kruskal-Wallis ANOVA with a Dunn's post-test. RH2A = RNASEH2A, P = Parental, WT = wildtype, EV = empty vector, RH2A^{CD} = catalytic dead RNASEH2A$^{D34A/D169A}$, RH2A^{SOF} = separation of function RNASEH2A$^{P40D/Y210A}$, RH1 = RNASEH1, CD = catalytic dead RNASEH1^{D210N}, Ctrl = Control, NT = Non-targeting. Source data are provided as a Source Data file.

Supplementary Figure 8. Removal of RNase H2 from stalled replication forks allows for replication restart and genome stability.

a, Representative immunoblot in U2OS cells transduced or transfected with indicated RNAi reagents and treated with HU (0 = untreated, 24 = 24 hr treatment, R = 24 hr treatment followed by 8 hr release). **b,** Quantification of representative experiment of the percentage of metaphase spreads in each category described in Fig. 7c in U2OS cells transduced or transfected with indicated RNAi reagents (112 hrs). n>50 for metaphases scored in each sample. **c,** Schematic and representative images of DNA combing replication fork restart assay. PBS washes are indicated by a black vertical line in all schematics. **d,** Top: Labeling schematic. Bottom: Ratio of CldU tract to IdU tract lengths in U2OS cells transfected with indicated siRNAs for 72 hrs. **e,f,** RT-qPCR analysis of *RNASEH2A* or *RNASEH1* expression in indicated cells normalized to *GAPDH*. **g, h, i,** Top: Labeling schematic. Mean nuclear signal of EdU in EdU-positive U2OS cells treated as indicated. Experiments conducted at least three times in biological replicates with consistent results for a,b,d-i. Error bars represent standard deviation. Experiments were blinded prior to analysis for d. Average CldU:IdU ratios are listed above each sample for d, g-i. Outliers removed with ROUT (1%) for d. Each dot represents one cell for g-i. Mean shown with red line for d, g-i. Experiments were conducted at least three times

in biological replicates with technical triplicates for h,i. Representative plot of normalized average across technical triplicates is shown, with error bars indicating standard deviation, for e,f. Significance evaluated by Kruskal-Wallis ANOVA with a Dunn's post-test. RH2A= RNASEH2A, Ctrl = Control, Luc = Luciferase, RH2A = RNASEH2A, Aphi = Aphidicolin, CPT = Camptothecin, RH1 = RNASEH1. Source data are provided as a Source Data file.

Supplementary Figure 9. Catalytic activity of primase PRIM1, not PRIMPOL, is required for efficient replication restart after stress.
a-c, Representative RT-qPCR analysis of relative human *RNASEH2A, RTF2,* or *PRIMPOL* transcript levels in indicated U2OS cells. Expression is normalized to *GAPDH.* **d-f,** Representative RT-qPCR analysis of relative human *RNASEH2A, RTF2,* or *PRIM1* transcript levels in indicated U2OS cells. Expression is normalized to *GAPDH.* **g,h,** Top: Labeling schematics for DNA combing restart and progression assays in the setting of varying concentrations of V-TP, a potent PRIM1 inhibitor. PBS washes are indicated by a black vertical line in all schematics. Bottom: Quantification of representative experiment of CldU:IdU tract length ratios in U2OS cells transduced or transfected with indicated RNAi reagents. Experiments were conducted at least three times in biological replicates with technical triplicates for a-f. Representative plot of normalized average across technical triplicates is shown, with error bars indicating standard deviation, for a-f. Experiments were conducted at least three times in biological replicates with consistent results for g,h. Experiments were blinded prior to analysis for g,h. Mean is shown with red line for g,h. Average CldU:IdU ratios are listed above each sample for g,h. Outliers removed with ROUT (1%) for g,h. Significance evaluated by Kruskal-Wallis ANOVA with a Dunn's post-test. RH2A= RNASEH2A, Ctrl = Control. Source data are provided as a Source Data file.

Supplementary Figure 10. Replication restart is sensitive to cellular levels of PRIM1.
a, IdU lengths in indicated RPE cells from experiment in Fig. 9b. Mean for each sample shown with red line. Average length indicated above each sample. **b,** Top: nPLA labeling schematic. PBS washes are indicated by a black vertical line in all schematics. Bottom: Quantification of PRIM1(GFP)-EdU PLA foci in PRIM1-AID-mCLOVER RPE cells in the setting of progressing or stalled replication. **c,** Representative images of PRIM1(GFP)-EdU foci in PRIM1-AID -mCLOVER RPE cells. DAPI stains the nucleus. **d,** Quantification of single antibody (PRIM1 (GFP) and EdU) and EdU-EdU PLA foci controls for nPLA in Supplementary Fig. 10b. **e,** Top: Labeling schematic for DNA combing. PBS washes are indicated by a black vertical line in all schematics. Below: Quantification of representative experiment of CldU:IdU tract length ratios in U2OS cells transduced with either HA-FLAG-PRIM1 or HA-FLAG-EV and transduced or transfected with indicated RNAi reagents related to Fig. 9e. Experiments were conducted at least three times in biological replicates with consistent results for a-e. Mean shown with red line for a,b,d,e. Experiments were blinded prior to analysis for a ,e. Average CldU:IdU ratios or tract length are listed above each sample for a,e. Outliers removed with ROUT (1%) for a,e. Significance evaluated by Kruskal-Wallis ANOVA with a Dunn's post-test. Ctrl = Control, RH2A = RNASEH2A, IAA = auxin, EV = empty vector. Source data are provided as a Source Data file.
