# Supplementary material for: RTF2 controls replication repriming and ribonucleotide excision at the replisome
Source: Nat Commun. 2024 Mar 2;15:1943. doi: 10.1038/s41467-024-45947-z (PMC10908796; doi:10.1038/s41467-024-45947-z)
Supplement: Supplementary file 10 — Reporting Summary [file 41467_2024_45947_MOESM10_ESM.pdf]

nature portfolio

Corresponding author(s): Agata Smogorzewska

Last updated by author(s): 2/3/2024

Reporting Summary

Nature Portfolio wishes to improve the reproducibility of the work that we publish. This form provides structure for consistency and transparency in reporting. For further information on Nature Portfolio policies, see our Editorial Policies and the Editorial Policy Checklist.

Statistics

For all statistical analyses, confirm that the following items are present in the figure legend, table legend, main text, or Methods section.

n/a	Confirmed	
☐	☒	The exact sample size (*n*) for each experimental group/condition, given as a discrete number and unit of measurement
☐	☒	A statement on whether measurements were taken from distinct samples or whether the same sample was measured repeatedly
☐	☒	The statistical test(s) used AND whether they are one- or two-sided<br>*Only common tests should be described solely by name; describe more complex techniques in the Methods section.*
☒	☐	A description of all covariates tested
☐	☒	A description of any assumptions or corrections, such as tests of normality and adjustment for multiple comparisons
☐	☒	A full description of the statistical parameters including central tendency (e.g. means) or other basic estimates (e.g. regression coefficient) AND variation (e.g. standard deviation) or associated estimates of uncertainty (e.g. confidence intervals)
☐	☒	For null hypothesis testing, the test statistic (e.g. *F*, *t*, *r*) with confidence intervals, effect sizes, degrees of freedom and *P* value noted<br>*Give P values as exact values whenever suitable.*
☒	☐	For Bayesian analysis, information on the choice of priors and Markov chain Monte Carlo settings
☒	☐	For hierarchical and complex designs, identification of the appropriate level for tests and full reporting of outcomes
☒	☐	Estimates of effect sizes (e.g. Cohen's *d*, Pearson's *r*), indicating how they were calculated

Our web collection on statistics for biologists contains articles on many of the points above.

Software and code

Policy information about availability of computer code

Data collection	Primary data was collected using Microsoft Excel.
Data analysis	Immunofluorescence images were anlalyzed using FIJI (version 2.11.0) or CellProfiler (version 4.1.3), as indicated in Methods. GraphPad Prism (version 9.5.1) was used for all statistical analyses. Transcript expressions were calculated using the Salmon quantification software (version 0.8.2) and gene expression levels as TPMs and counts retrieved using Tximport (version 1.8.0). Normalization and rlog transformation of raw read counts in genes were performed using DESeq2 (version 1.20.0). For visualisation in genome browsers, RNA-seq reads are aligned to the genome using Rsubread's subjunc method (version 1.30.6) and exported as bigWigs normalised to reads per million using the rtracklayer package (version 1.40.6).For intron retention analysis, raw reads were aligned to the mouse genome (mm10) with Strand NGS (version 2.1) .

For manuscripts utilizing custom algorithms or software that are central to the research but not yet described in published literature, software must be made available to editors and reviewers. We strongly encourage code deposition in a community repository (e.g. GitHub). See the Nature Portfolio guidelines for submitting code & software for further information.

Data

Policy information about availability of data

All manuscripts must include a data availability statement. This statement should provide the following information, where applicable:

- Accession codes, unique identifiers, or web links for publicly available datasets
- A description of any restrictions on data availability
- For clinical datasets or third party data, please ensure that the statement adheres to our policy

Mouse RNA sequencing data have been deposited in NCBI's Gene Expression Omnibus and are accessible through GEO Series accession number GSE152047 [https://www.ncbi.nlm.nih.gov/geo/query/acc.cgi?acc=GSE152047].
Sequence and transcript coordinates for mouse mm10 UCSC genome and gene models are available from the Bioconductor Bsgenome.Mmusculus.UCSC.mm10 (https://bioconductor.org/packages/release/data/annotation/html/BSgenome.Mmusculus.UCSC.mm10.html) and TxDb.Mmusculus.UCSC.mm10.knownGene https://bioconductor.org/packages/release/data/annotation/html/TxDb.Mmusculus.UCSC.mm10.knownGene.html Bioconductor libraries respectively. Source data are provided with this paper. Raw data from the proteomic studies are not available, but the complete primary data is included in Supplementary tables 1-3.

Research involving human participants, their data, or biological material

Policy information about studies with human participants or human data. See also policy information about sex, gender (identity/presentation), and sexual orientation and race, ethnicity and racism.

Reporting on sex and gender	N/A
Reporting on race, ethnicity, or other socially relevant groupings	N/A
Population characteristics	N/A
Recruitment	N/A
Ethics oversight	N/A

Note that full information on the approval of the study protocol must also be provided in the manuscript.

Field-specific reporting

Please select the one below that is the best fit for your research. If you are not sure, read the appropriate sections before making your selection.

☒ Life sciences ☐ Behavioural & social sciences ☐ Ecological, evolutionary & environmental sciences

For a reference copy of the document with all sections, see nature.com/documents/nr-reporting-summary-flat.pdf

Life sciences study design

All studies must disclose on these points even when the disclosure is negative.

Sample size	Sample size was set according to standards of the field (> 150 fibers/ replicate for combing; > 50 cells for cell-based assays). The specific sample size (number of nuclei or number of DNA combing tracts scored per experiment) is indicated.v
Data exclusions	Outliers were removed from DNA combing experiments to account for variability in the assay, and the experiments that have had outliers removed are indicated in the figure legends. To remove outliers, 1% ROUT analysis was performed in GraphPad Prism.
Replication	The number of independent experiments performed per result is indicated in the figure legend. There were no experiments included in which the results were not reproducible in our hands.
Randomization	The experiments in this study rely largely on immunofluorescence, immunoblot, and DNA combing approaches. Samples cannot be randomized, given the different treatments done for each sample prior to fixation and assay readout (such as siRNA transfection, Cre transduction, etc). To account for potential bias in scoring, the imaging-based experiments were blinded prior to image acquisition and analysis.
Blinding	Immunoluorescence and DNA combing experiments were blinded prior to imaging and analysis by numbering the coverslips. Data was unblinded after collection and analysis.

Reporting for specific materials, systems and methods

We require information from authors about some types of materials, experimental systems and methods used in many studies. Here, indicate whether each material, system or method listed is relevant to your study. If you are not sure if a list item applies to your research, read the appropriate section before selecting a response.

Materials & experimental systems

n/a	Involved in the study
☐	☒ Antibodies
☐	☒ Eukaryotic cell lines
☒	☐ Palaeontology and archaeology
☐	☒ Animals and other organisms
☒	☐ Clinical data
☒	☐ Dual use research of concern
☒	☐ Plants

Methods

n/a	Involved in the study
☒	☐ ChIP-seq
☐	☒ Flow cytometry
☒	☐ MRI-based neuroimaging

Antibodies

Antibodies used

Mouse IgG Santa Cruz Cat# sc-2025, RRID:AB_737182 D2022
Rabbit IgG Santa Cruz Cat# sc-2027, RRID:AB_737197
Rabbit IgG Cell Signaling Technology Cat# 2729, RRID:AB_1031062 10
Mouse monoclonal anti-�-tubulin (clone DM1A), WB:1:5000 MilliporeSigma Cat# T9026, RRID:AB_477593 0000137585
Mouse monoclonal anti-�H2AX Ser139 (clone JBW301), IF 1:2000 MilliporeSigma Cat# 05-636, RRID:AB_309864 3782118
Mouse monoclonal anti-biotin, IF: 1:2000 Jackson ImmunoResearch Cat# 200-002-211, RRID:AB_2339006 151728
Rabbit monoclonal anti-biotin, IF: 1:2000 Bethyl A150-109A 11
Mouse monoclonal anti-BrdU (B44), combing: 1:10 BD Biosciences Cat# 347580, RRID:AB_400326 9172603
Mouse monoclonal anti-Poly (ADP-Ribose) Polymer antibody [10H], WB:1:100 Abcam Cat # ab14459, RRID:AB_301239
Mouse monoclonal anti-PCNA (PC10), WB: 1:1000 Santa Cruz Cat# sc-56, RRID:AB_628110 K1121
Mouse Monoclonal anti-vinculin, Unconjugated, Clone hVIN-1 MilliporeSigma Cat# V9131, RRID:AB_477629 018M4779V
Rat monoclonal anti-BrdU [BU1/75 (ICR1)], combing: 1:20 Abcam Cat# ab6326, RRID:AB_305426
Rabbit monoclonal anti-MCM7 (D10A11) XP, WB: 1:1000 Cell Signaling Technology Cat# 3735S, RRID:AB_2142705 3
Rabbit polyclonal anti-c20orf43 (RTF2), WB: 1:500 Novus Cat# NBP2-30645 R72074
Rabbit polyclonal anti-RTF2, WB: 1:500 Proteintech Cat# 16633-1-AP, RRID: AB_2256547 00074586
Mouse monoclonal anti-RTF2 (clone OTI1E8), WB: 1:1000 LS Bio Cat# LS-C340588 75213
Rabbit polyclonal anti-GFP Smogorzewska Lab Kotteman et al.
Rabbit polyclonal anti-GFP Abcam Cat# ab290
RRIDL AB_303395 GR3251545-1
Mouse monoclonal Anti-GFP, WB: 1:3000 Roche Cat# 11814460001
Rabbit polyclonal anti-RNASEH2A, WB: 1:500 Proteintech Cat# 16132-1-AP, RRID:AB_2269729 00023264
Rabbit polyclonal anti-RNASEH2A, WB: 1:500 Abcam Cat# ab83943, RRID:AB_1861175 GR3212381-12
Mouse monoclonal anti-RNASEH2A, WB:1:500 Santa Cruz Cat# sc-515475
Rabbit polyclonal anti-RNASEH2C, WB: 1:500 AbClonal Cat# A13884, RRID:AB_2760737 0067820201
Rabbit polyclonal anti-RNASEH2C, WB: 1:500 Abcam Cat# ab89726, RRID:AB_2042815 GR74866-1
Rabbit polyclonal anti-RNASEH2B, WB: 1:1000 Thermo Fisher Scientific Cat# PA5-59059, RRID:AB_2646610 V13088599
Rabbit polyclonal anti-RNASEH2B, WB: 1:1000 Atlas Antibodies Cat# HPA041469, RRID:AB_2677496 000043725
Rabbit polyclonal anti-phospho-RPA32 (S4/8), WB:1:1000 Bethyl Cat# A300-245A, RRID:AB_210547 7
Rabbit polyclonal anti-RPA32, WB 1:2000
Bethyl Cat# A300-244A, RRID:AB_185548 3
Rabbit polyclonal anti-PRIM1, WB:1000 Proteintech Cat # 10773-1-AP RRID:AB_2237549 00009142
Mouse monoclonal anti-HA.11 Epitope Tag, WB: 1:3000
BioLegend Cat# 901514, RRID:AB_2565336 B272772
Mouse monoclonal anti-V5 tag antibody [SV5-PK1], WB: 1:5000 Abcam (Abcam Cat# ab27671, RRID:AB_471093) GR3337308-16
Sheep polyclonal anti-human RNase
H2 complex, WB: 1:500 Jackson Lab

Secondary Antibodies
Goat Anti-Mouse IgG H&L Cross-Absorbed (Alexa Fluor® 488), IF:1:1000, combing:1:100 ThermoFisher Cat# A-11001, RRID:AB_2534069 632115
Goat Anti-Mouse IgG H&L Cross-Absorbed (Alexa Fluor® 647), combing:1:100 ThermoFisher Cat# A-21235, RRID:AB_2535804 1837146
Goat Anti-Rabbit IgG H&L (Alexa Fluor® 488) ThermoFisher Cat# A-11008, RRID:AB_143165 645151
Goat Anti-Rat IgG H&L Cross-Absorbed (Alexa Fluor® 594), IF: 1:1000, combing: 1:100 ThermoFisher Cat# A-11007, RRID:AB_10561522 2107787
Peroxidase-AffiniPure Goat Anti-Mouse IgG (H + L) antibody, WB: 1:5000 Jackson ImmunoResearch Labs Cat# 115-035-003, RRID:AB_10015289
Peroxidase-AffiniPure Donkey Anti-Sheep IgG (H + L) antibody, WB: 1:5000 Jackson ImmunoResearch Labs Cat# 713-035-003, RRID:AB_2340709

Peroxidase-AffiniPure Goat Anti-Rabbit IgG (H + L) antibody, WB: 1:5000 Jackson ImmunoResearch Labs Cat# 115-035-144, RRID:AB_2307391

Validation

Mouse IgG Santa Cruz Cat# sc-2025 - 3,414 citations

Rabbit IgG Santa Cruz Cat# sc-2027- 12,278 citations

Rabbit IgG Cell Signaling Technology Cat# 2729 - 1,902 citations

Mouse monoclonal anti-alpha-tubulin (clone DM1A) MilliporeSigma Cat# T9026 - 4,263 citations

Mouse monoclonal anti-gamma-H2AX Ser139 (clone JBW301) MilliporeSigma Cat# 05-636 - & manufacturer-verified activity in Jurkat and HeLa cells

Mouse monoclonal anti-biotin Jackson ImmunoResearch Cat# 200-002-211 – 55 citations

Rabbit monoclonal anti-biotin, IF: 1:2000 Bethyl A150-109A 11 – 14 citations

Mouse monoclonal anti-BrdU (B44), combing: 1:10 BD Biosciences Cat# 347580 – 7 citations

Mouse monoclonal anti-Poly (ADP-Ribose) Polymer antibody [10H], WB:1:100 Abcam Cat # ab14459 – 45 citations & manufacturer-verified for IHC in rat tissue

Mouse monoclonal anti-PCNA (PC10), WB: 1:1000 Santa Cruz Cat# sc-56 – 2,861 citations & manufacturer-verified for Western blot and immunofluorescence in CT-116, Raji, HeLa, MOLT-4, NIH/3T3 and KNRK cells

Mouse Monoclonal anti-vinculin, Unconjugated, Clone hVIN-1 MilliporeSigma Cat# V9131 – 1,687 citations

Rat monoclonal anti-BrdU [BU1/75 (ICR1)], combing: 1:20 Abcam Cat# ab6326 – 1,464 citations & manufacturer-verified activity in rat tissue and HeLa and HEK293T cells

Rabbit monoclonal anti-MCM7 (D10A11) XP, WB: 1:1000 Cell Signaling Technology Cat# 3735S – 16 citations & manufacturer-verified activity in HeLa, 3T3, C6, CHO, C2C12, COS cells

Rabbit polyclonal anti-c20orf43 (RTF2), WB: 1:500 Novus Cat# NBP2-30645 R72074 - validated by immunoblot analysis of knockout cells in our hands and manufacturer-verified activity in siRNA-treated HeLa cells

Rabbit polyclonal anti-RTF2, WB: 1:500 Proteintech Cat# 16633-1-AP, RRID: AB_2256547 00074586 - validated by immunoblot analysis of knockout cells in our hands and manufacturer-verified activity in MCF-7

Mouse monoclonal anti-RTF2 (clone OTI1E8), WB: 1:1000 LS Bio Cat# LS-C340588 75213 – 2 citations & validated by immunoblot analysis of knockout cells in our hands and manufacturer-verified activity in COS7 cells and human pancreatic tissue

Rabbit polyclonal anti-GFP Smogorzewska Lab Kotteman et al. – validated by immunoblot analysis of knockout cells in our hands

Rabbit polyclonal anti-GFP Abcam Cat# ab290 – 2,676 citations & manufacturer-verified activity in 293T cells

Mouse monoclonal Anti-GFP, WB: 1:3000 Roche Cat# 11814460001 – 822 citations

Rabbit polyclonal anti-RNASEH2A, WB: 1:500 Proteintech Cat# 16132-1-AP, RRID:AB_2269729 00023264 – 5 citations, validated by immunoblot analysis of knockout cells in our hands, & manufacturer-verified activity in HeLa cells and mouse & human liver tissue, human testis & spleen tissue

Rabbit polyclonal anti-RNASEH2A, WB: 1:500 Abcam Cat# ab83943, RRID:AB_1861175 GR3212381-12 – 3 citations, validated by immunoblot analysis of knockout cells in our hands, & manufacturer-verified activity in HeLa cells

Mouse monoclonal anti-RNASEH2A, WB:1:500 Santa Cruz Cat# sc-515475 – 6 citations, validated by immunoblot analysis of knockout cells in our hands, & manufacturer-verified activity in HeLa, Jurkat, and MCF7 cells

Rabbit polyclonal anti-RNASEH2C, WB: 1:500 AbClonal Cat# A13884, RRID:AB_2760737 0067820201 – manufacturer-verified activity in HeLa and 293T cells

Rabbit polyclonal anti-RNASEH2C, WB: 1:500 Abcam Cat# ab89726, RRID:AB_2042815 GR74866-1 – 3 citations & manufacturer-verified activity in HeLa, 293T, and HepG2 cells

Rabbit polyclonal anti-RNASEH2B, WB: 1:1000 Thermo Fisher Scientific Cat# PA5-59059, RRID:AB_2646610 V13088599 – manufacturer-verified activity in siRNA-treated HeLa cells

Rabbit polyclonal anti-RNASEH2B, WB: 1:1000 Atlas Antibodies Cat# HPA041469, RRID:AB_2677496 000043725 – 1 citation & manufacturer-verified activity in siRNA-treated HEK293T cells

Rabbit polyclonal anti-phospho-RPA32 (S4/8), WB:1:1000 Bethyl Cat# A300-245A, RRID:AB_210547 7 – 165 citations & manufacturer-verified activity in HeLa, AlphaTC1, and BeWo cells and human breast and mouse gut tissue

Rabbit polyclonal anti-RPA32, WB 1:2000 Bethyl Cat# A300-244A, RRID:AB_185548 3 – 40 citations & manufacturer-verified activity in HeLa, HEK293T, U2OS and Jurkat cells and human breast and mouse kidney tissue

Rabbit polyclonal anti-PRIM1, WB:1000 Proteintech Cat # 10773-1-AP RRID:AB_2237549 00009142 – 5 citations & manufacturer-verified activity in K-562 and Sp2/0 cells

Mouse monoclonal anti-HA.11 Epitope Tag, WB: 1:3000
BioLegend Cat# 901514, RRID:AB_2565336 B272772 – 267 citations & manufacturer-verified activity in CHO cells

Mouse monoclonal anti-V5 tag antibody [SV5-PK1], WB: 1:5000 Abcam (Abcam Cat# ab27671, RRID:AB_471093) GR3337308-16 – 108 citations & manufacturer-verified activity in 3T3 cells

Sheep polyclonal anti-human RNase
H2 complex, WB: 1:500 Jackson Lab – 1 citation & validated by immunoblot analysis of knockout cells in our hands

Eukaryotic cell lines

Policy information about cell lines and Sex and Gender in Research

Cell line source(s)

Human: HEK293T, ATCC (CRL-3216)
Human: HEK 293T endogenously GFP-tagged RTF2, this paper
Human: BJ-hTERT-E6/7, male, Smogorzewska Lab
Human: U2OS, female, ATCC (HTB-96)
Human: RPE p53-/-, pRb-/-, PRIM1-AID-mClover, gift from de Lange Lab
Human: HeLa (HeLa Parental, HeLa WT Clone, HeLa RNASEH2A KO Clone), female, gifts from Durocher and Jackson Labs
Human: HCT-116 p53-/- (HCT-116 p53-/- WT Clone, HCT-116 p53-/- RNASEH2A KO Clone), gifts from Durocher and Jackson Labs
Mouse: RTF2 MEFs (Rtf2+/+, Rtf2+/lox, Rtf2-/lox, and Rtf2lox/lox, SV40-immortalized Rtf2-/- clone, p53-/-;Rtf2+/lox, p53-/-;Rtf2-/lox), this paper
KOMP mES cells Rtf2tm1a(KOMP)wtsi KOMP (MGI code: 1913654)

Authentication

RTF2 cell lines (293T endogenously GFP-tagged and all MEF cell lines) were authenticated by genotyping and western blotting. HeLa and HCT116 KO clones, PRIM1-AID-mClover, and conditional
RTF2 MEF cell lines and clones were validated by western blotting, after Cre treatment for conditional cell lines. Confirmation of loss of floxed allele was conducted following each Cre transduction by qPCR or western blotting.

Mycoplasma contamination

Cell lines were tested using PCR-based Mycoplasma detection and were negative.

Commonly misidentified lines
(See ICLAC register)

No commonly misidentified lines were used in this manuscript.

Animals and other research organisms

Policy information about studies involving animals; ARRIVE guidelines recommended for reporting animal research, and Sex and Gender in Research

Laboratory animals

C57BL/6J mice were bred with Rtf2tm1a(KOMP)wtsi mice to generate Rtf2+/stop mice, which were subsequently bred with B6.Cg Tg(ACTFLPe)9205Dym/J to generate Rtf2+/lox mice. Rtf2+/lox mice were bred with B6.FVB/N-Tg(Ella-cre)C5379Lmgd/J mice to generate Rtf2+/- mice. These crosses are outlined in the supplementary data. These mice were intercrossed to determine Mendelian ratios. All mice were bred after reaching 6-8 weeks and were euthanized before 2 years.
All mice were housed in autoclaved bedding in air-filtered cages and autoclaved water. Animal housing rooms are under temperature and humidity control. Rockefeller University provides comprehensive veterinary care utilizing the combined efforts of three full-time veterinarians and six veterinary technicians specially trained in laboratory animal care.

Wild animals

No wild animals were used in this study.

Reporting on sex

Sex of MEF cell lines used in this paper was not determined.

Field-collected samples

No field-collected samples were used in this study.

Ethics oversight

Rockefeller University Institutional Animal Care and Use Committee approved the mouse protocol.

Note that full information on the approval of the study protocol must also be provided in the manuscript.

Flow Cytometry

Plots

Confirm that:

☒ The axis labels state the marker and fluorochrome used (e.g. CD4-FITC).

☒ The axis scales are clearly visible. Include numbers along axes only for bottom left plot of group (a 'group' is an analysis of identical markers).

☐ All plots are contour plots with outliers or pseudocolor plots.

☒ A numerical value for number of cells or percentage (with statistics) is provided.

Methodology

Sample preparation	Exponentially growing cells were labeled with EdU 1 hr prior to collection and fixation. Cell cycle preparation was performed with Click-iT™ EdU Alexa Fluor™ 647 Flow Cytometry Assay Kit per the manufacturer's protocol. Total DNA content was stained with FxCycle™ Violet.
Instrument	BD AccuriTM C6 or BDTM LSR II
Software	FlowJo software was used to analyze Flow Cytometry data
Cell population abundance	Due to the abundance of cells available from cell culture, greater than 20,000 events were captured per sample to yield greater than 10,000 events post-gating strategy.
Gating strategy	Live cell gating: FSC-A (~25k - 225k) versus SSC-A (~25k-150k) based on size<br>Singlet gating: SSC-A versus SSC-W (~50-200k)<br>FSC-A v FSC-W (~75-150k)<br>Edu population (Alexa 647): no Edu control used to gate Edu+ population

☐ Tick this box to confirm that a figure exemplifying the gating strategy is provided in the Supplementary Information.
